# Embedding and Positioning of Two Fe^II^_4_L_4_ Cages in Supramolecular Tripeptide Gels for Selective Chemical Segregation

**DOI:** 10.1002/anie.201900429

**Published:** 2019-05-07

**Authors:** Marion Kieffer, Ana M. Garcia, Cally J. E. Haynes, Slavko Kralj, Daniel Iglesias, Jonathan R. Nitschke, Silvia Marchesan

**Affiliations:** ^1^ Department of Chemistry University of Cambridge Lensfield Road Cambridge CB2 1EW UK; ^2^ Department of Chemical and Pharmaceutical Sciences University of Trieste Via L. Giorgieri 1 34127 Trieste Italy; ^3^ Materials Synthesis Department Jožef Stefan Institute Jamova 39 1000 Ljubljana Slovenia

**Keywords:** chemical separation, host–guest systems, low-molecular-weight gelators, metal–organic cages, self-assembly

## Abstract

An unreported d,l‐tripeptide self‐assembled into gels that embedded Fe^II^_4_L_4_ metal–organic cages to form materials that were characterized by TEM, EDX, Raman spectroscopy, rheometry, UV/Vis and NMR spectroscopy, and circular dichroism. The cage type and concentration modulated gel viscoelasticity, and thus the diffusion rate of molecular guests through the nanostructured matrix, as gauged by ^19^F and ^1^H NMR spectroscopy. When two different cages were added to spatially separated gel layers, the gel–cage composite material enabled the spatial segregation of a mixture of guests that diffused into the gel. Each cage selectively encapsulated its preferred guest during diffusion. We thus present a new strategy for using nested supramolecular interactions to enable the separation of small molecules.

Gels share properties of solids and liquids, and exhibit stimuli responsiveness,^1^ which enables applications that include environmental remediation^2^ and cargo delivery.^3^ The incorporation of metal complexes and hollow metal–organic cages^4^ (MOCs) into gels^5^ can alter the responsiveness of the material to stimuli,^6^ thus endowing it with useful properties comprising fluorescence^7^ and self‐healing.^8^ Chemically modified MOCs have been reported to act as nodes within gels^9^ and polymers,^10^ with linkages between cages formed by polymeric chains^11^ or binders.^12^ However, we envisaged that covalent linkages between the hollow architectures and the gel matrix^13^ may be unnecessary for embedding MOCs in a supramolecular gel matrix. In addition, we hypothesized that the ability of MOCs to selectively encapsulate molecular cargoes^14^ could allow us to prepare cage‐containing gels that can separate molecules spatially,^15^ by extracting guests from solution as they diffuse through a gel.

This strategy requires the identification of a suitable gel matrix that is compatible with MOCs. Minimalistic peptides^16^ are readily obtained, biocompatible materials^17^ that form fibrillar gels under a wide variety of conditions. Although the resulting nanostructures often display size heterogenerity due to hierarchical assembly,^18^ the addition of a second component into the supramolecular system can limit fibril bundling and allow a more homogeneously defined nanomorphology.^19^ Peptides can also display recognition motifs for cell internalization,^20^ and enzyme mimicry for catalysis,^21^ and thus are attractive components for adaptive supramolecular materials. We envisaged a hybrid peptide gel could embed MOCs for functional guest binding through hierarchical self‐assembly. Such systems are capable of separating and immobilizing different cages within distinct regions of a gel, but within a single solvent phase without requiring covalent linkages to be made between MOCs and gelators. The high tunability of the process lies in the absence of synthetic modification needed on either the MOCs or the peptide, making it an extremely versatile strategy. The spatially separated cages may thus bind distinct guests, allowing these guests to be segregated within defined parts of the gel, and thus separated from a mixture by selective encapsulation.

Cyclic peptides that feature alternating d‐ and l‐amino acids have been shown to self‐assemble into nanotubes and hydrogels.^22^ However, prediction of the gel‐formation behavior for smaller, acyclic minimalistic peptides is difficult.^23^ Tripeptides of syndiotactic l‐d‐l stereochemistry have been shown only recently to adopt an amphipathic conformation, with side chains in an isotactic configuration that enables their long‐range self‐organization into gel‐forming fibers. An exception to this design rule is l‐Phe‐d‐Ala‐l‐Phe, which does not meet the expected requirements of hydrophobicity and steric hindrance and was thus not observed to form a gel.^24^ We hypothesized that the addition of a *p*‐aminobenzoyl moiety at the N‐terminus could address both issues to yield a new gelator, which would also be potentially useful in cases where the presence of a primary aliphatic amine is undesirable. Indeed, a free N‐terminus could result in unwelcome substitution of MOC amine subcomponents and reduce MOC stability, whereas an aniline substituted with an electron‐deficient amide group would not be expected to substitute more electron‐rich aniline residues incorporated into the periphery of a cage.^25^

The tripeptide (*p*‐aminobenzoyl)‐l‐Phe‐d‐Ala‐l‐Phe‐NH_2_ (Figure 1) was thus synthesized and probed for gelation in various solvents, among which CH_3_CN was chosen as the most suitable choice for stable gels and cages (see Table S2 in the Supporting Information). Two MOCs (Figure 1) were prepared^25^ to encapsulate different guests in CH_3_CN; MOC **1** binds trifluoroacetate (TFA^−^) moderately and perrhenate (ReO_4_^−^) strongly, as evidenced by ^1^H and ^19^F NMR spectroscopy (see Figures S13–S17 in the Supporting Information), whereas MOC **2** encapsulates fluoroadamantane (FA; see Figures S18 and S19).

**Figure 1:**
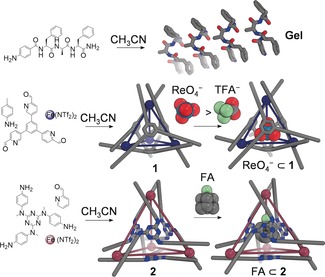
a) Self‐assembly of (*p*‐aminobenzoyl)‐l‐Phe‐D‐Ala‐l‐Phe‐NH_2_ into gel‐forming fibrils. b,c) Synthesis of cages **1** and **2** by subcomponent self‐assembly and encapsulation of ReO_4_^−^ or TFA^−^ in **1** and FA in **2**.

The tripeptide formed gels (50 mm, 2.5 wt %) that incorporated either of the cages at concentrations up to 5 mm (10:1 tripeptide:cage in this case). The gels were visibly colored, even at a MOC concentration of 0.1 mm (Figure 2 a,b); for analyses, MOCs were employed at 1 or 5 mm and the peptide at 50 mm to facilitate detection. Energy‐dispersive X‐ray (EDX) spectra showed homogenous distribution of iron (Figure 2 c,d; see also Table S4 and Figures S30–S34). The cages were inferred to remain intact and evenly distributed within the gels, as confirmed by the other techniques noted below. Rheology revealed that both cages affected the gel viscoelastic properties, thus providing a means to fine‐tune gelation kinetics, stiffness, and resistance to applied stress (Figure 2 e,f; see also Figures S21–S23). Frequency sweeps indicated that both the elastic (G′) and the viscous (G′′) moduli were independent of the applied frequency, and G′>G′′ (see Table S3), thus confirming gelation. In particular, gelation kinetics revealed a two‐stage process with a lag phase only in the presence of either MOC, thus suggesting a longer nucleation phase relative to the peptide alone.^26^

**Figure 2:**
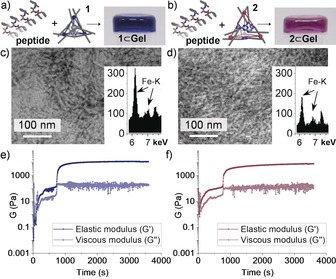
a,b) Peptide and either cage **1** or **2** yield **1**⊂**Gel** and **2**⊂**Gel**, respectively. c,d) TEM images show the fibrils of the gels and EDX spectra (insets) show the presence of Fe from MOCs at 1 mm in **1**⊂**Gel** (c) and **2**⊂**Gel** (d). Scale bar=100 nm. e,f) Gelation kinetics depict G′ (dark blue and dark purple) and G′′ (light blue and light pink) for **1**⊂**Gel** (e) and **2**⊂**Gel** (f).

Transmission electron microscopy (TEM) was used to investigate the morphology of the three different gels, that is, the peptide alone, and the hybrids **1**⊂**Gel** and **2**⊂**Gel** (Figure 2 c,d; see also Figures S25–S29). The peptide yielded a colorless, transparent gel containing a matrix of homogeneous thin fibrils of average diameter (9.8±1.7) nm (*n=*100; see Figure S25). Either cage **1** or **2** at a concentration below 1 mm did not significantly alter the fibril diameter ((8.9±1.9) nm for **1**⊂**Gel** and (9.0±2.1) nm for **2**⊂**Gel**; see Figures S26 and S27). However, at the higher MOC concentration of 5 mm, **1**⊂**Gel** displayed thinner fibrils with a diameter of (6.5±2.0) nm (see Figure S28), while **2**⊂**Gel** showed instances of amorphous aggregates after 5 days, along with fibrils that were (11.7±1.9) nm wide (see Figure S29).

MOC stability in the gels was probed by ^1^H and ^19^F NMR spectroscopy. In gels incorporating TFA^−^⊂**1** or FA⊂**2**, MOC integrity was evidenced by the absence of NMR resonances associated with free cage subcomponents (e.g., the aldehyde peak at 10 ppm); free guests were also not observed (see Section 5.1 in the Supporting Information). The gel stabilized the MOCs at TFA^−^ concentrations that resulted in their decomposition, aggregation, and precipitation when free in solution (see Figures S36–S39). Intact **1** and **2** were observed by ESI‐MS after dilution and filtration of **1**⊂**Gel** and **2**⊂**Gel** (see Figures S44 and S45). Minor substitution (below 6 %) was observed after 1 week when **1** (5 mm) was combined with (*p*‐aminobenzoyl)‐l‐Phe‐OMe (50 mm), with the extent of substitution limited by the electron deficiency of the aniline. We hypothesized that, similarly, the peptide substituted only minor amounts of the *p*‐toluidine of MOC **1** in the gels (see Section 5.1.3 in the Supporting Information). UV/Vis (see Figure S24) and Raman spectroscopic analyses (see Figure S35) confirmed MOC integrity in **1**⊂**Gel** and **2**⊂**Gel**. Raman^27^ imaging of **1**⊂**Gel** and **2**⊂**Gel** revealed a homogeneous distribution of MOCs in the gels at the microscale (Figure S35 D,E). Circular dichroism indicated that the peptide conformation was not changed by either MOC (see Figure S20).

The host–guest chemistry of **1**⊂**Gel** and **2**⊂**Gel** was studied by ^1^H and ^19^F NMR spectroscopy over time (Figure 3). We infer the rate of guest uptake by cages in the gel to depend upon both guest diffusion through the sample, purely controlled by kinetics, and guest encapsulation, which is influenced by both thermodynamic (binding affinity) and kinetic factors (guest uptake rate). Indeed, the rate of guest uptake in gels was substantially slower than in solution owing to the kinetic contributions (diffusion in gels for guest uptake), while the binding affinity most likely remained unchanged (Figure 3).

**Figure 3:**
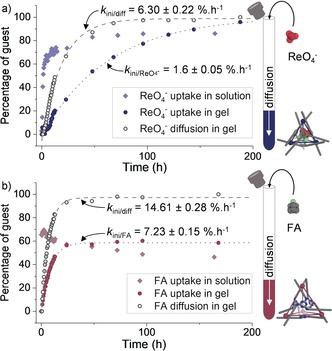
a) ReO_4_^−^ uptake kinetics by **1** in solution (light‐blue diamonds) and by **1**⊂**Gel** (dark‐blue dots) and diffusion of ReO_4_^−^ in **1**⊂**Gel** (black circles). b) FA uptake kinetics by **2** in solution (pink diamonds) and by **2**⊂**Gel** (purple dots) and diffusion of FA in **2**⊂**Gel** (black circles). Dotted lines represent the asymptotic fitting from which the initial rates (*k*_ini_) were deduced.

We hypothesize that more ReO_4_^−^ was encapsulated in **1**⊂**Gel**, relative to **1** in solution, because of binding of TFA^−^ anions by the gel fibrils, thus reducing the availability of TFA^−^ for competitive encapsulation. This interaction between anions and fibrils was evidenced by broadening of the ^19^F NMR signals corresponding to the TFA^−^ in the gel, as compared to the solutions containing either **1** or **2** alone. No significant changes in the proportion of FA encapsulated in **2** were observed between **2**⊂**Gel** and **2** in solution, as TFA^−^ is not a competing guest for **2**.

Fitting the guest‐uptake curves to an asymptotic exponential model enabled calculation of the initial rate of guest uptake or diffusion (see Section 5.3 in the Supporting Information), thus allowing quantitative comparison between samples. The diffusion of the guests (FA or ReO_4_^−^) in **1**⊂**Gel** ((6.30±0.22) % h^−1^ and (5.44±0.20) % h^−1^, respectively, for the initial rates of diffusion) was slower than in **2**⊂**Gel** ((14.61±0.28) % h^−1^ for FA). In **1**⊂**Gel**, the rates of diffusion for ReO_4_^−^ and FA were similar. These observations suggest that the rate of diffusion of small molecules within these gels is mainly influenced by the structure of the gel network, as observed by TEM (see Section 4.6 in the Supporting Information), rather than by the chemical structure of the diffusing species. We inferred that the difference in nanostructures between gels, such as thinner fibrils with higher surface area, led to greater physisorption and therefore slower diffusion of guests.

A fivefold difference between the initial rate of guest uptake for **1**⊂**Gel** and **2**⊂**Gel** ((1.60±0.05) % h^−1^ and (7.23±0.15) % h^−1^, respectively) was observed owing to the different properties of the hybrid MOC⊂Gel. Thus, temporal control of the encapsulation of small guest molecules could be achieved for this system.

The diffusion of larger species, such as the MOCs themselves, was shown to be much slower than that of the smaller guests. The initial rate of diffusion of the MOCs was four and seven times slower, respectively, in **1**⊂**Gel** and **2**⊂**Gel** ((1.31±0.20) % h^−1^ and (2.11±0.11) % h^−1^), meaning that the MOCs remained localized in the gel matrix on a timescale that allowed the smaller guests to diffuse through the sample. The system therefore allowed for MOC segregation in separate gel layers, as required for selective guest separation.

With guest separation in mind, we designed a three‐layered system composed of **1**⊂**Gel** and **2**⊂**Gel**, separated by a buffer layer of peptide gel devoid of MOCs (Figure 4 a). The presence of each MOC in their respective layers was confirmed by spatial mapping of the sample by slice‐selective ^1^H NMR spectroscopy (Figure 4 b).^28^ The gray regions in Figure 4 b detail the spatial partitioning of the proton signals corresponding to cages **1** and **2** within the gel matrix. ^1^H NMR signals assigned to **1** (blue spectrum) were only found in layer 1, and signals for **2** (purple spectrum) were only found in layer 2. The observation of color leaching into the buffer layer was attributed to the strong visible absorbance of MOCs even at trace concentrations (Figure 2 a,b). ^1^H NMR signals of the peptide only were detected there, indicating that the MOCs remained segregated mostly in their original layers and were not present at concentrations above the NMR detection threshold in the buffer layer (see Section 5.4 in the Supporting Information).

**Figure 4:**
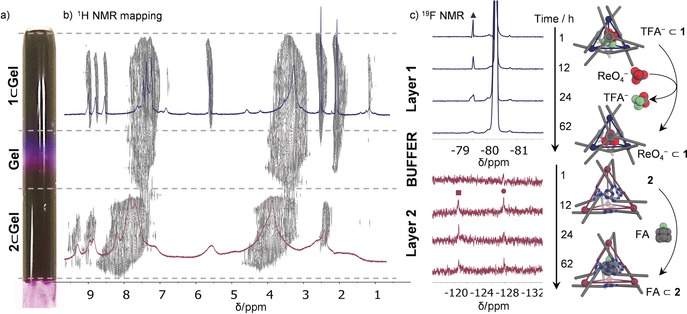
a) Photograph of the three‐layered gel and b) 2D mapping of the ^1^H NMR spectra for the three‐layered gel, showing the presence of MOC **1** in layer 1 (**1⊂Gel**), the peptide alone in the buffer gel layer, and MOC **2** in layer 2 (**2⊂Gel**). c) ^19^F NMR spectra of layer 1 (top, blue) and layer 2 (bottom, purple) showing, respectively, the decrease in the peak for encapsulated TFA^−^ (▴) and the increase in both the encapsulated (▪) and free FA (•) over time after the addition of mixed FA and ReO_4_^−^ (1 equiv each).

After the three‐layered gel formation, a mixture of tetrabutylammonium ReO_4_^−^ and FA in CD_3_CN was added on top of the gel, and the diffusion of the guests through the different gel layers was followed by slice‐selective ^19^F NMR spectroscopy (Figure 4 c). Owing to the faster diffusion and uptake of FA in **2**⊂**Gel** relative to that of ReO_4_^−^ in **1**⊂**Gel,** maximum guest encapsulation was achieved in 12 h for the former, and in 62 h for the latter (Figure 4 c). Afterwards, each MOC‐encapsulated guest could be observed specifically in its respective layer. While free FA was observed in both layers, most likely in similar proportions, the presence of both FA⊂**2** and free FA in layer 2 signaled a clear enrichment of the compound in this layer. Over 80 % of the encapsulated TFA^−^ was displaced by ReO_4_^−^ from the cavity of MOC **1**, and the remaining ReO_4_^−^ was inferred to be spread across both layers, thus resulting in an estimated enrichment up to a ratio 9:1 of this compound in layer 1 as compared to layer 2 (Figure 4 c; see also Figures S72 and S73). The potential of this type of system for selective chemical separation was thus demonstrated. The investigation of MOCs with greater binding affinity for more technologically relevant guests will be explored next, to further optimize the extent of separation achievable by these systems.

In conclusion, we have demonstrated the formation of hybrid MOC⊂gel nanostructured materials made of a self‐assembled tripeptide and one of two distinct Fe_4_L_4_ cages. These gels allow for the spatial separation of chemically distinct phases from one‐solvent systems. The hierarchically nested supramolecular assemblies enable selective chemical segregation by means of guest encapsulation. The gel nanostructure is influenced by the presence of the cages, thus providing new means to tune the diffusion kinetics and, as a consequence, the uptake of small molecules in the embedded MOCs. Future efforts will focus upon the extension of this chemical platform to biocompatible materials able to perform time‐controlled guest release.

## Conflict of interest

The authors declare no conflict of interest.

## Supporting information

As a service to our authors and readers, this journal provides supporting information supplied by the authors. Such materials are peer reviewed and may be re‐organized for online delivery, but are not copy‐edited or typeset. Technical support issues arising from supporting information (other than missing files) should be addressed to the authors.

Supplementary
