# Supplementary material for: Embedding and Positioning of Two FeII 4L4 Cages in Supramolecular Tripeptide Gels for Selective Chemical Segregation
Source: Angew Chem Int Ed Engl. 2019 May 7;58(24):7982–6. doi: 10.1002/anie.201900429 (PMC6563161; doi:10.1002/anie.201900429)
Supplement: Supplementary file 1 — Supplementary [file ANIE-58-7982-s001.pdf]

Angewandte Chemie
Eine Zeitschrift der Gesellschaft Deutscher Chemiker

Supporting Information

Embedding and Positioning of Two $Fe^{II}{}_4L_4$ Cages in Supramolecular Tripeptide Gels for Selective Chemical Segregation

Marion Kieffer[+], Ana M. Garcia[+], Cally J. E. Haynes, Slavko Kralj, Daniel Iglesias, Jonathan R. Nitschke, and Silvia Marchesan**

anie_201900429_sm_miscellaneous_information.pdf

Table of Contents

Table of Contents 2
1) Materials and methods 3
1.1) General 3
1.2) Mass spectrometry (MS) 3
1.3) Nuclear Magnetic Resonance (NMR) 3
1.4) LC-MS analysis 3
1.5) Circular Dichroism. 3
1.6) Oscillatory rheometry. 3
1.7) UV analysis. 4
1.8) Transmission electron microscopy (TEM) and energy-dispersive X-ray spectroscopy (EDXS). 4
1.9) Raman Spectroscopy. 4
2) Synthesis 4
2.1) Peptide *p*-aminobenzoyl-L-Phe-D-Ala-L-Phe-NH_2 4
2.2) Cage 1 7
2.3) Cage 2 8
2.4) Subcomponent (*p*-aminobenzoyl)-L-Phe-OMe C and cage 3 9
3) Host-guest complexes 10
3.1) $TFA^- \subset 1$ 10
3.2) $ReO4^- \subset 1$ in the presence of 20 eq. of TFA^- 11
3.3) FA ⊂ 2 (FA = Fluoroadamantane) 13
4) Gels formation and characterization 14
4.1) General preparation of peptide gel 14
4.2) General preparation of peptide/cage gel systems (1⊂Gel and 2⊂Gel) 14
4.3) Circular Dichroism. 14
4.4) Oscillatory rheometry. 15
4.5) UV analysis. 16
4.6) Transmission electron microscopy. 17
4.7) Raman spectroscopy. 20
5) Host-guest chemistry in gels 21
5.1) Stability of cages 1 and 2 21
5.1.1) Stability of **1** and **2** in solution 21
5.1.2) Stability of **1** and **2** in the gel 23
5.1.3) Subcomponent exchange in **1** 26
5.2) Guest uptake in cages 1 and 2 27
5.2.1) Guest uptake in solution 27
5.2.2) Guest uptake in gel 29
5.3) Diffusion of compounds through the gel 32
5.3.1) Diffusion of respective guest 32
5.3.2) Diffusion of FA in **1**⊂ Gel 34
5.3.3) Cage diffusion in the gels 36
5.3.4) Summary of initial uptake and diffusion rates 39
5.4) Spatial segregation of cages and guest separation 39
References: 42
Author Contributions 43

1) Materials and methods

1.1) General

2-chlorotrityl chloride resin, *O*-benzotriazole-*N*,*N*,*N*,*N′*-tetramethyluronium-hexafluoro-phosphate (HBTU), 1-hydroxy-7-azabenzotriazole (HOAt) and the Fmoc-protected amino acids were purchased from GL Biochem (Shanghai) Ltd. (Shanghai, China). All other reagents and solvents were purchased from commercial suppliers and were used as supplied without purification unless stated otherwise. Iron(II)bis(trifluoromethane)sulfonimide, 5,5',5''-(benzene-1,3,5-triyl)tripicolinaldehyde **A** and *N2*,*N4*,*N6*-tris(4-aminophenyl)-*N2*,*N4*,*N6*-trimethyl-1,3,5-triazine-2,4,6-triamine **B** were prepared following literature procedures.[1]

1.2) Mass spectrometry (MS)

Low resolution electrospray mass spectra (LR ESI-MS) were obtained on a Micromass Quattro LC (cone voltage 10-30 eV, desolvation temp. 313 K, ionization temp. 313 K) infused from a Harvard Syringe Pump at a rate of 10 *µL* per minute. High resolution electrospray mass spectra (HR ESI-MS) were obtained on a Thermofisher LTQ Orbitrap XL hybrid ion trap mass spectrometer.

1.3) Nuclear Magnetic Resonance (NMR)

NMR spectra were recorded on a Bruker 400 MHz Avance III HD Smart Probe, Bruker 500 MHz AVIII HD Smart Probe Spectrometer and Bruker 500 MHz DCH Cryoprobe Spectrometer. Proton chemical shift (δ) values are reported in ppm relative to the solvent residual acetonitrile peak (δ = 1.94 ppm). Fluorine chemical shift (δ) values are reported in ppm relative to the internal reference peak of hexafluorobenzene in acetonitrile (δ = −164.90 ppm). Carbon chemical shift (δ) values are reported in ppm relative to the solvent residual peak (δ = 1.32 and 118.26 ppm for CH_3CN). Coupling constants (*J*) are reported in Hertz (Hz) and the signal multiplicities are described as: s (singlet), d (doublet), t (triplet), m (multiplet) and b (broad).
NMR spectra of gels and slice selective experiments were recorded on a Bruker 500 MHz AVIII HD Smart Probe Spectrometer using the previously reported pulse and au program designed by Duncan Howe for the Chemistry Department of the University of Cambridge.[2]

1.4) LC-MS analysis

LC-MS data was acquired on an Agilent 6120 LC-MS system with a C-18 analytical column (Zorbax SB-C18 Rapid Resolution HT 2.1x50 mm, particle size: 1.8 microns), flow 0.5 ml/min. The gradient used consisted of CH_3CN /H_2O with 0.1% formic acid with the following program: t = 0-2 min. 5% CH_3CN; t = 10 min. 95% CH_3CN; t = 12 min. 95% CH_3CN (peptide t_R= 8.4 min).

1.5) Circular Dichroism.

A 0.1 mm quartz cell was used in a Jasco J-815 Spectropolarimeter, with 1 s integrations, 1 accumulation and a step size of 1 nm with a bandwidth of 1 nm. The CD signal was monitored from 210 to 700 nm at 25 °C (Peltier).

1.6) Oscillatory rheometry.

Dynamic time sweep rheological analyses were performed on a Malvern Kinexus Ultra Plus Rheometer (Alfatest, Milan, Italy) with a 20 mm stainless steel parallel plate geometry. The system was kept at 25 °C using a Peltier temperature controller. Each gel was prepared *in situ* and immediately analyzed with a gap of 1 mm. Time sweeps were recorded for 1 hour, using a frequency of 2 Hz and a controlled stress of 2 Pa. After 1 hour, frequency sweeps were recorded from 0.1 to 10 Hz using a controlled stress of 2 Pa. Finally,

stress sweeps were recorded using a frequency of 2 Hz until the breaking point for every gel, recognizable by the inversion of G' and G'' values. Each analysis was repeated at least 3 times.

1.7) UV analysis.

UV visible spectra were recorded on a PerkinElmer Lambda 35 UV-Vis spectrophotometer using a 0.1 mm path length cuvette. The samples were freshly prepared for the analysis and the measurements were performed at room temperature.

1.8) Transmission electron microscopy (TEM) and energy-dispersive X-ray spectroscopy (EDXS).

TEM analyses were performed on JEM 2100 (Jeol, Japan) equipped with an Oxford Instruments INCS energy-dispersive X-ray spectroscopy (EDXS) detector operated at 100 kV. TEM grids (copper-grid-supported lacey carbon film) were first exposed to the UV-ozone cleaner (UV-Ozone Procleaner Plus) for 10 mins to make the grid surface more hydrophilic. Five days aged gels were precisely deposited on a TEM grid, dried for 15 mins at room temperature, and contrasted by aqueous tungsten phosphate solution (pH 7.4). TEM micrographs were acquired on at least 15 different spots on TEM grid accompanying corresponding EDXS analyses. The average size or cross-section diameter of the nanostructures was determined by averaging at least 100 individual nanostructures.

1.9) Raman Spectroscopy.

Raman analysis was acquired in an Invia Renishaw microspectrometer (50) equipped with He-Ne laser at 532 nm. The laser was focused using a 50 x microscope and the power of the laser was set as required for the sample. At least 10 spectra per sample were acquired to assure the homogeneity of the samples. For the characterization of the powders, a small amount of sample was deposited on a glass microscope slide. For the gels, a small piece of gel was deposited on quartz and open air dried before the analysis. Sample preparation was analogous for Raman imaging. An area of around 625 µm was mapped over 676 points. The exposure time was 1 s and the power of the laser was set at 0.08 mW. The data was normalized to the maximum and the relative intensity of the peak at 1448 and 1472 cm^{-1} were plotted for cage **1** and cage **2**, respectively.

2) Synthesis

2.1) Peptide *p*-aminobenzoyl-L-Phe-D-Ala-L-Phe-NH_2

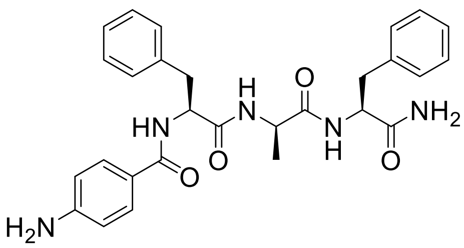

p-aminobenzoyl-L-Phe-D-Ala-L-Phe-NH_2 was synthesized following Fmoc-based SPPS under dry and inert atmosphere. Briefly, the swelling of the resin (2-chlorotrytil chloride, 10 g) was done in dichloromethane (40 mL). Then $SOCl_2$ (1 mL) was added, and the reaction was shaken under an argon flow for 1 h. After that, the resin was washed with DMF (2 x 30 mL) and dicloromethane (2 x 30 mL). Next, a solution of Fmoc-Rink amide linker (5.4 g, 10 mmol), DIPEA (9 mL) in DMF/dichloromethane (2:1) was added to the resin, and the reaction was stirred for 1.5 h. Then, methanol (4 mL) was added and it was shaken for 5 minutes following by washes with DMF (3 × 30 mL) and dicloromethane (3 × 30 mL). For the deprotection, piperidine 20% in DMF (10 mL) was added to the reactor and was stirred at room temperature (2 x 10 minutes). The reaction mixture was washed with DMF and dichloromethane. For the first coupling, a mixture of Fmoc-L-Phe-OH (7.3 g, 18.8 mmol), HBTU (5.7 g, 15.0 mmol), HOAt (2.0 g, 15.0 mmol) and DIPEA 1 M in DMF (15 mL) in DMF was ultrasonicated until the solution was clear, and was added to the reactor. The coupling was shaken at room temperature for 1.5 h. Then, the resin was washed and deprotected as in the previous step. The coupling and deprotection of the followings amino acids (D-Ala and L-Phe) were done exactly the same way as the first coupling, by using Fmoc-D-Ala-OH (5.9 g, 18.8 mmol) for the second coupling and Fmoc-L-Phe-OH (7.3 g, 18.8 mmol) for the third one. For the introduction of *p*-aminobenzoyl motif, a coupling was performed under the same

conditions by using Boc-protected p-aminobenzoic acid instead of a Fmoc-protected amino acid. Eventually, the peptide was cleaved from the resin by shaking 2 hours in the presence of a solution of TFA/dichloromethane/H_2O/triisopropylsilane (47.5/47.5/2.5/2.5) (100 mL). The solution was drained from the reactor, and the solvent was evaporated under air flow. The remaining oil was dissolved in a mixture of acetonitrile/H_2O (containing 0.05% of TFA), and then purified by reverse-phase HPLC (Agilent Technologies, Santa Clara, CA, USA). The HPLC Agilent 1260 Infinity system was equipped with a preparative gradient pump (1311B), semipreparative C-18 column (Kinetex, 5 microns, 100 Å, 250 mm x 10 mm, Phenomenex, Torrance, CA, USA), autosampler (G1329B), and Photodiode Array detector (G1315C). The following HPLC method was used for the purification of the peptide: t = 0–2 min, 25% CH_3CN; t = 14 min, 80% CH_3CN; t = 16 min, 95% CH_3CN; t = 17 min, 95% CH_3CN. The compound was then freeze-dried to yield the corresponding peptide as a white fluffy powder. Peptide identity was verified by ESI-MS, ^{1}H-NMR, and ^{13}C-NMR. ^{1}H and ^{19}F NMR were used to assess the amount of residual TFA^{-} per peptide. Integration of protons and fluorine signals against a reference capillary of $C_6F_3H_3$ showed the presence of 2 equivalents of TFA^{-} per equivalent of peptide.

^{1}H NMR (400 MHz, DMSO-d_6) δ 8.19 (d, J = 7.5 Hz, 1H_d), 8.10 (dd, J = 8.2, 1.8 Hz, 2$H_{j,m}$), 7.55 (d, J = 8.8 Hz, 2H_c), 7.36 (bs, 2H_s), 7.29 – 7.07 (m, 10$H_{g,h,i,p,q,r}$), 6.56 (d, J = 8.8 Hz, 2H_b), 4.75 (bs, 2H_a), 4.52 (ddd, J = 9.1, 7.5, 5.9 Hz, 1H_e), 4.35 (ddd, J = 10.6, 8.8, 4.1 Hz, 1H_n), 4.16 (q, J = 7.3 Hz, 1H_k), 3.11 – 2.91 (m, 3$H_{f,o}$), 2.75 (dd, J = 13.7, 10.6 Hz, 1H_o), 0.91 (d, J = 7.0 Hz, 3H_l).

^{13}C NMR (100 MHz, DMSO-d_6) δ 173.4, 172.1, 171.8, 167.7 (4 x CO); 151.1, 138.1, 137.5, 129.3, 129.2, 129.1, 128.4, 128.2, 127.9, 126.6, 126.4, 121.6, 117.3, 113.5 (Ar); 56.4, 55.0, 48.9 (3 x αC); 37.0, 36.7, 16.2 (3 x βC).

ESI-MS: m/z calculated for M = 501.2, observed positive mode $[M + H]^{+}$ = 502.2 and negative mode $[M - H]^{-}$ = 500.2

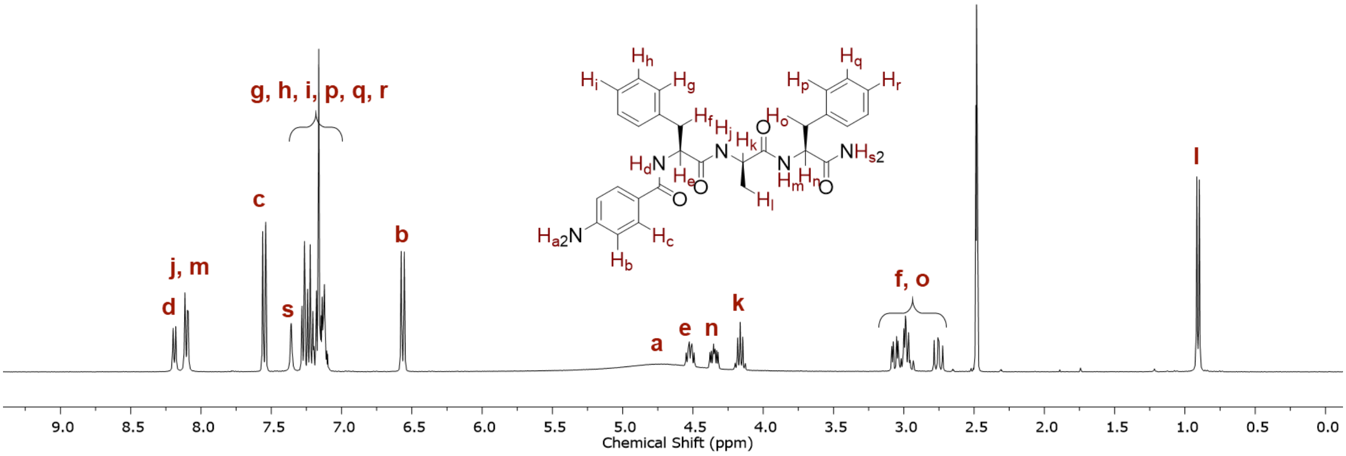

Figure S1: ^{1}H NMR spectrum (400 MHz, 298 K, DMSO-d_6) of the peptide

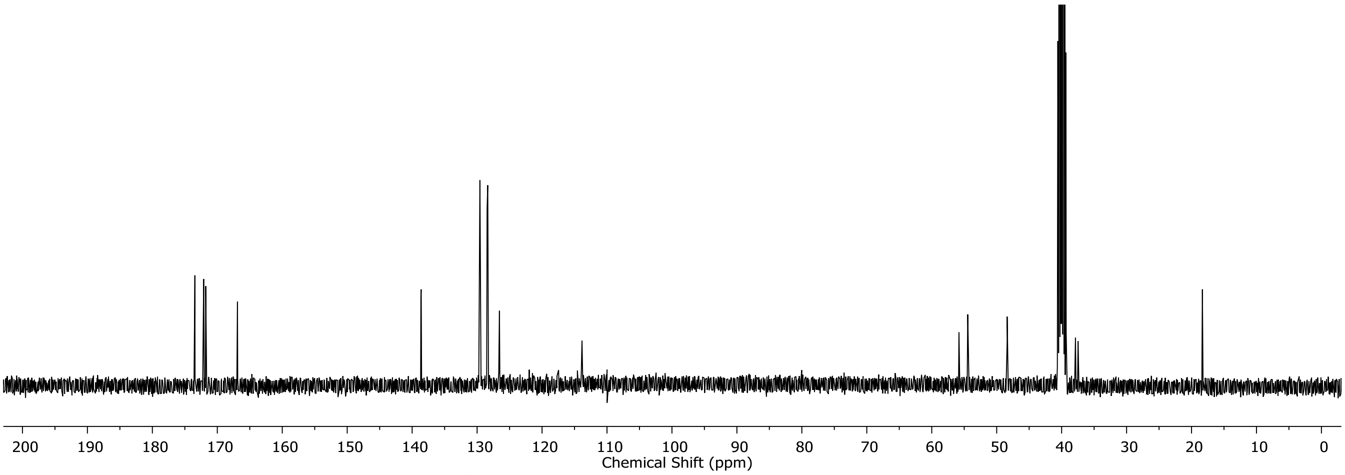

Figure S2: ^{13}C NMR spectrum (101 MHz, 298 K, DMSO-d_6) of the peptide.

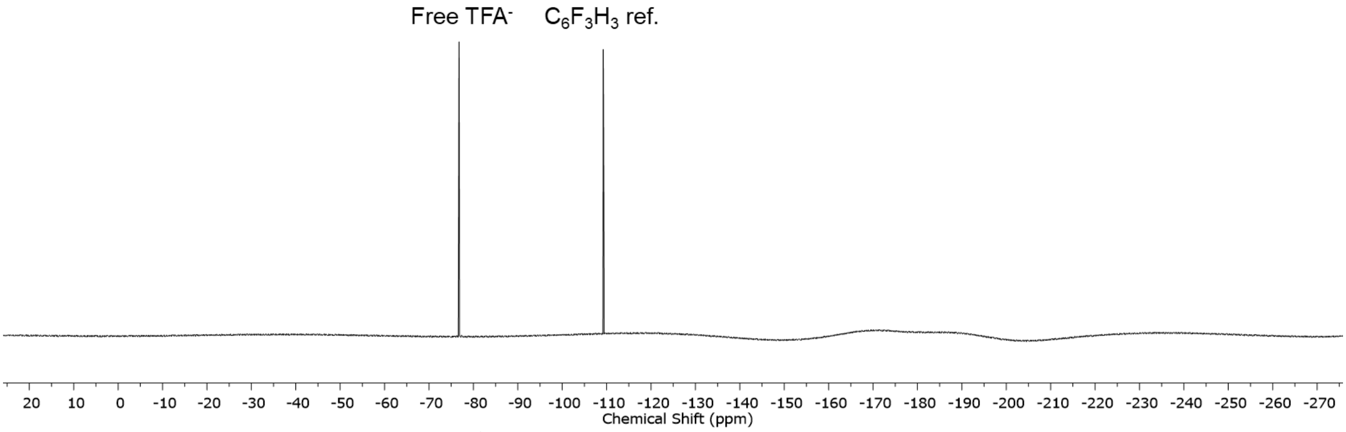

Figure S3: ^{19}F NMR spectrum (471 MHz, 298 K, CD_3CN) of the peptide.

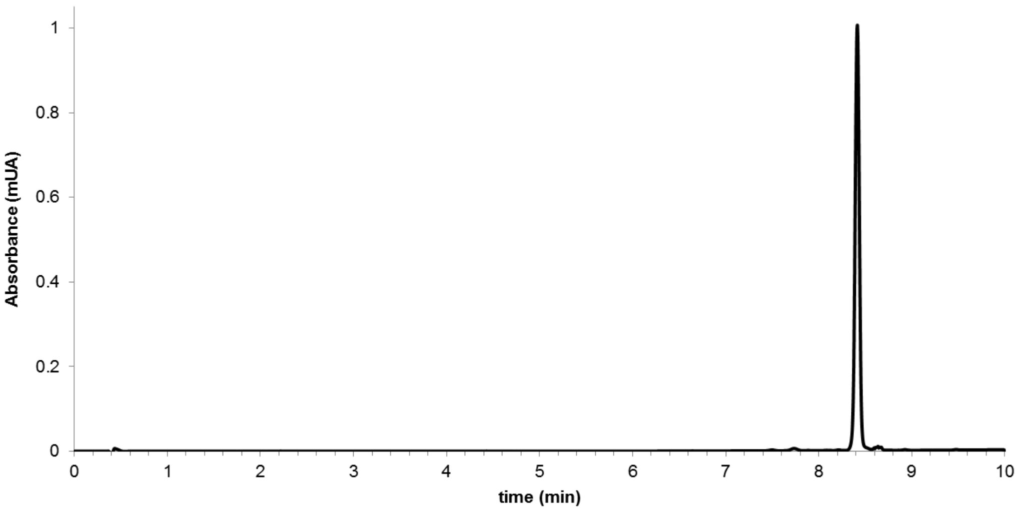

Figure S4: LC trace of the peptide (λ = 254 nm). Method: t = 0, 95% water (+0.1% CF_3COOH) and 5% MeCN (+0.1% CF_3COOH); t = 10 min, 5% water (+0.1% CF_3COOH) and 95 % MeCN (+0.1% CF_3COOH). Flow: 0.5 ml/min. (t_R= 8.4 min).

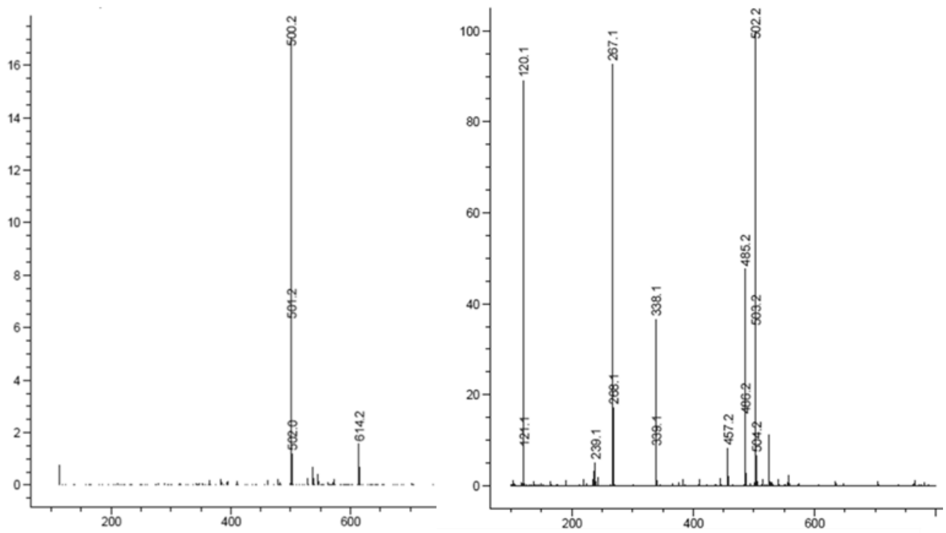

Figure S5: ESI-MS spectra of the peptide in negative (left) and positive (right) ion mode.

2.2) Cage 1

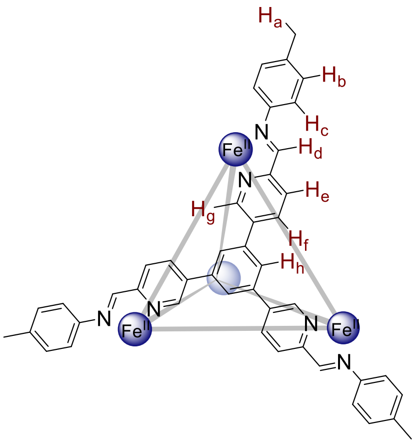

5,5',5''-(benzene-1,3,5-triyl)tripicolinaldehyde **A** (40.0 mg, 0.1018 mmol, 1.0 equiv.) and *p*-toluidine (32.7 mg, 0.3054 mmol, 3.0 equiv.) were dissolved in 10.0 mL of CH_3CN in a sealed 25 mL round bottom flask. The solution was degassed with N_2 for 10 min after which $Fe(NTf_2)_2$ (70.0 mg, 0.1018 mmol, 1.0 equiv.) was added. The solution was degassed for an additional 10 min. The solution was heated at 50 °C for 18 h. The dark blue solution was then cooled and concentrated under a flow of nitrogen. Addition of 80 mL of diethyl ether precipitated the compound into a dark blue solid. The solid was separated by centrifugation and washed with diethyl ether (2 × 80 mL). The solid was then dried under a flow of nitrogen.

^{1}H NMR (500 MHz, acetonitrile-*d*$_3$) δ 8.95 (s,12H, H_d), 8.81 (d, *J* = 8.3, 12H, H_e), 8.55 (dd, *J* = 8.3, 2.0 Hz, 12H, H_f), 7.43 (s, 12H, H_h), 7.14 (d, *J* = 8.1 Hz, 24H, H_b), 7.11 (d, *J* = 2.0 Hz, 12H, H_g), 5.51 (d, *J* = 8.0 Hz, 24H, H_c), 2.41 (s, 36H, H_a).
^{19}F NMR (471 MHz, acetonitrile-*d*$_3$) δ -80.45.
LR-ESI-MS [charge, calculated mass]: *m/z* = 1422.2 [**1**$(NTf_2)_5^{3+}$, 1422.4], 996.7 [**1**$(NTf_2)_4^{4+}$, 996.8], 741.3 [**1**$(NTf_2)_3^{5+}$, 741.4], 571.0 [**1**$(NTf_2)_2^{6+}$, 571.2], 449.4 [**1**$(NTf_2)^{7+}$, 449.5], 358.2 [**1**$^{8+}$, 358.3].

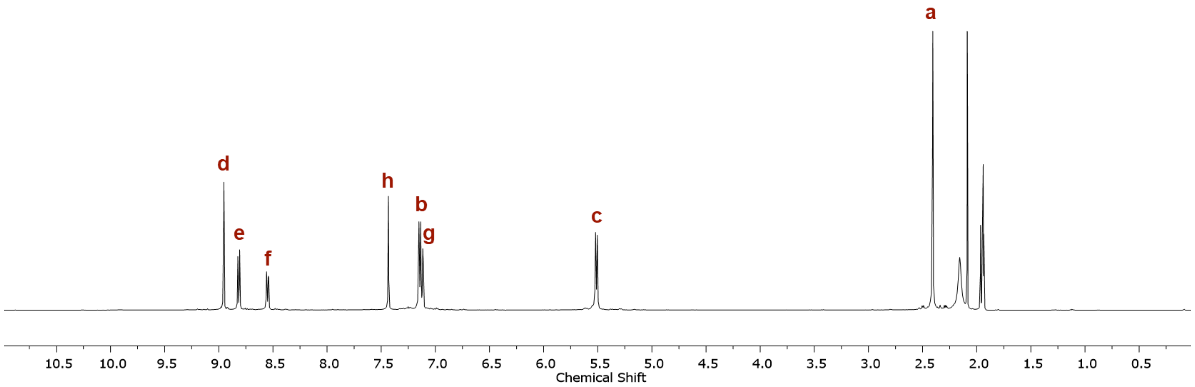

Figure S6: ^{1}H NMR spectrum (500 MHz, 298 K, CD_3CN) of **1**.

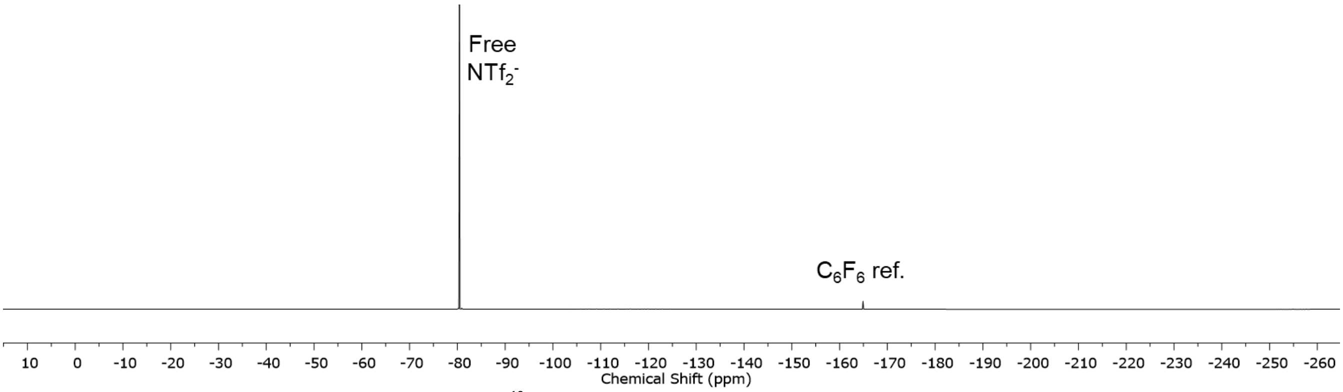

Figure S7: ^{19}F NMR spectrum (471 MHz, 298 K, CD_3CN) of **1**.

The data was consistent with the previously reported data for cage **1** with OTf^- counterions.[3]

2.3 Cage 2

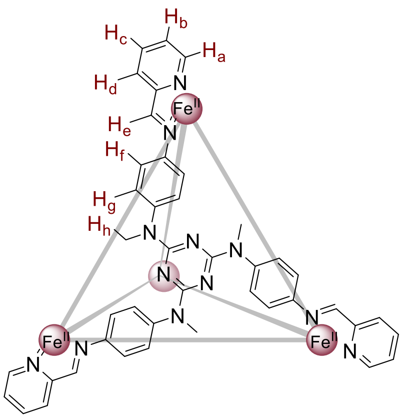

*N*2,*N*4,*N*6-tris(4-aminophenyl)-*N*2,*N*4,*N*6-trimethyl-1,3,5-triazine-2,4,6-triamine **B** (50.0 mg, 0.1133 mmol, 1.0 equiv.) and 2-picolinaldehyde (32.3 µL, 0.3399 mmol, 3.0 equiv.) were dissolved in 10.0 mL of CH_3CN in a sealed 25 mL round bottom flask. The solution was degassed with N_2 for 10 min after which $Fe(NTf_2)_2$ (78.0 mg, 0.1133 mmol, 1.0 equiv.) was added. The solution was degassed for an additional 10 min. The solution was stirred at room temperature for 18 h. The dark purple solution was then concentrated under a flow of nitrogen. Addition of 80 mL of diethyl ether precipitated the compound into a dark blue solid. The solid was separated by centrifugation and washed with diethyl ether (2 × 80 mL). The solid was then dried under a flow of nitrogen.

^{1}H NMR (500 MHz, acetonitrile-d_3) δ 8.84 (s, 12H, **2**-H_e), 8.80 (s, 1H, NTf_2^-⊂**2**-H_e), 8.50 (d, *J* = 7.7 Hz, 12H, **2**-H_d), 8.47 (d, *J* = 7.7 Hz, 1H, NTf_2^-⊂**2**-H_d) 8.39 (t, *J* = 7.7 Hz, 13H, NTf_2^-⊂**2** and **2**-H_c), 7.75 (t, *J* = 7.7 Hz, 13H, NTf_2^-⊂**2** and **2**-H_b), 7.43 – 7.35 (bm, 39H, NTf_2^-⊂**2** and **2**-H_a and H_g), 5.24 – 4.89 (bm, 26H, NTf_2^-⊂**2** and **2**-H_f), 3.41 (s, 39H, NTf_2^-⊂**2** and **2**-H_h).
^{19}F NMR (471 MHz, acetonitrile-d_3) δ -75.40 (NTf_2^-⊂**2**), -80.47 (free NTf_2^-).
LR-ESI-MS [charge, calculated mass]: *m/z* = 1486.4 [**2**$(NTf_2)_5^{3+}$, 1486.5], 1044.2 [**2**$(NTf_2)_4^{4+}$, 1044.8], 779.7 [**2**$(NTf_2)_3^{5+}$, 779.8], 603.1 [**2**$(NTf_2)_2^{6+}$, 603.2], 476.9 [**2**$(NTf_2)^{7+}$, 477.0], 382.2 [$\mathbf{2}^{8+}$, 382.3].

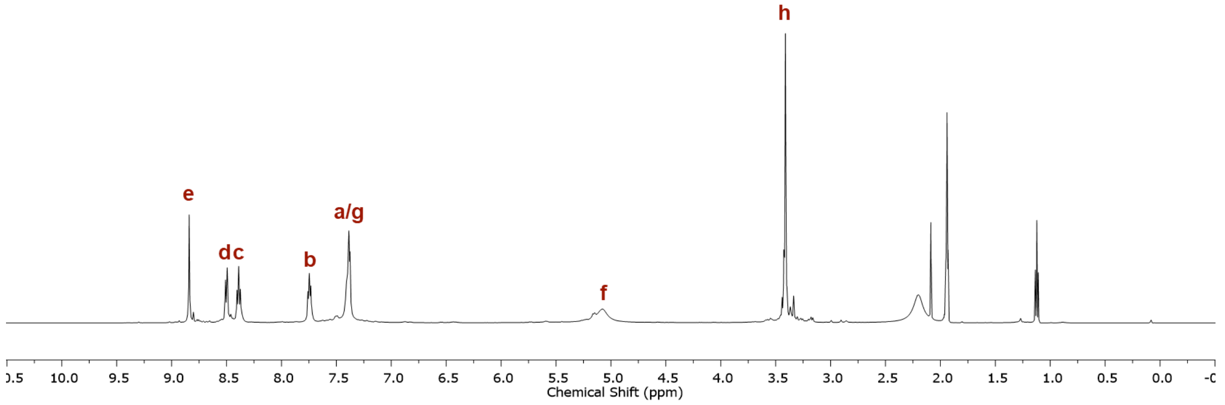

Figure S8: ^{1}H NMR spectrum (500 MHz, 298 K, CD_3CN) of **2**.

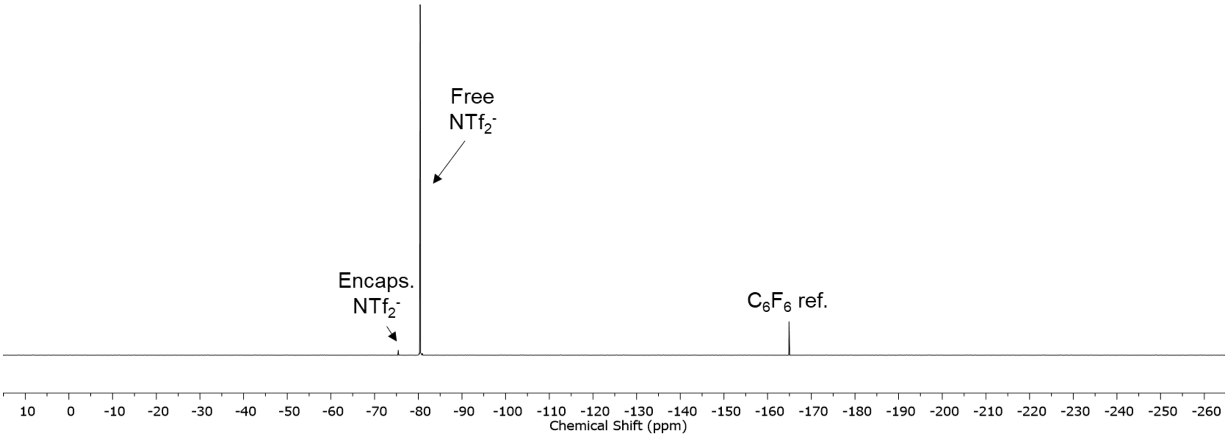

Figure S9: ^{19}F NMR spectrum (471 MHz, 298 K, CD_3CN) of **2**.

The data was consistent with the previously reported data for cage **2** with OTf⁻ counterions.[1c]

2.4) Subcomponent (*p*-aminobenzoyl)-L-Phe-OMe C and cage 3

2.4.1) Subcomponent (*p*-aminobenzoyl)-L-Phe-OMe **C**

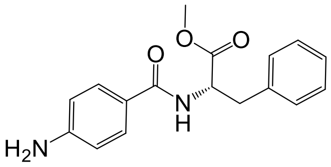

Methyl L-Phenylalaninate.HCl (431.3 mg, 2.00 mmol, 1.2 eq.), 4-nitrobenzoic acid (279 mg, 1.67 mmol, 1.0 eq.), EDC.HCl (0.627 mg, 3.27 mmol, 2 eq.), DIPEA (0.886 ml, 5.10 mmol, 3 eq.) and HOAt (23 mg, 0.17 mmol, 0.1 eq) were combined in THF (50 ml). The mixture were stirred at room temperature for 18 h. The solvents were removed under reduced pressure and the resulting materials was dissolved in 50 ml of CH_2Cl_2. The solution was washed with water (2 × 50 ml), saturated aqueous NH_4Cl (2 × 50 ml) and saturated aqueous $NaHCO_3$ (2 × 50 ml). The organic phase was dried over $MgSO_4$ and the solvents removed under reduced pressure. The resulting solid was purified by silica gel chromatography (CH_2Cl_2) giving the desired nitrophenyl compound as a yellow solid. The nitrophenyl compound obtained was dissolved in CH_3OH and Pd/C (50 mg) was added. The mixture was stirred under H_2 atmosphere for 3 h. The Pd/C was removed by filtration on celite and the solvents removed under reduced pressure to give the desired aniline as an off-white solid (443.9 mg, 1.48 mmol, 89% over two steps).

^{1}H NMR (400 MHz, Chloroform-*d*) δ 7.56 (d, *J* = 8.6 Hz, 2H), 7.32 – 7.20 (m, 3H), 7.17 – 7.04 (m, 2H), 6.64 (d, *J* = 8.6 Hz, 2H), 6.40 (bd, *J* = 7.7 Hz, 1H), 5.14 – 4.93 (m, 1H), 3.95 (bs, 1H), 3.74 (s, 3H), 3.36 – 3.13 (m, 2H).

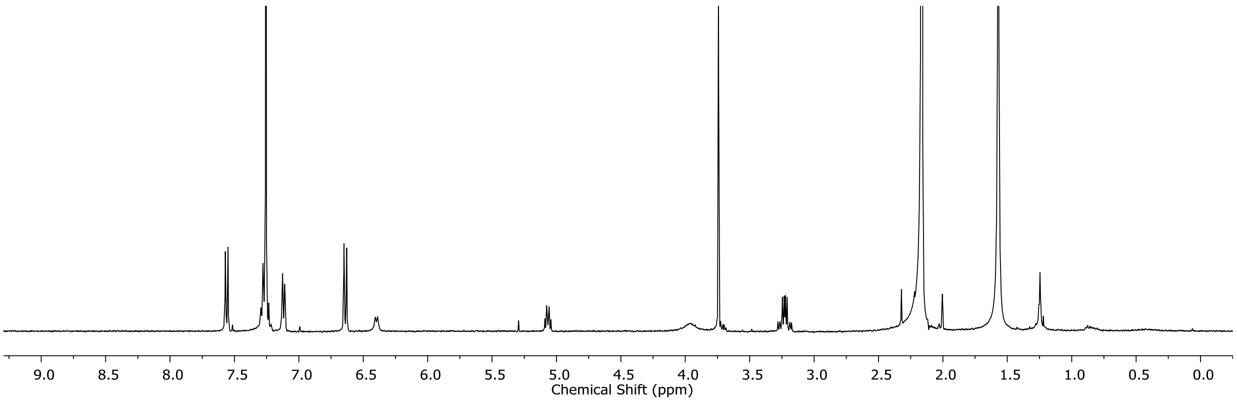

Figure S10: ^{1}H NMR (400 MHz, Chloroform-*d*) of **C**.

These data were consistent with previously reported data.[4]

2.4.2) Cage **3**

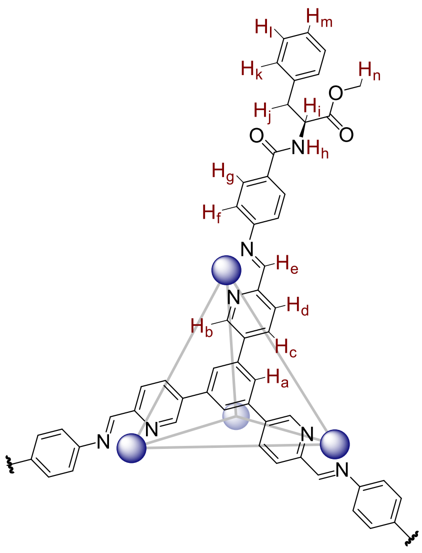

5,5',5''-(benzene-1,3,5-triyl)tripicolinaldehyde **A** (3.6 mg, 0.0092 mmol, 1.0 eq.) and aniline **C** (8.2 mg, 0.0275 mmol, 3.0 eq.) were dissolved in 2.0 mL of CH_3CN in a sealed 5 mL Schlenk flask. Oxygen was removed by freeze-pumped-thawing with N_2 three times after which $Fe(NTf_2)_2$ (6.3 mg, 0.0023 mmol, 1.0 eq.) was added. The solution was freeze-pumped-thawed an additional two times. The solution was heated at 50 °C for 18 h. The dark blue solution was cooled and concentrated under a flow of nitrogen. Addition of 30 mL of diethyl ether precipitated the compound into a dark blue solid. The solid was separated by centrifugation and washed with diethyl ether (2 × 30 mL). The solid was dried under a flow of nitrogen.

^{1}H NMR (400 MHz, Acetonitrile-d_3) δ 9.07 (s, 12H, H_e), 9.04 (s, 12H, H_e), 8.91 (d, *J* = 4.2 Hz, 12H, H_d), 8.89 (d, *J* = 4.3 Hz, 12H, H_d), 8.58 (d, *J* = 8.4 Hz, 24H, H_c), 7.75 – 7.67 (m, 48H, H_g), 7.47 (d, *J* = 2.4 Hz, 24H, H_h), 7.44 – 7.14 (m, 180H, $H_{a,b,k,l,m}$), 5.58 (d, *J* = 8.1 Hz, 48H, H_f), 5.01 – 4.85 (m, 24H, H_i), 3.73 (s, 72H, H_n), 3.41 – 3.10 (m, 48H, H_j). ^{13}C NMR (126 MHz, Acetonitrile-d_3) δ 176.74 (d, *J* = 6.4 Hz), 172.92 (d, *J* = 6.8 Hz),

166.35 (d, J = 4.7 Hz), 159.43, 153.29 (d, J = 7.1 Hz), 152.83, 138.97, 138.13 (d, J = 1.6 Hz), 137.43, 136.84, 135.42 (d, J = 14.9 Hz), 133.82, 130.25 (d, J = 2.8 Hz), 129.86 (d, J = 6.8 Hz), 129.49, 127.89 (d, J = 1.9 Hz), 127.04, 122.61, 122.06, 55.43 (d, J = 14.3 Hz), 53.02, 38.02 (d, J = 2.6 Hz).

LR-ESI-MS: [charge, calculated mass]: *m/z* = 1570.3 [**3**$(NTf_2)_4^{4+}$, 1569.9], 1200.2 [**3**$(NTf_2)_3^{5+}$, 1199.9], 953.5 [**3**$(NTf_2)_2^{6+}$, 953.3], 777.2 [**3**$(NTf_2)^{7+}$, 777.1].

HR-ESI-MS: *m/z* calculated for **3**$(NTf_2)_3^{5+}$ = 1200.0794, observed = 1200.0829.

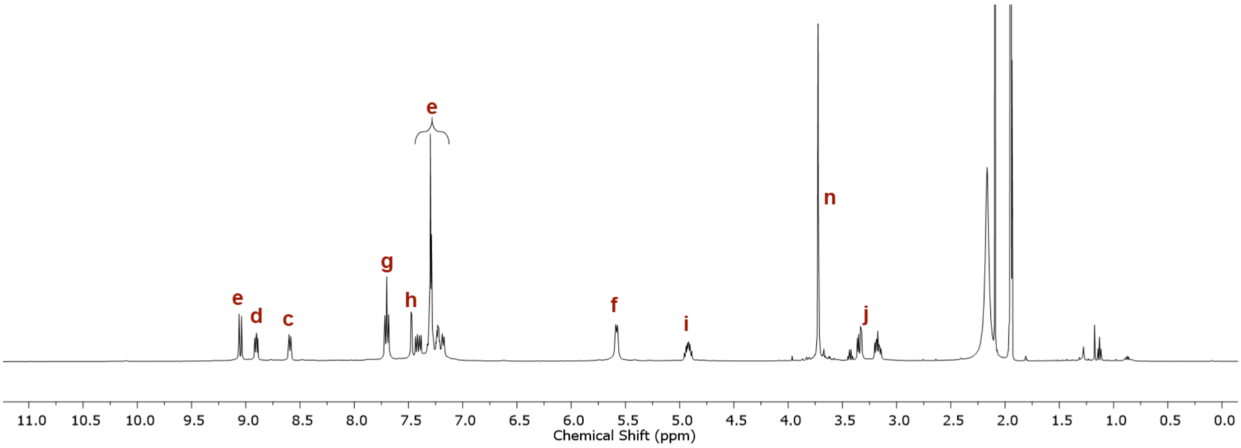

Figure S11: ^{1}H NMR (400 MHz, CD_3CN) of **3.**

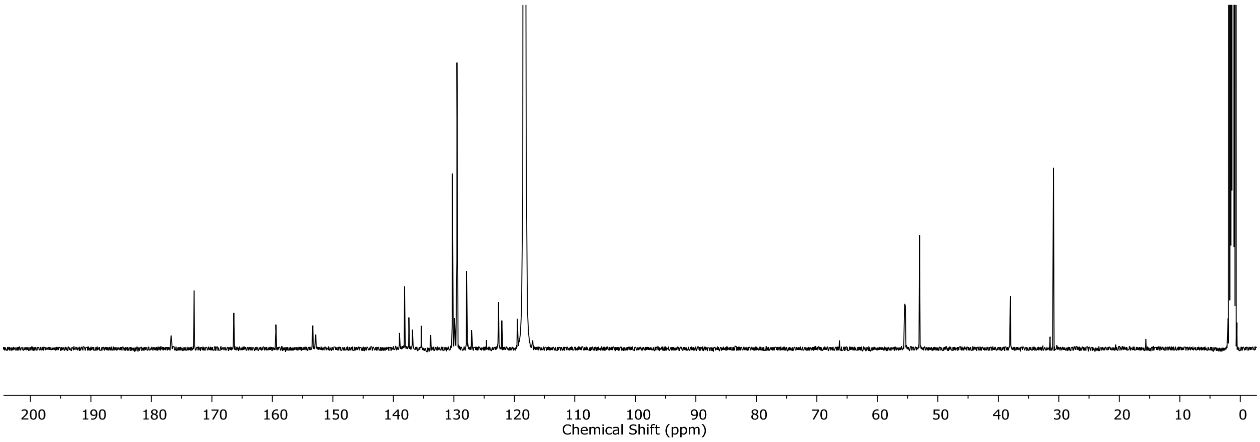

Figure S12: ^{1}H NMR (126 MHz, CD_3CN) of **3.**

3) Host-guest complexes

3.1) TFA$^-$ ⊂ 1

20 Equivalents of K TFA was added to a solution of cage **1** (5 mM) in CD_3CN. The solution was left to equilibrate for 2 h.

^{1}H NMR (400 MHz, acetonitrile-d_3) δ 8.91 (s, 12H, H_d), 8.75 (d, J = 7.8 Hz, 12H, H_e), 8.52 (d, J = 7.2 Hz, 12H, H_f), 7.35 (s, $12H_g$), 7.32 (s, 12H, H_h), 7.10 (d, J = 7.9 Hz, 24H, H_b), 5.50 (d, J = 7.9 Hz, 24H, H_c), 2.38 (s, 36H, H_a).

^{19}F NMR (376 MHz, acetonitrile-d_3) δ -76.00 (bs, free TFA$^-$), -79.76 (s, encaps. TFA$^-$), -80.58 (s, free NTf_2^-).

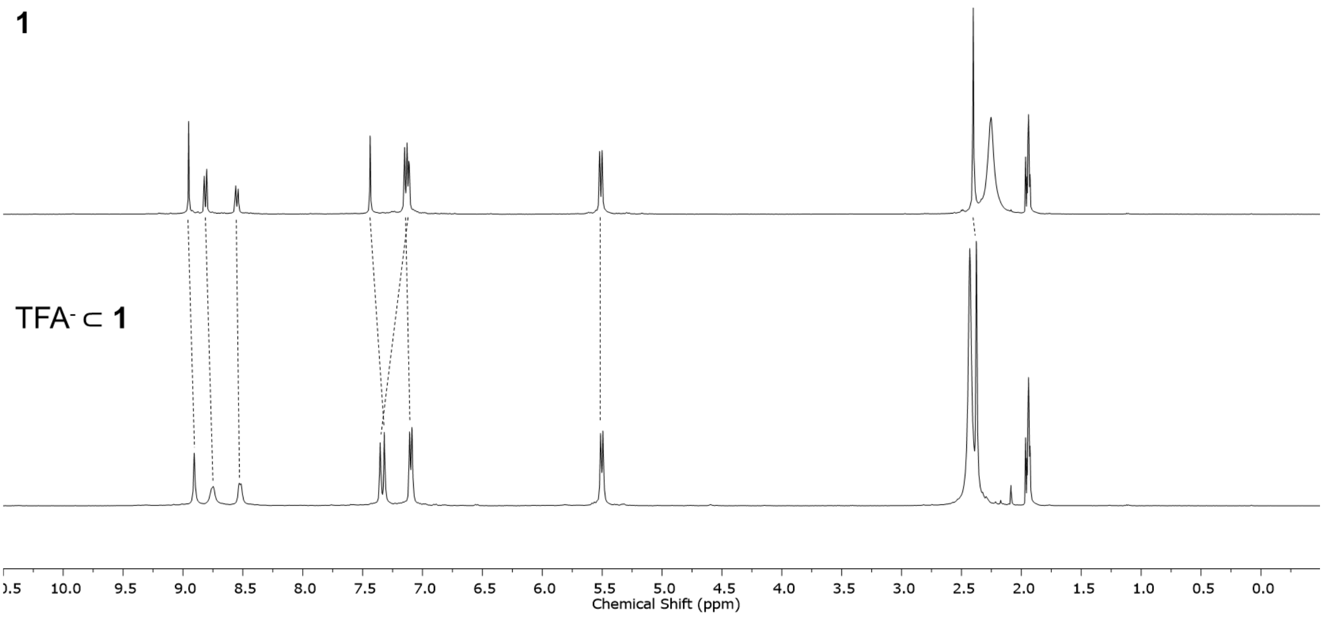

Figure S13: ^{1}H NMR spectrum (400 MHz, 298 K, CD_3CN) of **1** (top) and TFA^- ⊂ **1** (bottom)

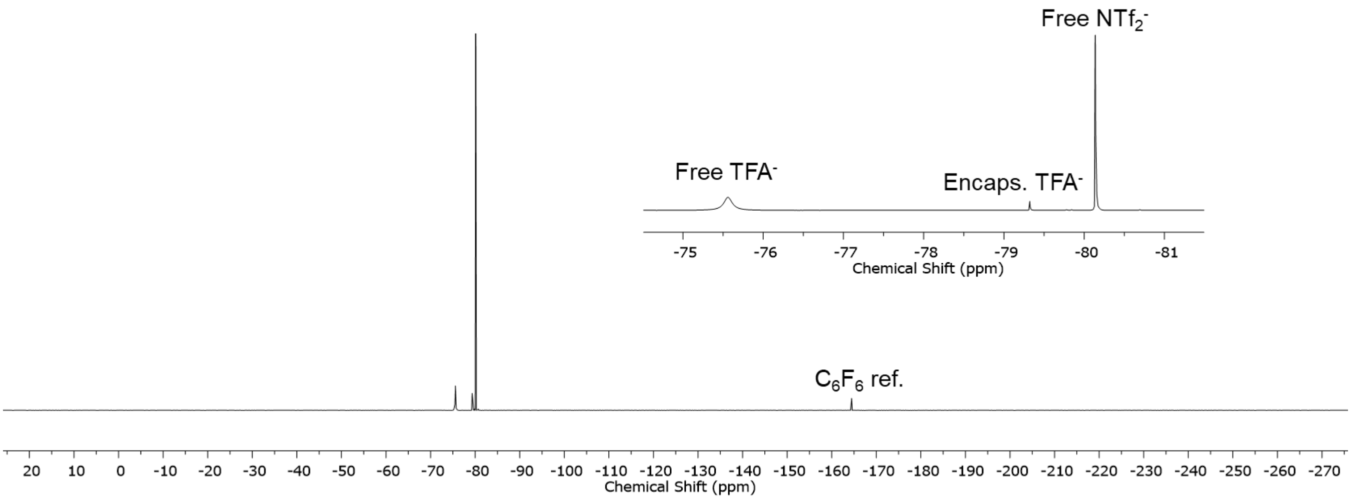

Figure S14: ^{19}F NMR spectrum (376 MHz, 298 K, CD_3CN) of TFA^- ⊂ **1**

3.2) $ReO4^-$ ⊂ 1 in the presence of 20 eq. of TFA^-

One equivalent of TBA ReO_4 was added to a solution of cage **1** (5 mM) containing 20 eq. K TFA in CD_3CN. The solution was left to equilibrate for 4 days. The decrease in intensity of the TFA^- peak in the ^{19}F NMR is due to the competitive encapsulation or ReO_4^-, showing a stronger preference of **1** for ReO_4^- over TFA^-.

^{1}H NMR (400 MHz, acetonitrile-*d*$_3$) δ 8.90 (s, 12H, H_d), 8.76 (bs, 12H, H_e), 8.51 (bs, 12H, H_f), 7.31 (s, 1H, H_g), 7.29 (s, 12H, H_h), 7.10 (d, *J* = 7.8 Hz, 24H, H_b), 5.53 (d, *J* = 8.1 Hz, 24H, H_c), 3.15 – 2.98 (m, 1H, TBA^+), 2.38 (s, 36H, H_a), 1.59 (ddd, *J* = 12.0, 9.9, 6.2 Hz, 8H, TBA^+), 1.35 (h, *J* = 7.4 Hz, 8H, TBA^+), 0.96 (t, *J* = 7.3 Hz, 12H, TBA^+).

^{19}F NMR (376 MHz, acetonitrile-*d*$_3$) δ -76.08 (bs, free TFA^-), -79.76 (s, encaps. TFA^-), -80.58 (s, NTf_2^-).

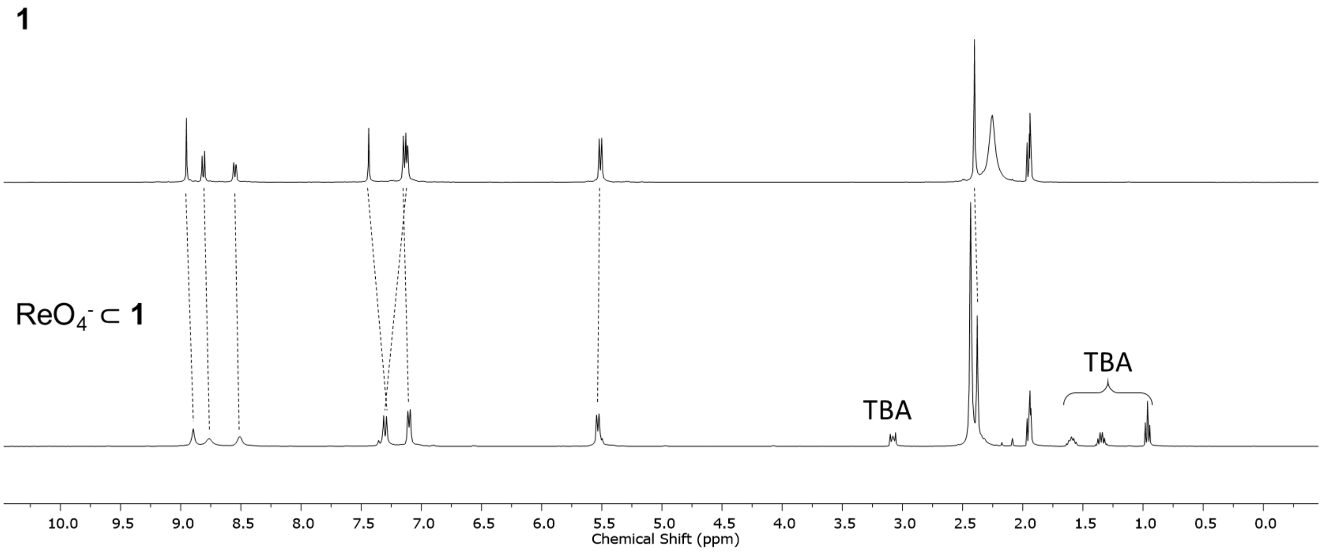

Figure S15: ^{1}H NMR spectrum (400 MHz, 298 K, CD_3CN) of **1** (top) and $ReO_4^- \subset$ **1** in the presence of 20 eq. TFA^-(bottom).

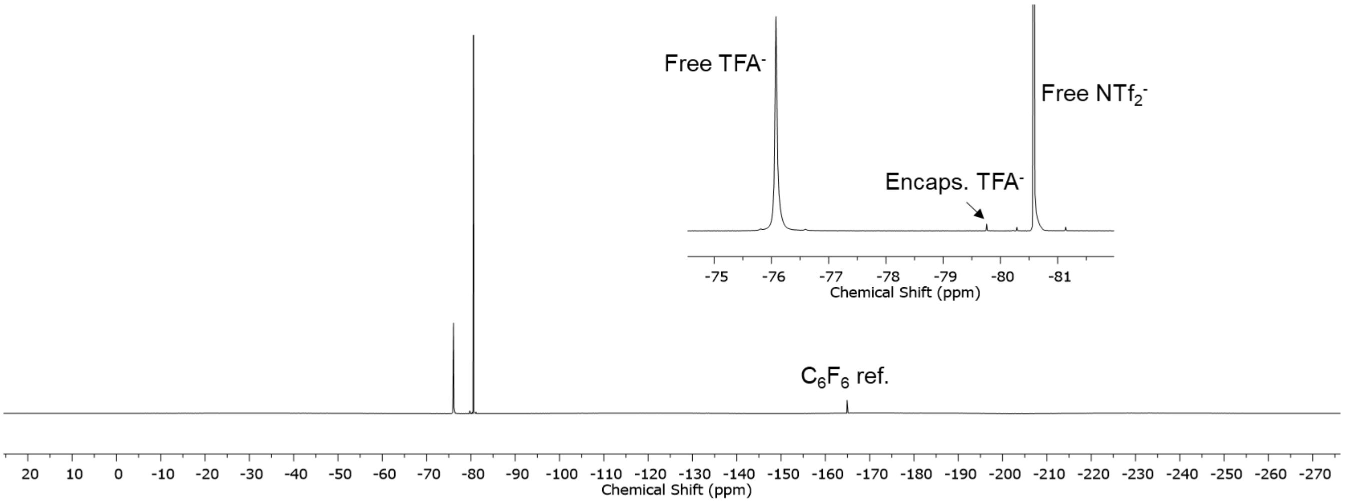

Figure S16: ^{19}F NMR spectrum (376 MHz, 298 K, CD_3CN) of $TFA^- \subset$ **1** in the presence of 20 eq. TFA^-.

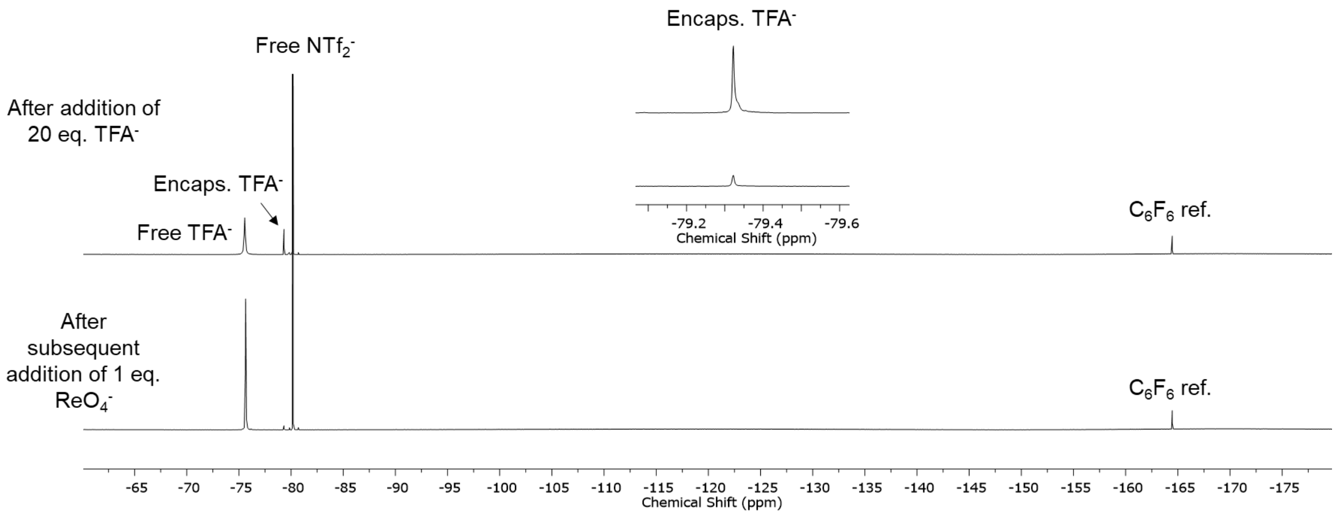

Figure S17: ^{19}F NMR spectrum (376 MHz, 298 K, CD_3CN) of $ReO_4^- \subset$ **1** in the presence of 20 eq. TFA^- (bottom) compared to the spectrum before the addition of ReO_4^- (top) showing the decrease in the intensity of the encapsulated TFA^- peak.

3.3) FA ⊂ 2 (FA = Fluoroadamantane)

*N*2,*N*4,*N*6-tris(4-aminophenyl)-*N*2,*N*4,*N*6-trimethyl-1,3,5-triazine-2,4,6-triamine **B** (24.8 mg, 0.056 mmol, 4.0 equiv.), 2-picolinaldehyde (16.0 µL, 0.168 mmol, 12.0 equiv.) and FA (13.0 mg, 0.084 mmol, 6 equiv.) were dissolved in 13.5 mL of CH_3CN in a sealed 25 mL round bottom flask. The solution was degassed with N_2 for 10 min after which $Fe(NTf_2)_2$ (38.7 mg, 0.1133 mmol, 1.0 equiv.) was added. The solution was degassed for an additional 10 min. The solution was stirred at room temperature for 18 h. The dark purple solution was then concentrated under a flow of nitrogen. Addition of 80 mL of diethyl ether precipitated the compound into a dark blue solid. The solid was separated by centrifugation and washed with diethyl ether (2 × 80 mL). The solid was then dried under a flow of nitrogen.

^1H NMR (400 MHz, acetonitrile-d_3) δ 8.84 (s, 12H, H_e), 8.50 (dd, J = 7.9, 1.4 Hz, 12H, H_d), 8.40 (dd, J = 7.7, 7.8 Hz, 12H, H_c), 7.75 (ddd, J = 7.7, 7.8, 1.5 Hz, 12H, H_b), 7.53 – 7.23 (b, 24H, H_g), 7.38 (d, J = 5.6 Hz, 12H, H_a), 5.90 – 4.11 (b, 24H, H_f), 3.42 (s, 36H, H_h).
^{19}F NMR (376 MHz, acetonitrile-d_3) δ -80.47 (free NTf_2^-), -121.11 (encaps. FA).

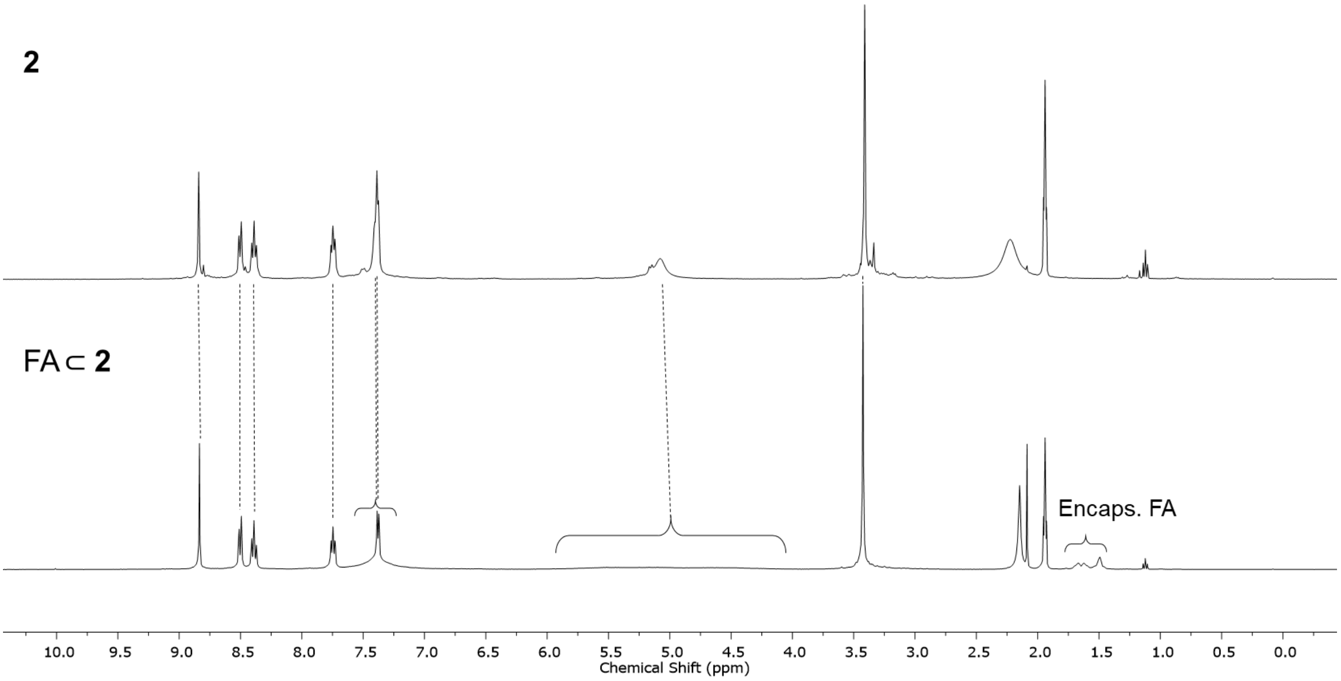

Figure S18: ^1H NMR spectrum (400 MHz, 298 K, CD_3CN) of **2** (top) and FA ⊂ **2** (bottom).

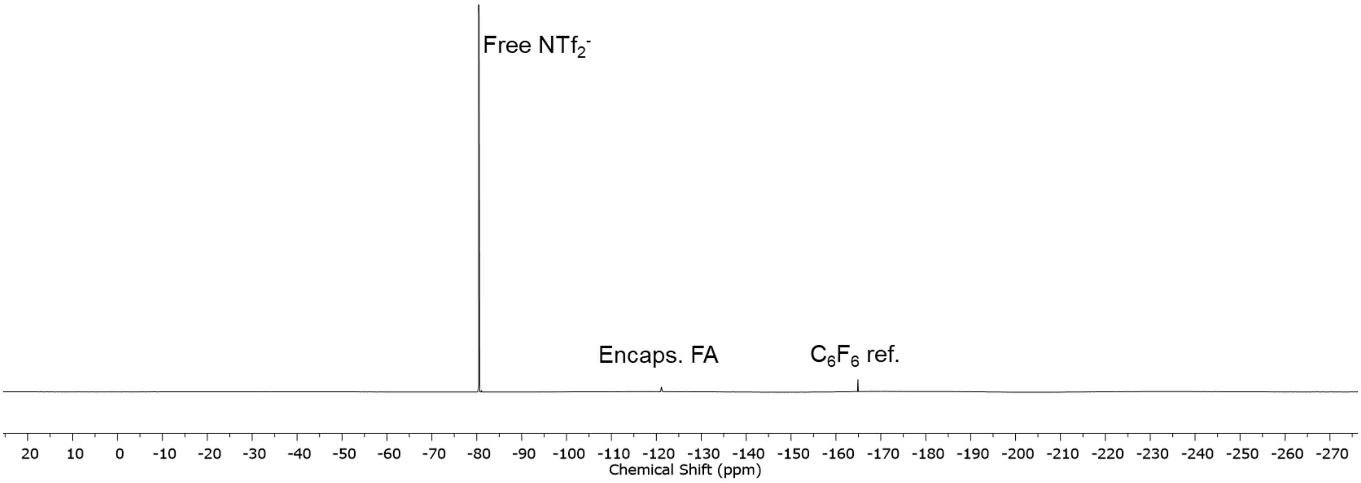

Figure S19: ^{19}F NMR spectrum (376 MHz, 298 K, CD_3CN) of FA ⊂ **2.**

The data was consistent with the previously reported data for cage **2** binding FA.[5]

4) Gels formation and characterization

4.1) General preparation of peptide gel

4 mg of *p*-aminobenzoyl-L-Phe-D-Ala-L-Phe-NH_2 were dissolved in acetonitrile by heating. The sample was left to cool down to room temperature and the gel was formed in 20 minutes.

Table S1. Attempts for the preparation of the peptide gel.

Concentration (mM)	Gel	Time for gelation
10 mM	NO	Solution even after 24 hours
20 mM	NO	Solution even after 24 hours
30 mM	YES	3 hours
40 mM	YES	1 hour 30 minutes
50 mM	YES	20 minutes
50 mM[a]	YES	10 seconds

[a] sonicating in an ultrasonic bath.

Table S2. Attempts for the preparation of the peptide gel in different solvents.

Concentration (mM)	Gel	Gelation	Observations
Acetonitrile	30 mM	YES	Minimum Gelation Concentration (MGC)
Ethanol	50 mM	NO	High solubility
Methanol	50 mM	NO	High solubility
Ethyl acetate	50 mM	NO	Not soluble
	40 mM	NO	Not soluble
	20 mM	NO	Not soluble
	10 mM	NO	Soluble
Chloroform	50 mM	NO	Not soluble
	40 mM	NO	Not soluble
	20 mM	NO	Not soluble
	10 mM	NO	Soluble
Acetonitrile-Ethyl acetate (1:9)	20 mM	YES	Sonication 30 seconds[a]
Acetonitrile-Chloroform (1:9)	20 mM	YES	Sonication 4 minutes[a]

[a]The peptide was dissolved in acetonitrile by heating. Then the other solvent was added to get a final concentration of 20 mM and it was sonicated for the indicated time to get the gel.

4.2) General preparation of peptide/cage gel systems (1⊂Gel and 2⊂Gel)

For the gels containing 1 mM of cage, a stock solution of 5 mM cage was prepared in acetonitrile. Then, the peptide was dissolved in acetonitrile and added to the required volume of the cage solution to get the desired final concentrations of peptide and cage of 50 mM and 1 mM, respectively. The sample was ultrasonicated for 5 minutes and the gel was formed. For the gels containing 5 mM of cage, a stock solution of 20 mM cage was prepared in acetonitrile. Then, the peptide was dissolved in acetonitrile and added to the required volume of the cage solution to get the desired final concentrations of peptide and cage of 50 mM and 5 mM, respectively. The sample was ultrasonicated for 10 minutes and the gel was formed.

4.3) Circular Dichroism.

The analysis of the samples at the concentration of peptide required for gelation (50 mM) was not viable since the voltage goes above ~ 600 and the detector is saturated (signal too noisy to be reliable). Thus, we selected 10 mM as peptide concentration and

different concentrations of cage (0; 0.05; 0.1; 0.15 and 0.2 mM). Samples were freshly prepared, and the spectra immediately recorded.

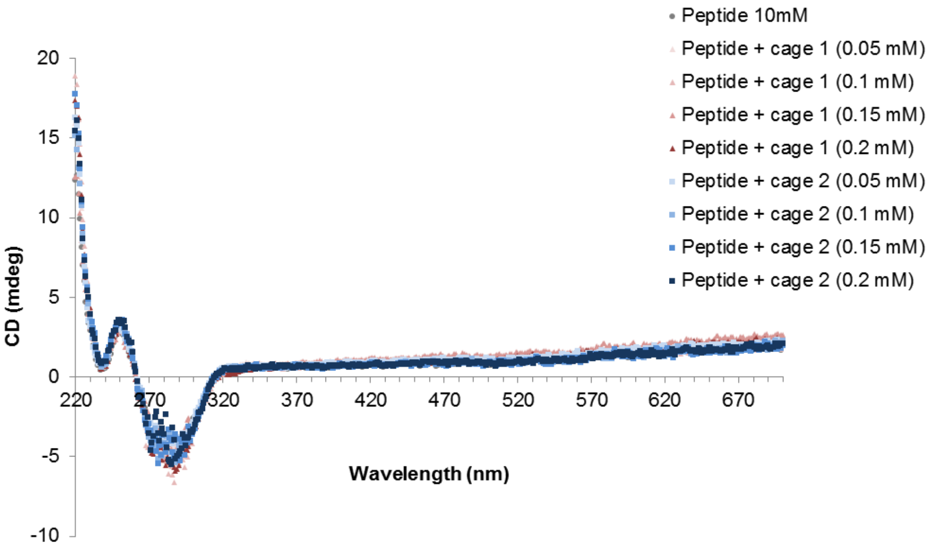

Figure S20: CD spectra of peptide and peptide+cage **1** or **2** at different concentrations.

4.4) Oscillatory rheometry.

Table S3. Elastic (G') and viscous (G'') moduli for the different gels described here. The values shown in the table are calculated as the average (standard deviation included) of 3 independent experiments performed using 3 different samples after 1 hour of self-assembly. The peptide concentration was kept at 50 mM for the different hybrid gels.

Sample	G' (KPa)	G'' (KPa)
Peptide Gel (50 mM)	46.4 ± 4.0	1.8 ± 0.7
1⊂Gel ([MOC 1] = 1 mM)	18.4 ± 1.4	0.7 ± 0.3
2⊂Gel ([MOC 2] = 1 mM)	18.1 ± 0.5	0.9 ± 0.1
1⊂Gel ([MOC 1] = 5 mM)	12.3 ± 0.3	0.2 ± 0.1
2⊂Gel ([MOC 2] = 5 mM)	6.1 ±1.7	0.3 ± 0.1

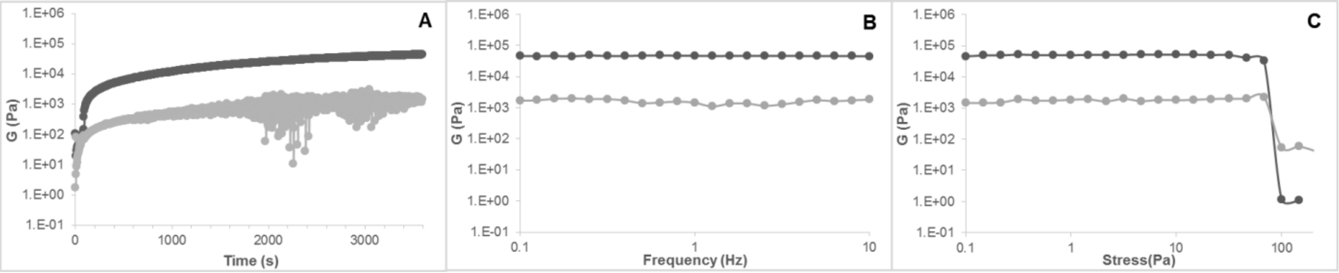

Figure S21: Oscillatory rheometry analysis of peptide gel (50 mM) time sweeps (**A**); frequency sweep (**B**) and stress sweep (**C**), with the elastic modulus (G') in dark grey and the viscous modulus (G'') in light grey.

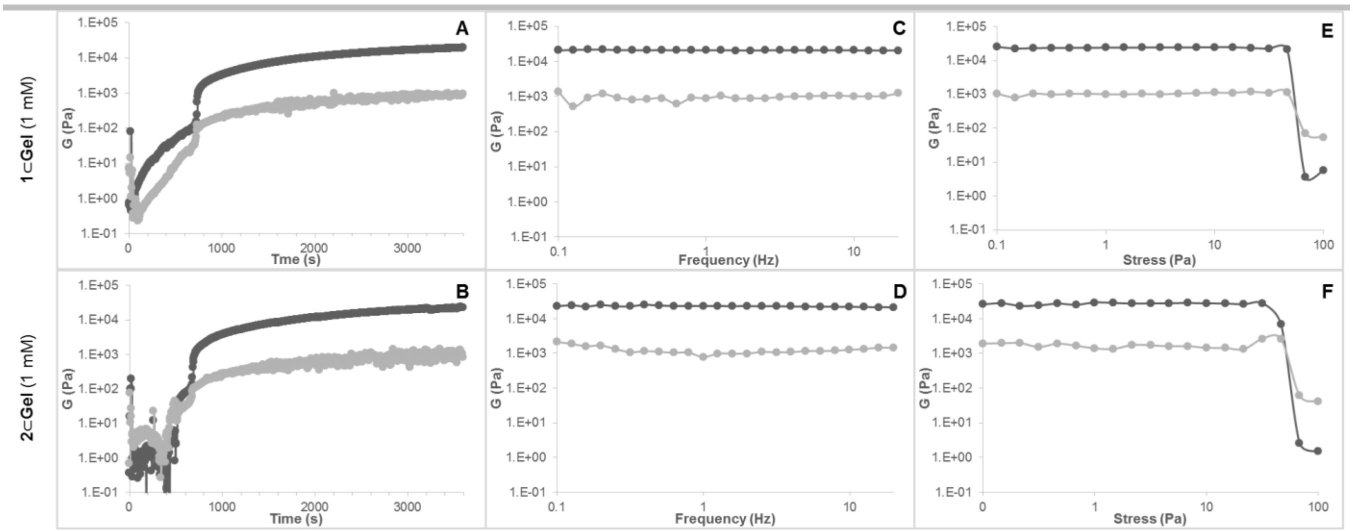

Figure S22: Oscillatory rheometry analysis of **1⊂Gel** and **2⊂Gel** (1 mM of MOC and 50 mM of peptide) time sweeps (**A**-**B**); frequency sweep (**C**-**D**) and stress sweep (**E**-**F**), with the elastic modulus (G') in dark grey and the viscous modulus (G'') in light grey.

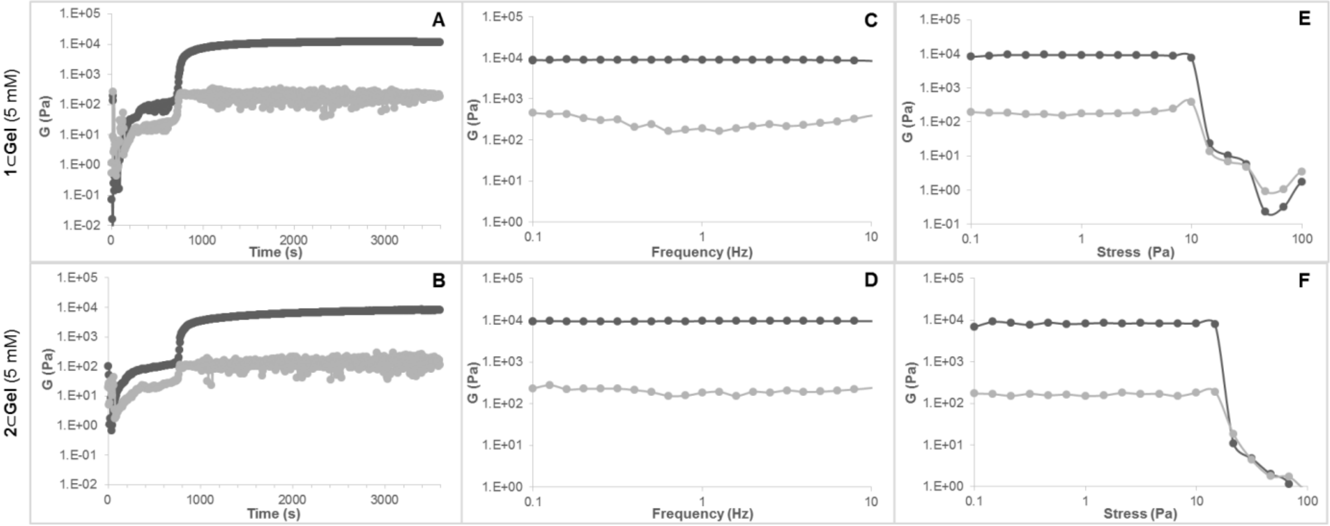

Figure S23: Oscillatory rheometry analysis of **1⊂Gel** and **2⊂Gel** (5 mM of MOC and 50 mM of peptide) time sweeps (**A**-**B**); frequency sweep (**C**-**D**) and stress sweep (**E**-**F**) , with the elastic modulus (G') in dark grey and the viscous modulus (G'') in light grey.

4.5) UV analysis.

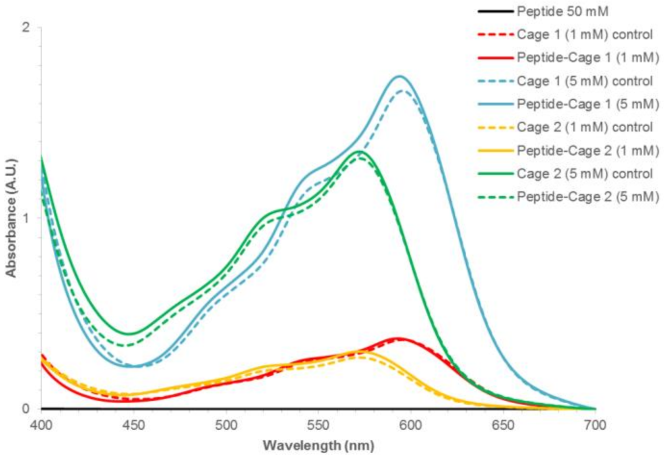

Figure S24: UV-visible spectrum for the gels of peptide at 50 mM and the indicated concentrations of cage.

UV-visible analyses of both MOCs **1** and **2** in solution displayed a characteristic band in the visible region (400-700 nm), attributed to the metal-to-ligand charge transfer (MLCT). This absorbance was conserved in **1⊂Gel**, and **2⊂Gel**, providing further evidence of MOC stability.

4.6) Transmission electron microscopy.

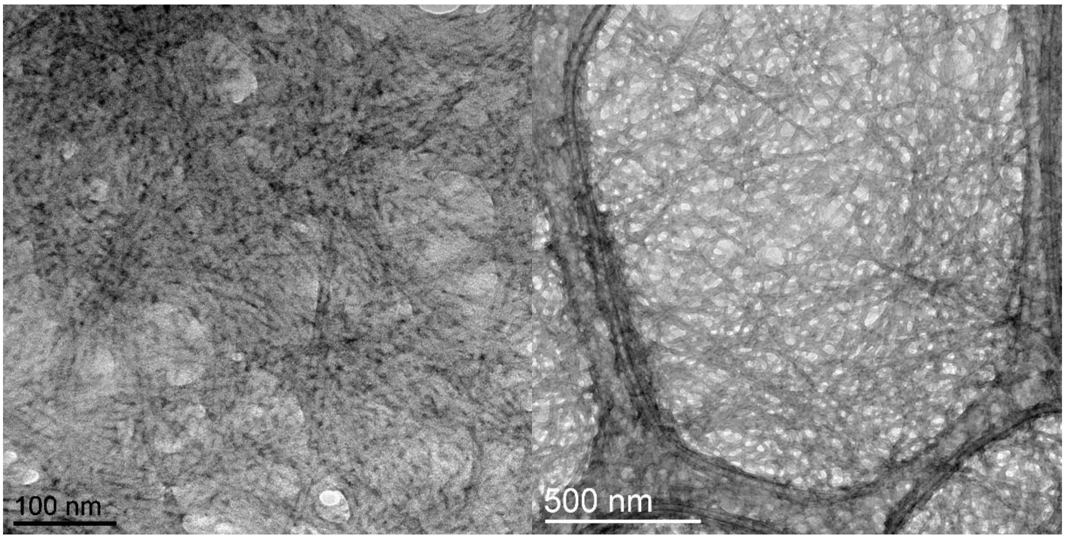

Figure S25: TEM micrographs for the peptide gel (50 mM) at high (left) and low (right) magnification.

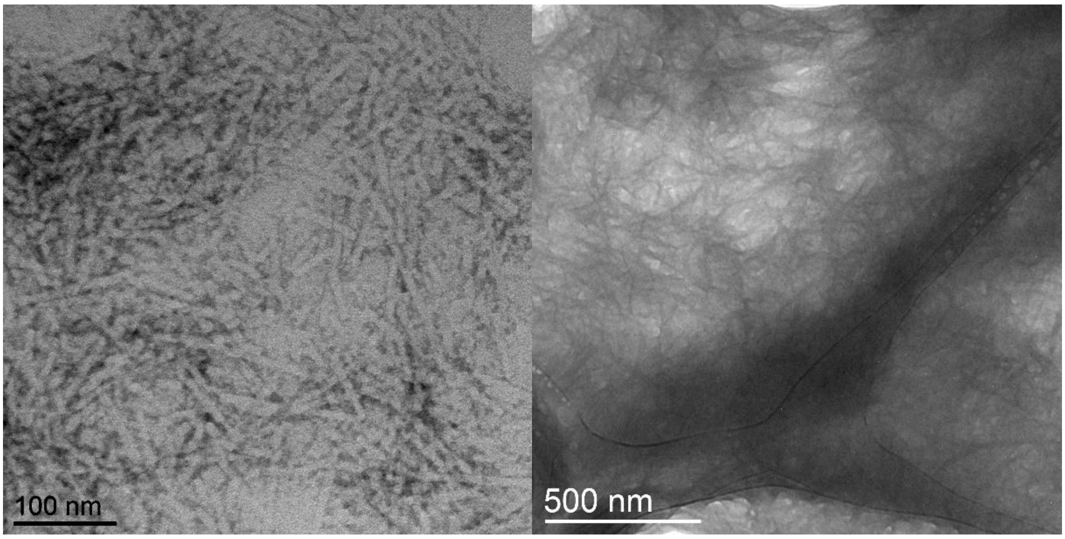

Figure S26: TEM micrographs for the **1⊂Gel** (1 mM) at high (left) and low (right) magnification.

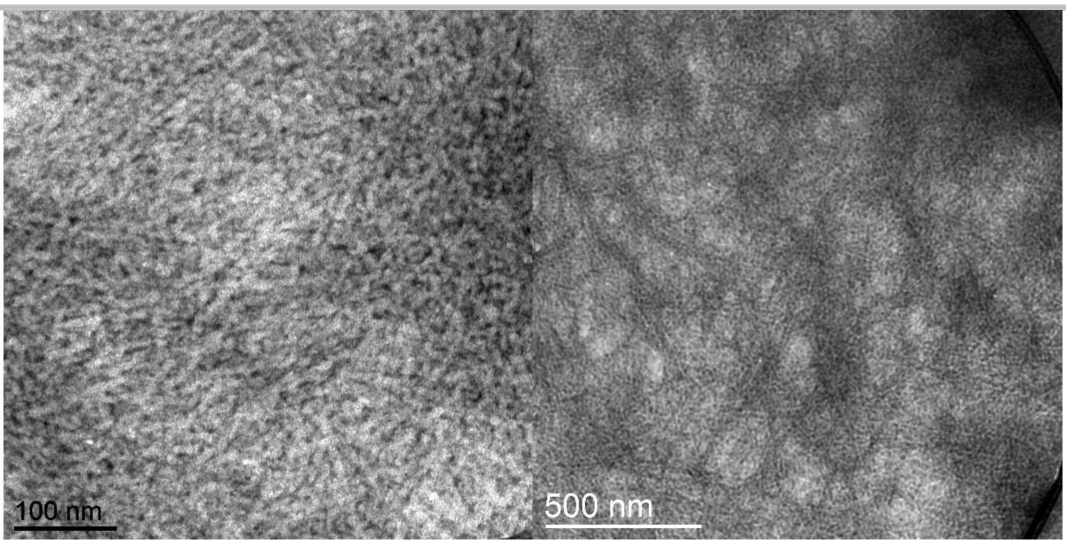

Figure S27: TEM micrographs for the **2⊂Gel** (1 mM) at high (left) and low (right) magnification.

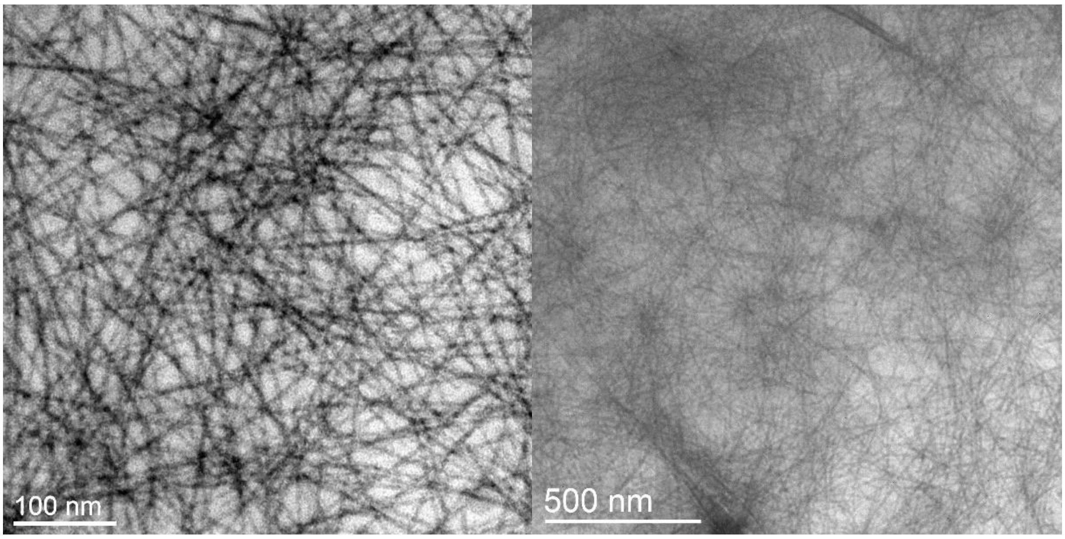

Figure S28: TEM micrographs for the **1⊂Gel** (5 mM) at high (left) and low (right) magnification.

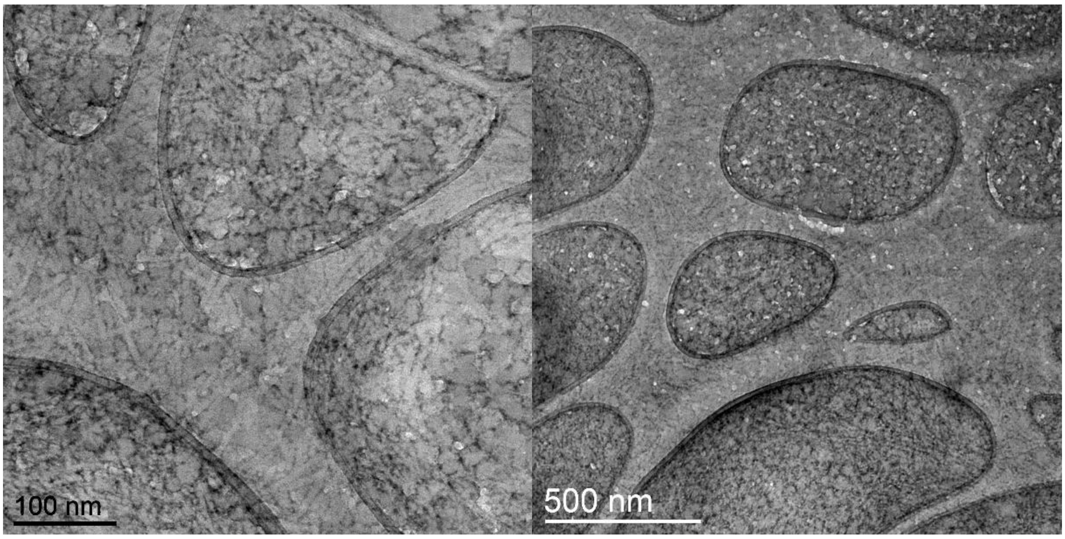

Figure S29: TEM micrographs for the **2⊂Gel** (5 mM) at high (left) and low (right) magnification.

Table S4. Average mass ratio among the K, Fe, Cu, and W elements determined on >15 different spots per sample by EDXS analysis. Elements such as C, N, and O were not taken into account because EDXS analysis is not very suitable for quantification of these light elements. Thus, the obtained data should be assumed as "semi-quantitative". Cu is present in the copper TEM grid; K and W are present in the potassium phosphotungstate stain; Fe is present in both cages.

Element	keV	**Peptide gel 50 mM**	**1⊂Gel (1 mM)**	**2⊂Gel (1 mM)**	**1⊂Gel (5 mM)**	**2⊂Gel (5 mM)**
		Mass % ± SD	*Mass % ± SD*	*Mass % ± SD*	*Mass % ± SD*	*Mass % ± SD*
K – K shell	3.312	2.9 ± 0.7	2.6 ± 0.9	2.6 ± 1.1	4.3 ± 1.9	2.4 ± 1.3
**Fe – K shell**	**6.398**	**0.3 ± 0.1**	**1.9 ± 0.4**	**0.9 ± 0.6**	**5.9 ± 2.1**	**3.2 ± 0.4**
Cu – K shell	8.040	63.0 ± 4.1	65.0 ± 6.1	64.5 ± 7.2	51.2 ± 4.3	72.5 ± 5.3
W – K shell	1.774	33.8 ± 3.1	30.5 ± 2.9	32.0 ± 5.6	38.6 ± 4.9	21.9 ± 6.0

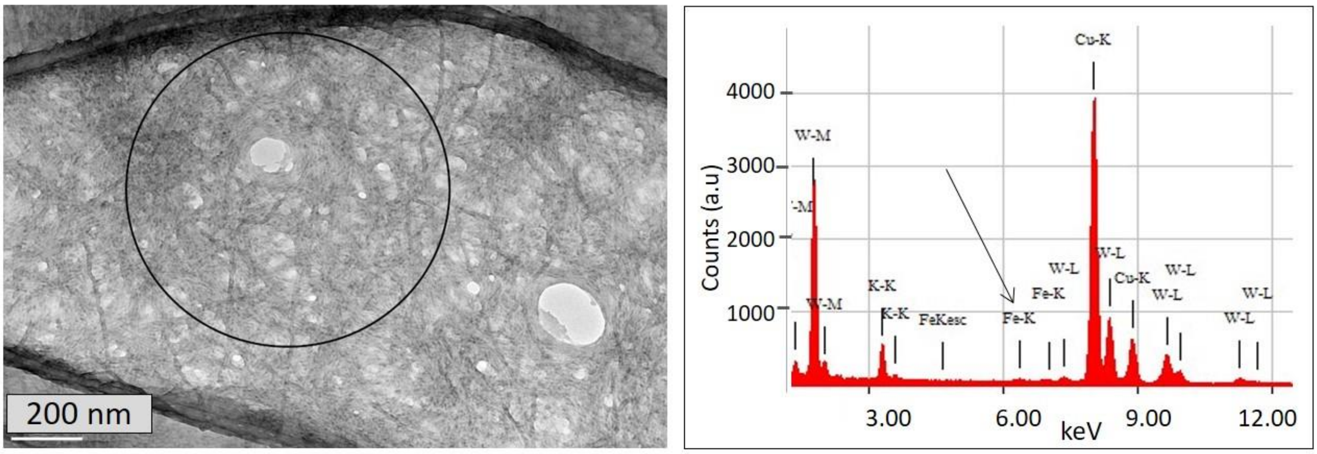

Figure S30: TEM micrograph for the peptide C gel (50 mM) marked with a circle (left) where EDXS was performed and the corresponding EDXS spectrum (right).

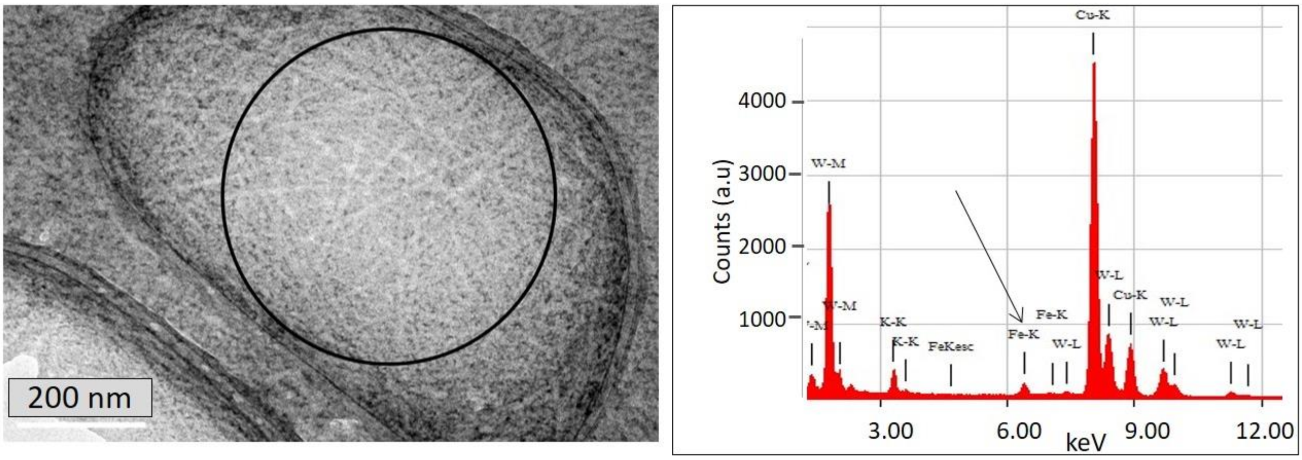

Figure S31: TEM micrograph for the **1⊂Gel** (1 mM) marked with a circle (left) where EDXS was performed and the corresponding EDXS spectrum (right).

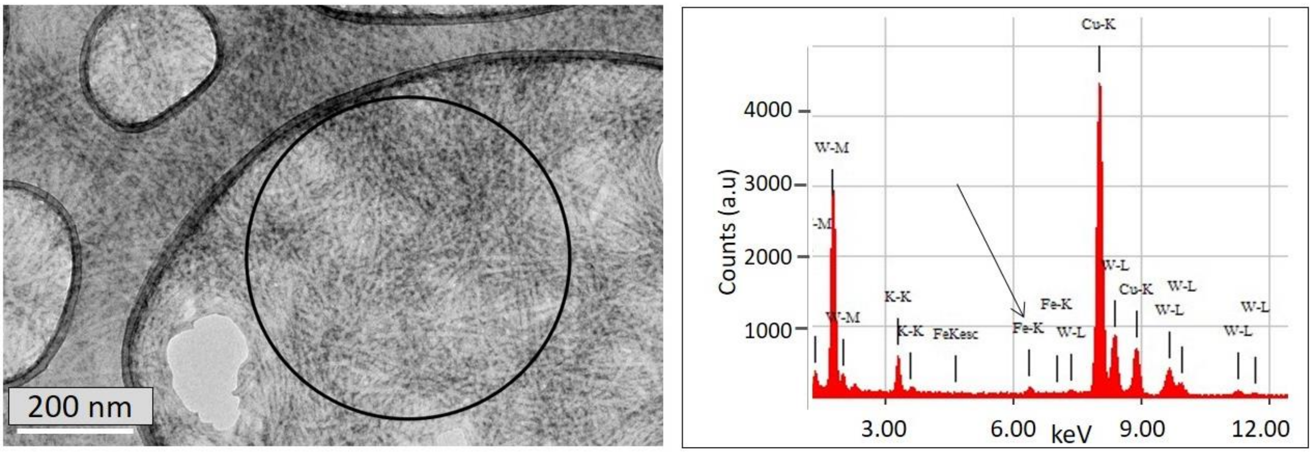

Figure S32: TEM micrograph for the **2⊂Gel** (1 mM) marked with a circle (left) where EDXS was performed and the corresponding EDXS spectrum (right).

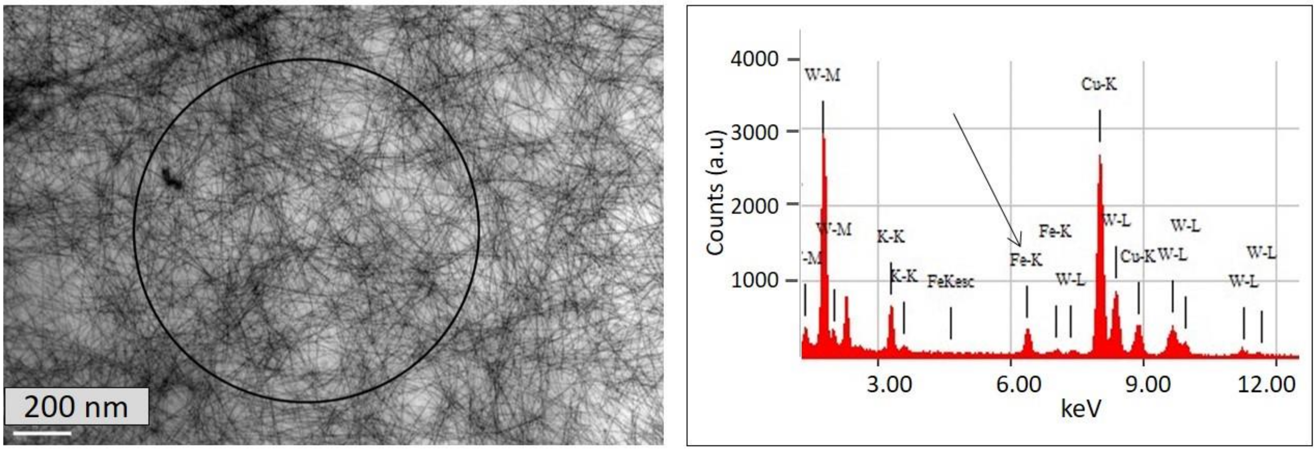

Figure S33: TEM micrograph for the **1⊂Gel** (5 mM) marked with a circle (left) where EDXS was performed and the corresponding EDXS spectrum (right).

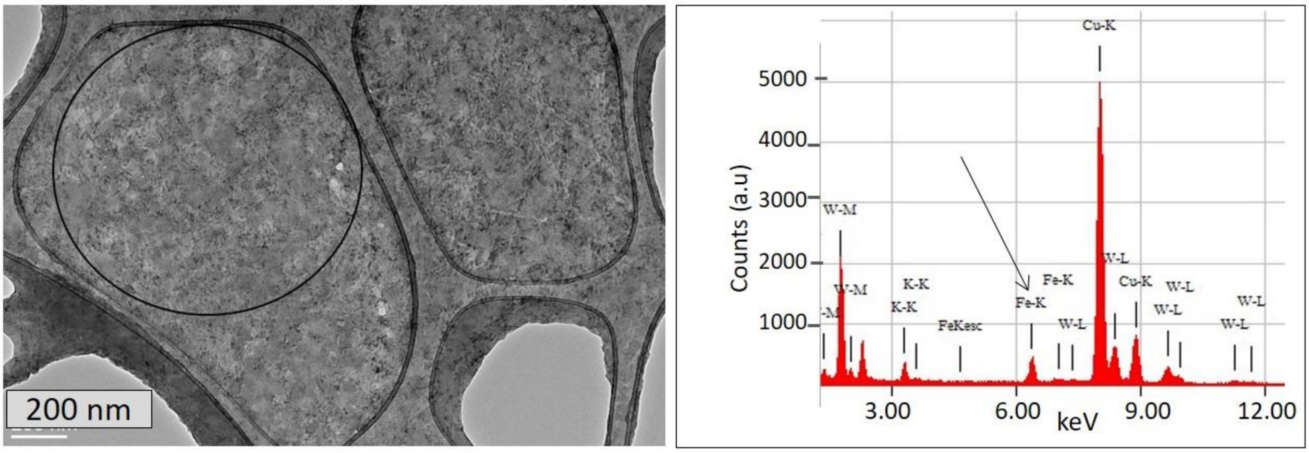

Figure S34: TEM micrograph for the **2⊂Gel** (5 mM) marked with a circle (left) where EDXS was performed and the corresponding EDXS spectrum (right).

4.7) Raman spectroscopy.

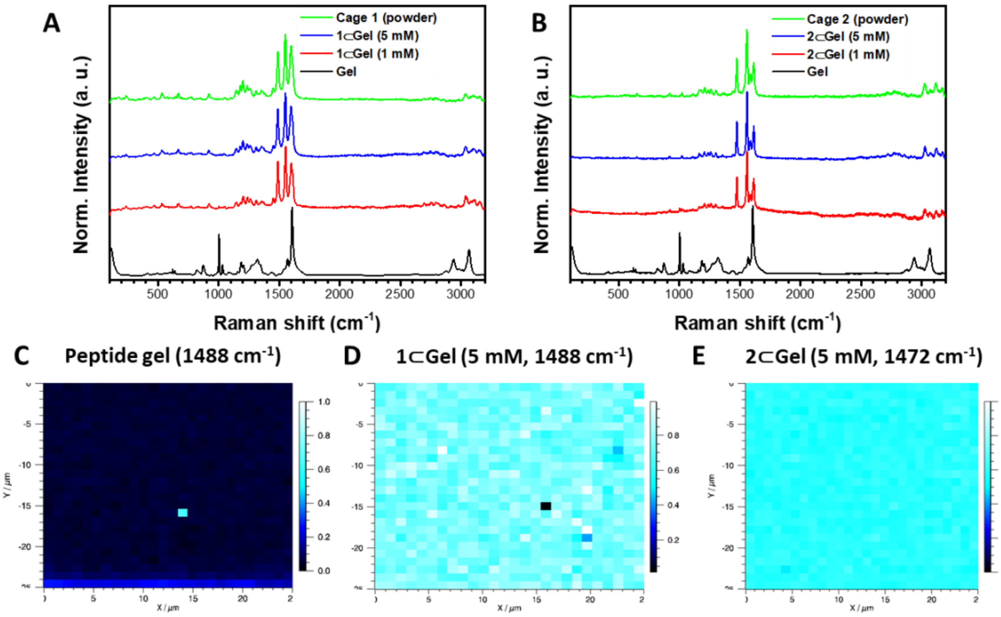

Figure S35: Raman characterization including Raman spectra of the gels with or without cages and cages in the powder form (A and B); mapping of gels without cages (C), with cage **1** (D) and with cage **2** (E).

This technique is quick and non-destructive, and has also been fruitfully applied to other peptide gel composite materials.[6] In the present case, the native gel displayed intense signals at 1004, 1037, 1570, and 1607 cm^{-1}. To our knowledge, imine-based supramolecular capsules have not previously been studied by Raman. Spectra of both MOCs displayed characteristic, intense bands between 1450 and 1650 cm^{-1}, which were attributed to C=N and N-Fe stretching, and to the Raman-active modes of the ligand. Raman signals attributed to the MOCs dominated the spectra of **1⊂Gel** and **2⊂Gel**, confirming the integrity of the cages.

5) Host-guest chemistry in gels

All samples were prepared in sealed J-Young NMR tubes to prevent evaporation of the solvent over time. Samples were monitored by ^{1}H and ^{19}F NMR for a period of 3 weeks.
The errors on the values of the integrals (reading error) were assumed to be 5%.

5.1) Stability of cages 1 and 2

*5.1.1) Stability of **1** and **2** in solution*

To 0.35 mL of a 5 mM solution of cage **1** or FA ⊂ **2** in CD_3CN was added 20 equivalents (35 µmol) of KTFA dissolved in 20 µL of CD_3CN. This aims at replicating the amount of TFA^- counter-ions present in solution due to the peptide. The encapsulated guest peak in the ^{19}F NMR spectra was monitored over time and integrated against the triflimide peak. The values were normalised so that the highest point (at 24 h for **1** and 12 h for **2**) is equal to 100% as only the host-guest complex was observed at this stage.

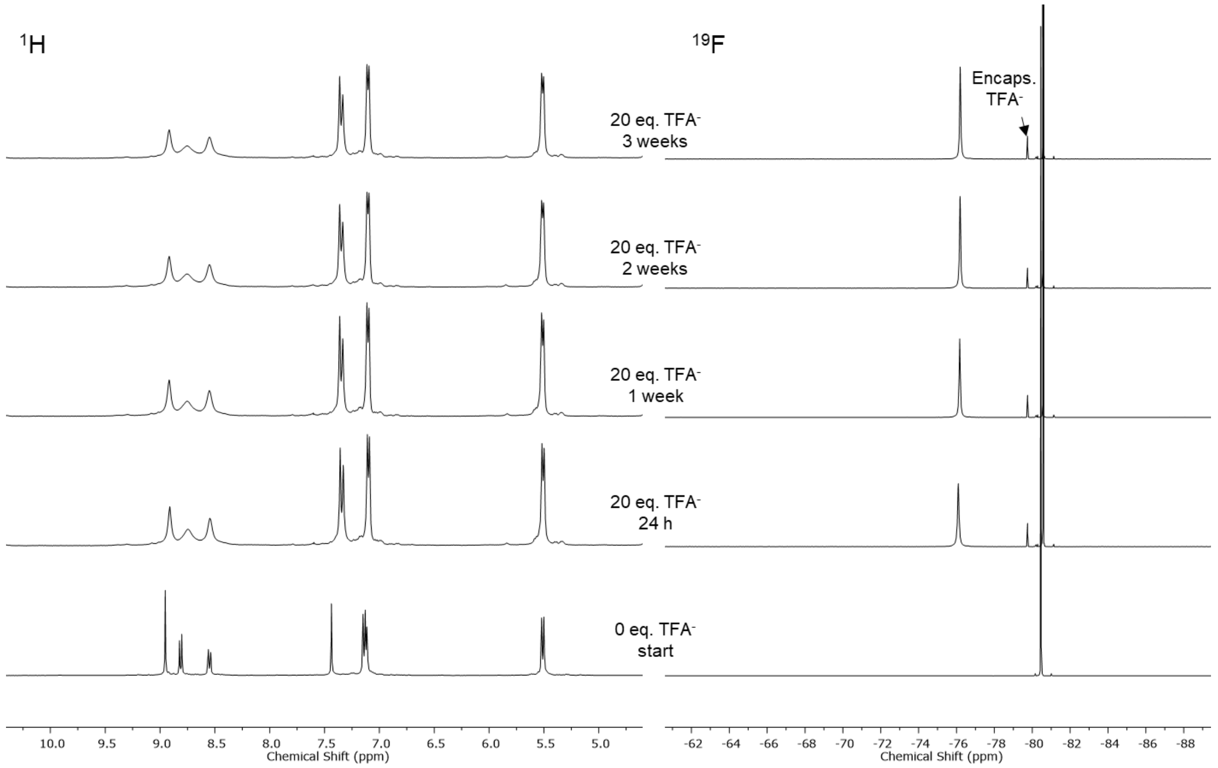

Figure S36: ^{1}H NMR spectra (400 MHz, 298 K, CD_3CN) (left) and ^{19}F NMR spectra (376 MHz, 298 K, CD_3CN) (right) of TFA^- ⊂ **1** over three weeks after addition of 20 eq. of K TFA.

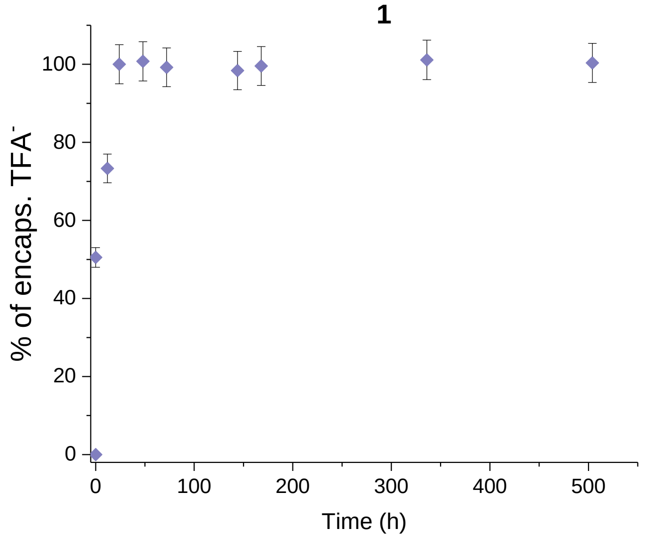

Figure S37: Amount of encapsulated TFA^- in **1** in solution as a function of time.

No precipitate was observed to form over time and no significant drop in the intensity of the encapsulated TFA^- peak in the NMR was observed, demonstrating the stability of the cage in solution in the presence of 20 eq. of KTFA. The small shift of the NTf_2^- peak observed upon addition of KTFA was attributed to the change in the environment of the cage due to TFA^- potentially associating with the outside of the cage and due to small change in the solution's acidity.

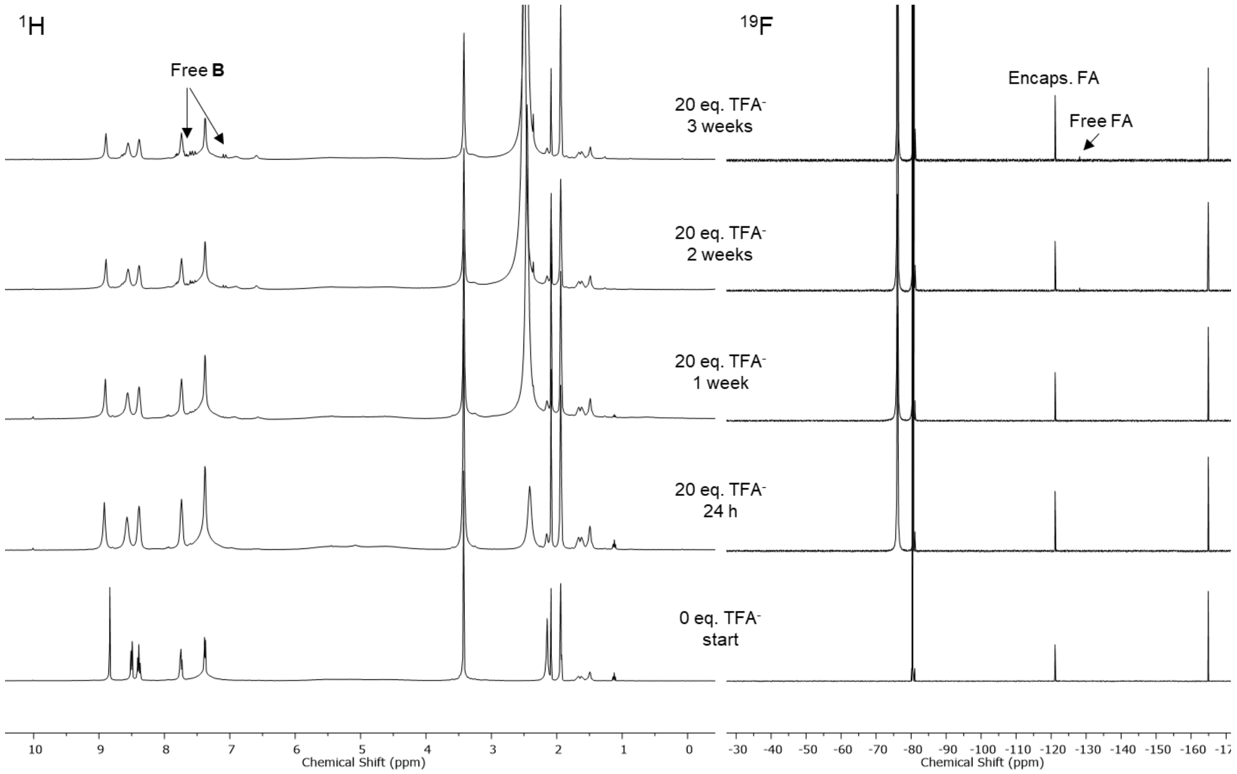

Figure S38: ^1H NMR spectra (400 MHz, 298 K, CD_3CN) (left) and ^{19}F NMR spectra (376 MHz, 298 K, CD_3CN) (right) of FA ⊂ **2** over three weeks after addition of 20 eq. of KTFA.

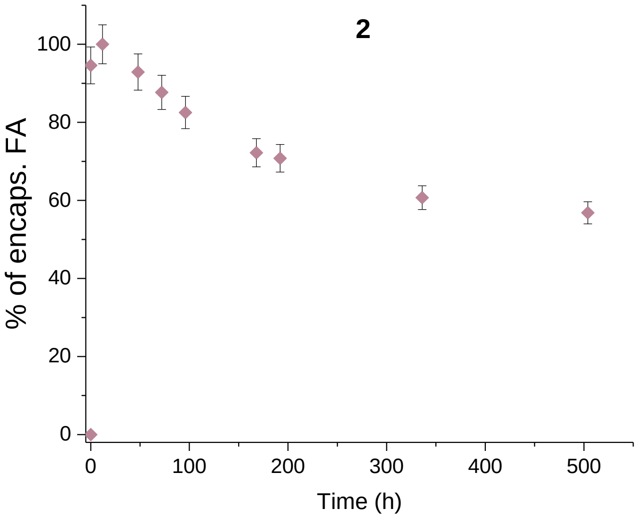

Figure S39: Amount of encapsulated FA in **1** in solution as a function of time.

Precipitate was observed to form over time and a small amount of free FA was observed after 2 weeks, which is consistent with the drop in intensity of the encapsulated FA. The free FA observed was attributed to a slight decomposition of **2** over time, whereas the precipitate and the important drop in the encapsulated FA observed was attributed to **2** precipitating out of solution as the TFA^- complex. This shows that **2** is reasonably stable in the presence of 20 eq. of KTFA in solution on short time scales but precipitation and a small amount of decomposition occurs over longer periods.

*5.1.2) Stability of **1** and **2** in the gel*

To 0.35 mL of a 5 mM solution of cage **1** or FA ⊂ **2** in CD_3CN was added 10 equivalents (12.5 mg, 17.5 µmol) of the peptide. The samples were sonicated for 10 min to promote the formation of the gel. The encapsulated guest peak in the ^{19}F NMR spectra was monitored over time and integrated against the NTf_2^- peak. The values were normalised so that the highest point (at 336 h for **1** and 12 h for **2**) is equal to 100% as only the host-guest complex was observed at this stage.

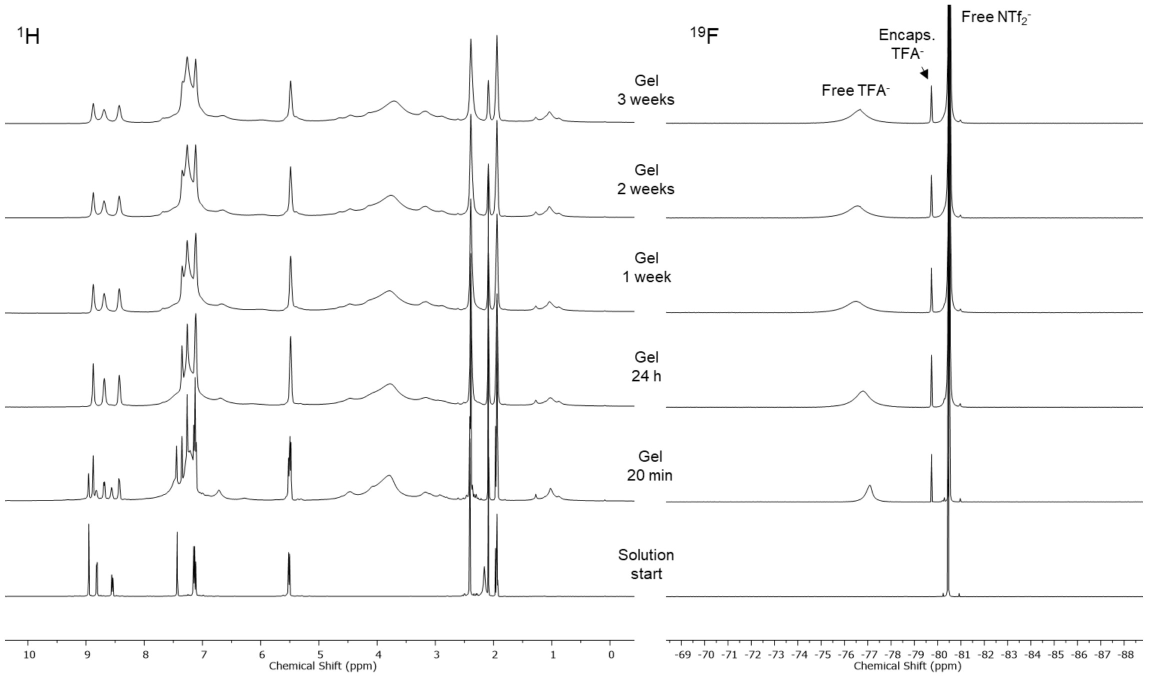

Figure S40: ^1H NMR spectra (500 MHz, 298 K, CD_3CN) (left) and ^{19}F NMR specta (471 MHz, 298 K, CD_3CN) (right) of TFA^- ⊂ **1** ⊂ Gel over three weeks after formation of the gel.

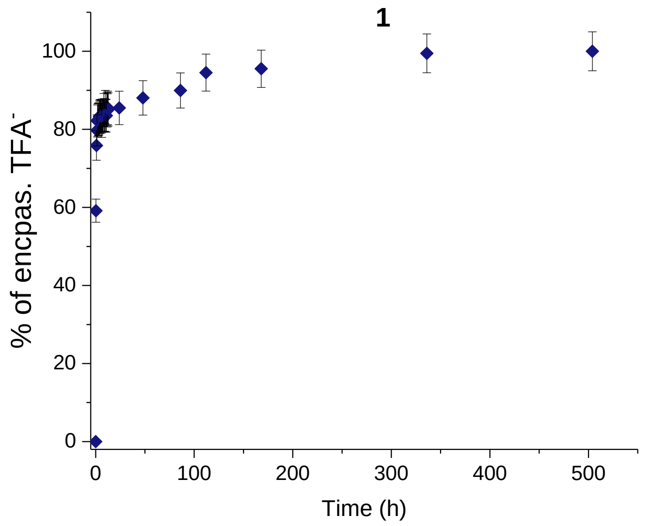

Figure S41: Amount of encapsulated TFA^- in **1** in the gel as a function of time.

The initial increase in the encapsulated TFA^- peak is due to the slower kinetics of encapsulation of TFA^- in the gel which is shown by the presence of both free and filled cage **1** at low times. This can be explained by the TFA^- interacting with the peptide fibrils and therefor being less available for encapsulation. No drop of the intensity was observed after maximum encapsulation was reached, demonstrating the stability of **1** in the gel.

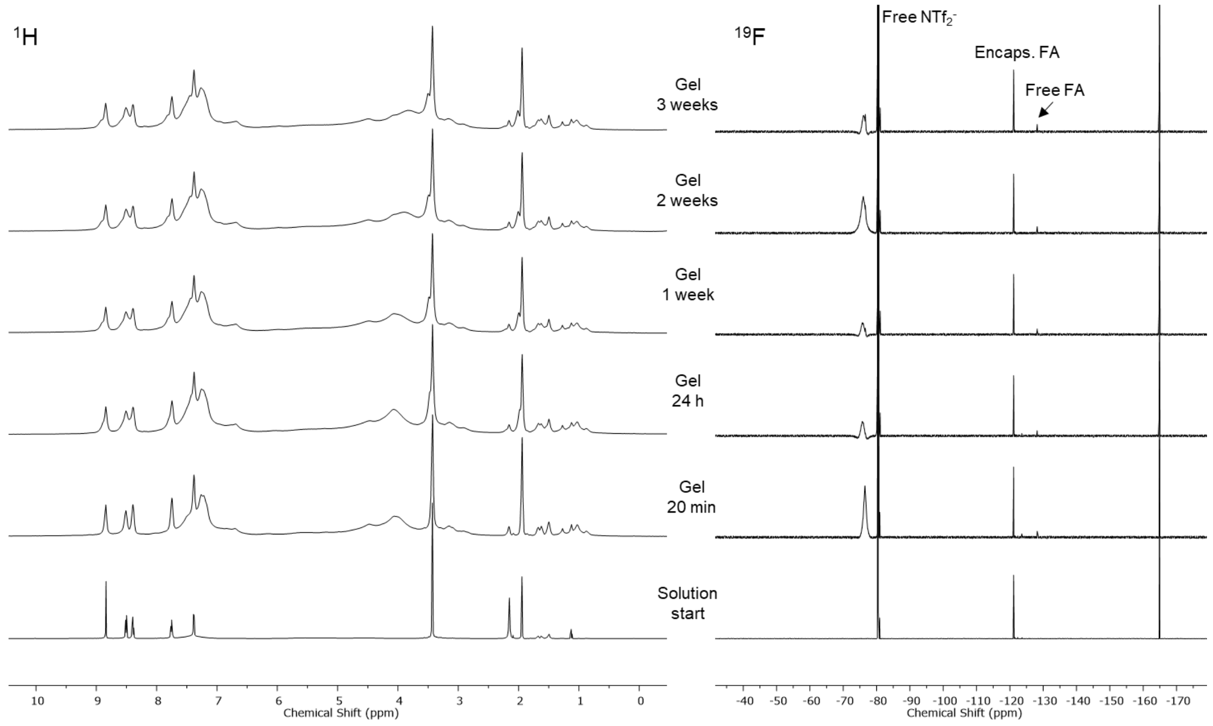

Figure S42: ^1H NMR spectra (500 MHz, 298 K, CD_3CN) (left) and ^{19}F NMR spectra (471 MHz, 298 K, CD_3CN) (right) of FA ⊂ **2** ⊂ Gel over three weeks after addition formation of the gel.

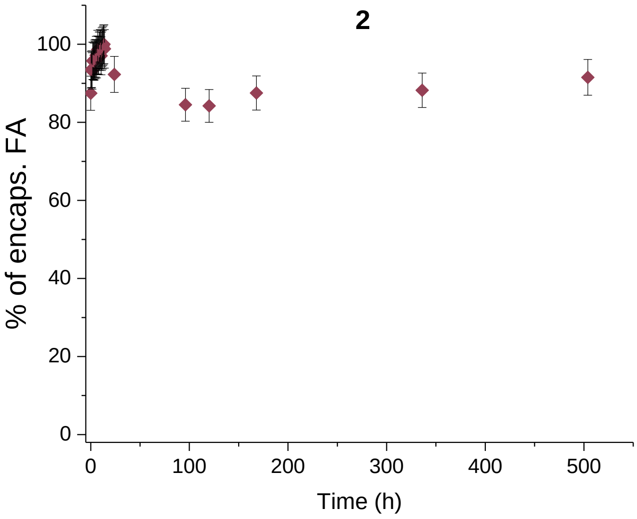

Figure S43: Amount of encapsulated FA in **2** in the gel as a function of time.

Slight decomposition (approx. 7%) of **2** upon formation of the gel is evidenced by the release of FA. No subsequent increase in the amount of free FA demonstrates the stability of **2** in the gel after the initial drop. Furthermore, no decrease of the encapsulated FA peak was observed after the initial drop, suggesting that **2** is more stable in the gel compared to the solution state. This can be explained by the TFA^- interacting with the gel fibrils and therefore not associating with the cage, which causes precipitation.

Furthermore, the presence of intact cage in the gel could be evidenced by ESI-MS after dissolving some gel containing either cage **1** or **2** in acetonitrile.

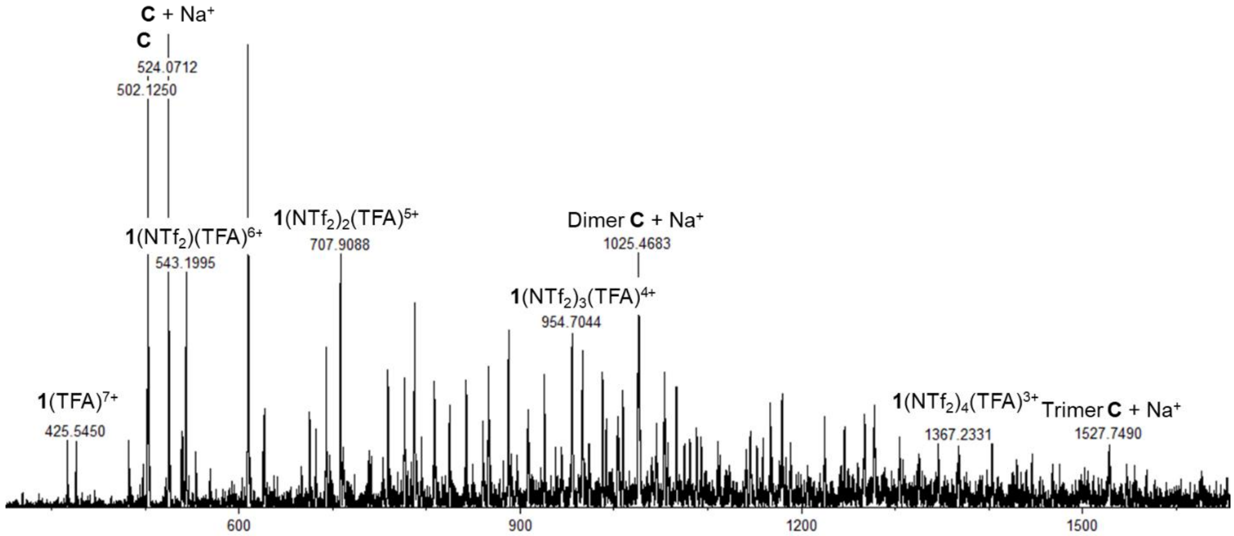

Figure S44: LR-ESI-MS of **1** and the peptide (labelled **C** for ease) after dissolving a sample of **1** ⊂ Gel in CH_3CN.

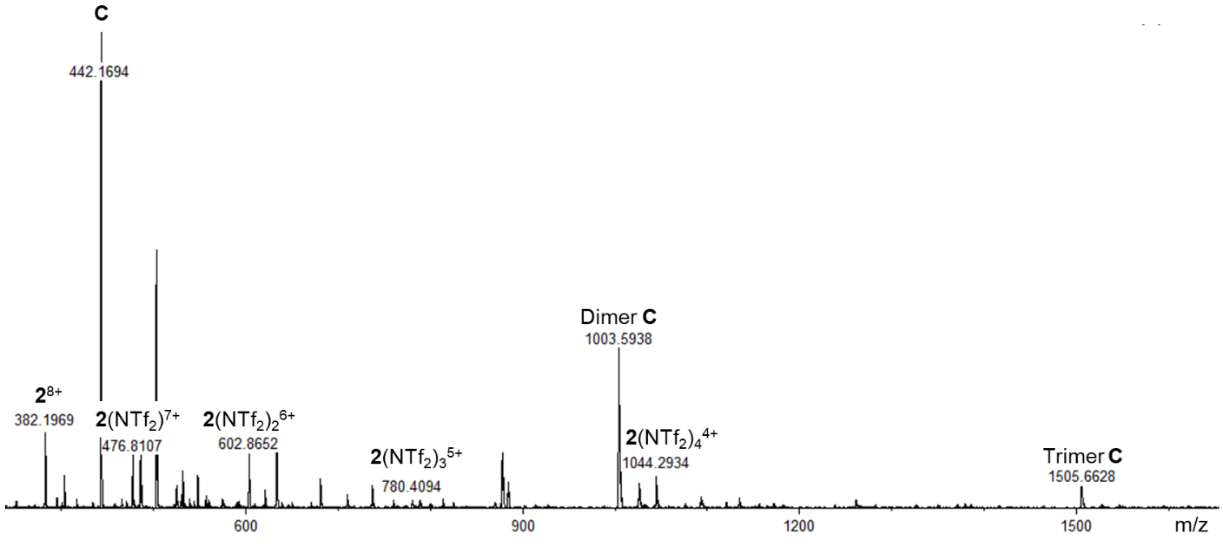

Figure S45: LR-ESI-MS of **2** and the peptide (labelled **C** for ease) after dissolving a sample of **2** ⊂ Gel in CH_3CN.

*5.1.3) Subcomponent exchange in **1***

Aniline **C** (5.2 mg, 17.5 µmol, 10 eq.) was added to a 5 mM solution of MOC **1** (8.9 mg, 1.75 µmol, 1 eq.) containing KTFA (5.3 mg, 35 µmol, 20 eq.). The sample was monitored over time and compared against the spectra of TFA⁻⊂**3** to evidence aniline exchange.

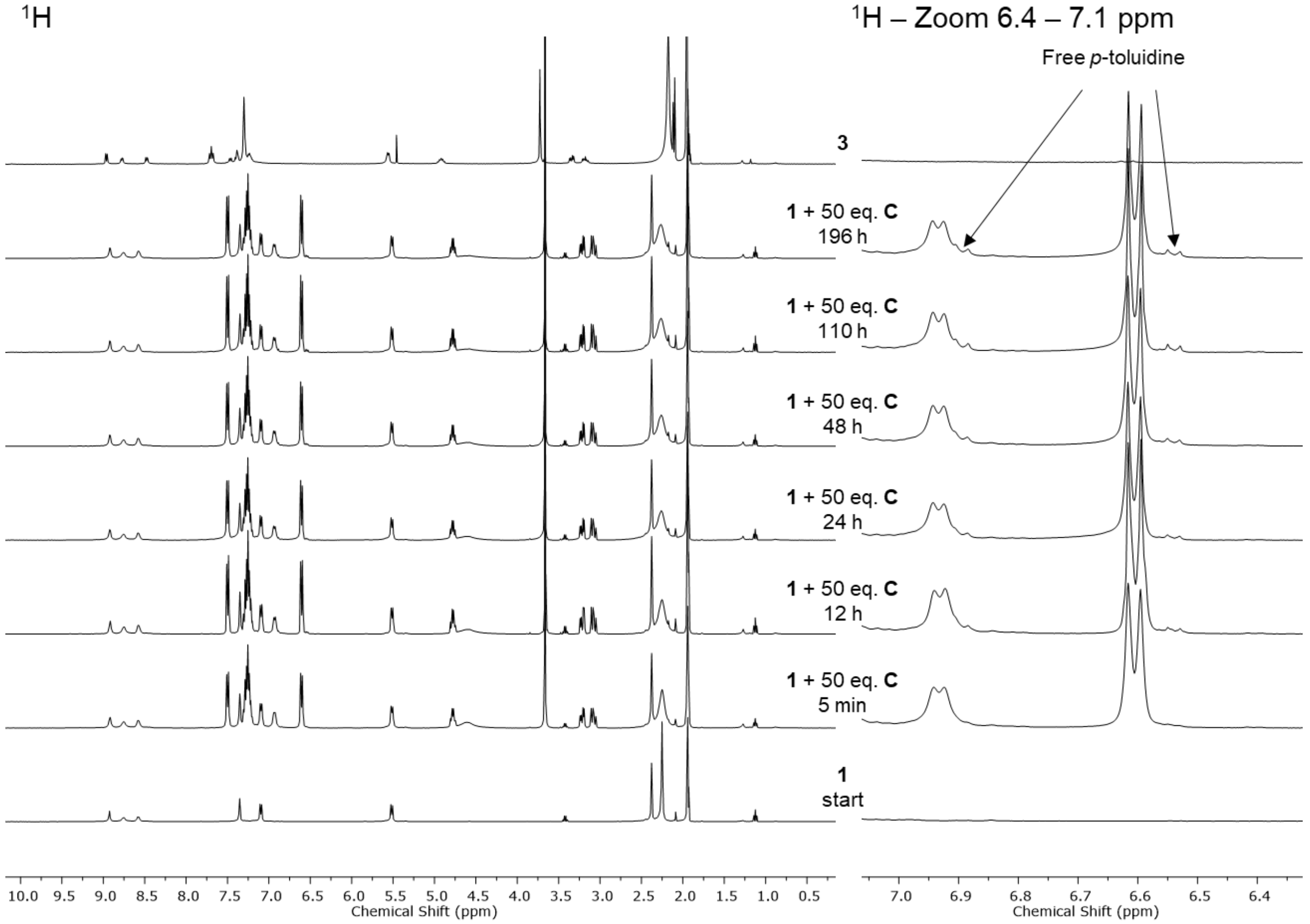

Figure S46: ^1H NMR spectra (500 MHz, 298 K, CD_3CN) (left) and zoom in the 6.4 - 7.1 ppm region (right) of **1** in the presence of 20 eq. of KTFA over 1 week after the addition of 10 eq. of **C.**

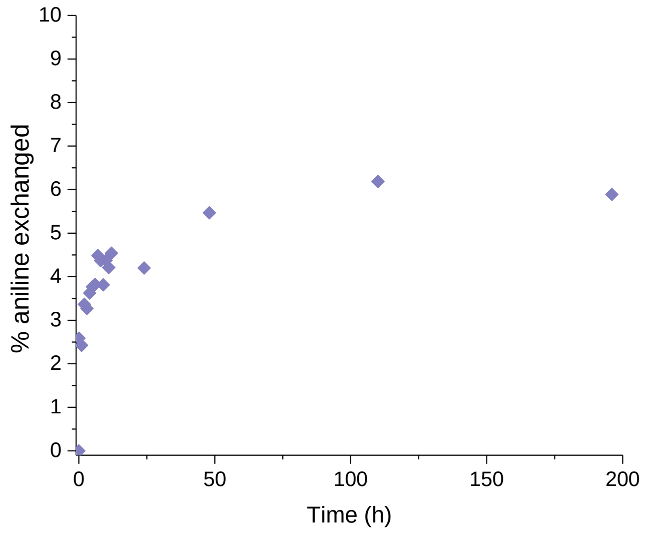

Figure S47: Percentage of aniline exchanged in **1** in the presence of 10 eq. of **C** in solution as a function of time.

No signals corresponding to cage **3** were observed after 1 week. This was explained by the small amount of aniline exchanged and the likely desymmetrisation of the signals in the species incorporating **C**. However, small signals corresponding to free *p*-toluidine in solution appeared over time. The maximum exchange (6%) was reached after 4 days. We concluded that, due to the electron withdrawing character of the amide, nucleophilic attack of **C** onto **1** was limited at the concentrations used in our study. Furthermore, we consider this case to be the “worst case scenario” as we expect the equilibriation in the gel to be slower, as has been seen for guest uptake. We also expect the anilines to be less available for nucleophilic attack in the gel than in solution as they are involved in the gel fibrils. By extension, we expect an absolute maximum of 6% of the *p*-toluidine to be exchanged for the tripeptide in **1**, which represents less than one molecule per cage.

5.2) Guest uptake in cages 1 and 2

5.2.1) Guest uptake in solution

To 0.35 mL of a 5 mM solution of cage **1** or **2** in CD_3CN was added 20 equivalents (35 µmol) of KTFA dissolved in 20 µL of CD_3CN. This aims at replicating the amount of TFA^- counter-ions present in solution due to the peptide. After 2 h of equilibration, 1 equivalent (1.75 µmol) of TBA ReO4 or FA in 20 µL of CD_3CN was respectively added to the solution of **1** or **2**. The encapsulated guest peak (and the free guest peak in the case of FA) in the ^{19}F NMR spectra were monitored over time and integrated against the triflimide peak. The values for **1** were normalised so that the value for the encapsulated TFA^- is equal to 100% before the addition of TBA ReO_4 as only the host-guest complex $TFA^- \subset$ **1** is present at this stage. The drop in the intensity of the encapsulated TFA^- is attributed to the encapsulation of ReO_4^- and therefore the values for the encapsulated ReO_4^- were assumed the complement of the values for the TFA^-. The values for **2** were normalised so that the sum of the signal for free and encapsulated FA was equal to 100%.

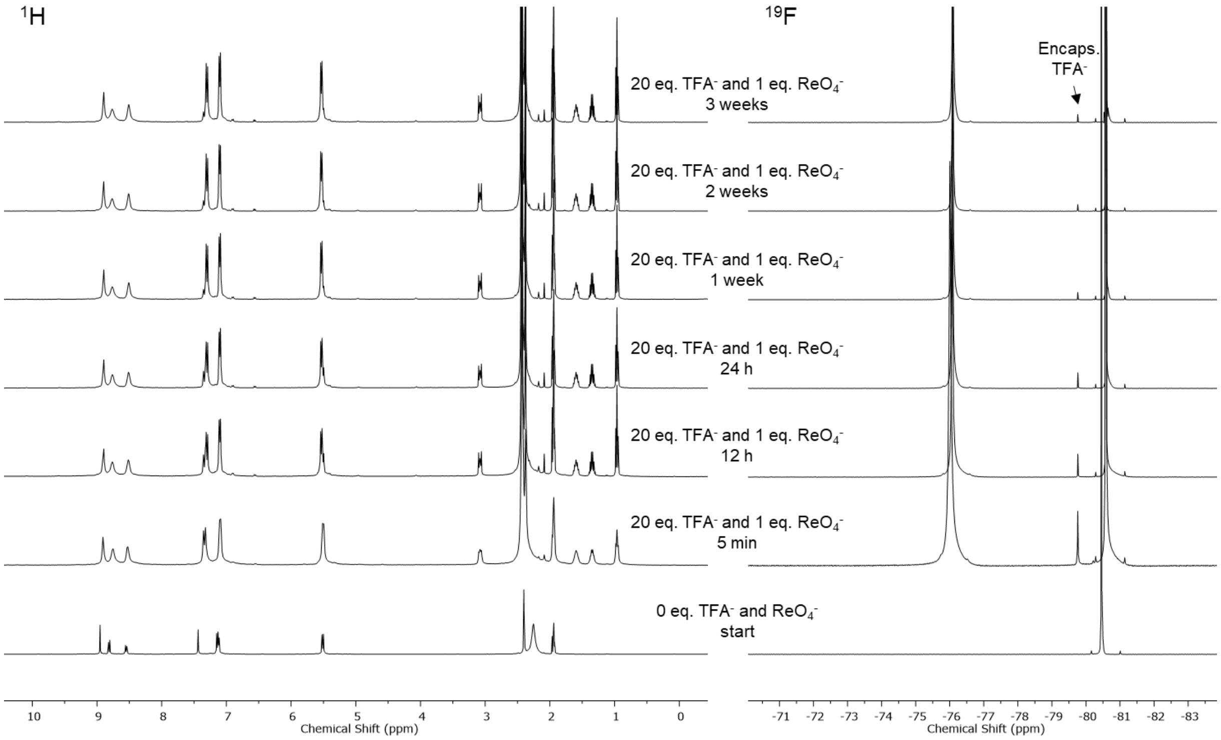

Figure S48: ^{1}H NMR spectra (400 MHz, 298 K, CD_3CN) (left) and ^{19}F NMR spectra (376 MHz, 298 K, CD_3CN) (right) of **1** in solution over three weeks after addition of 20 eq. of KTFA and 1 eq. of TBA ReO_4.

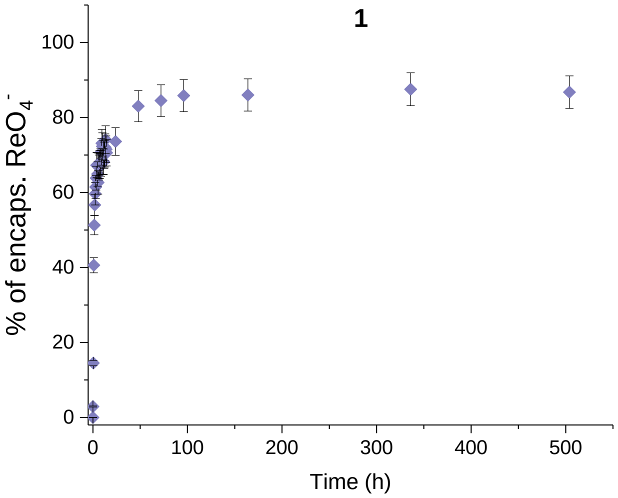

Figure S49: Amount of encapsulated ReO_4^- in **1** in the presence of 20 eq. TFA^- in solution as a function of time.

The maximum encapsulation of ReO_4^- (86% ± 4) was reached after four days. The process followed an exponential asymptotic model but was too fast to allow calculation of a rate of encapsulation in this case.

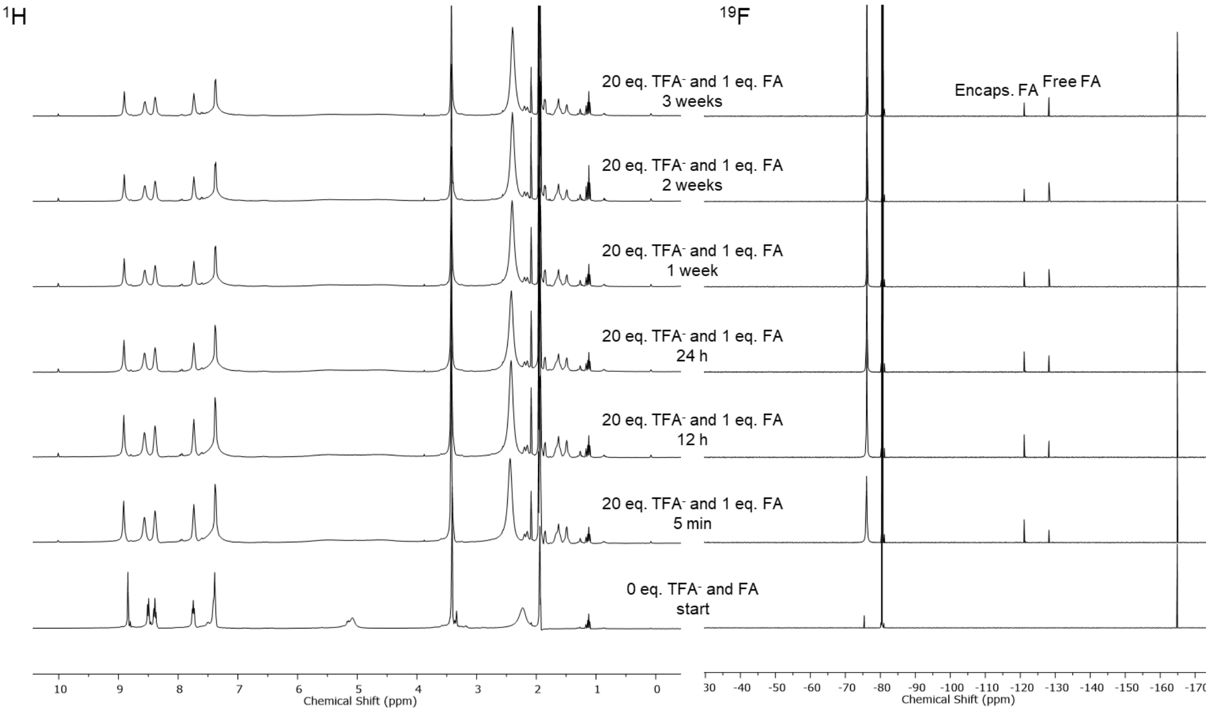

Figure S50: ^{1}H NMR spectra (400 MHz, 298 K, CD_3CN) (left) and ^{19}F NMR spectra (376 MHz, 298 K, CD_3CN) (right) of **2** over three weeks after addition of 20 eq. of KTFA and 1 eq. of FA.

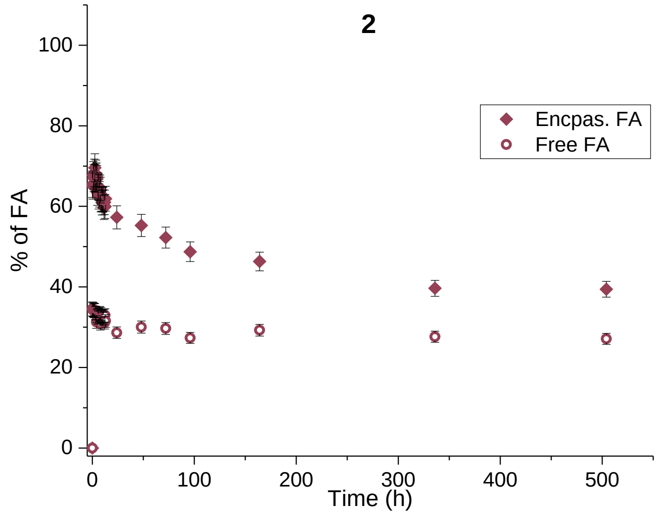

Figure S51: Amount of encapsulated FA in **2** and free FA in the presence of 20 eq. TFA^- in solution as a function of time.

The maximum encapsulation of FA (69% ± 3) was reached within 2 h of the guest addition. The process was too fast to allow calculation of a rate of encapsulation in this case. The subsequent drop in the encapsulated FA followed the same trend as FA ⊂ **2** in solution and was attributed to the precipitation of **2** as the TFA^- adduct as no increase in the free FA indicative of cage decomposition could be observed.

5.2.2) Guest uptake in gel

To 0.35 mL of a 5 mM solution of cage **1** or **2** in CD_3CN was added 10 equivalents (12.5 mg, 17.5 µmol) of the peptide. The samples were sonicated for 10 min to promote the formation of the gel. After 2 h of equilibration for **2** and 3 weeks for **1** (in this case the guest uptake experiment was performed on the same gel sample used to test the stability of cage **1**), 1 equivalent (1.75 µmol) of TBA ReO4 or FA in 20 µL of CD_3CN was respectively layered on top of the gels containing **1** or **2**. The encapsulated TFA^- peak for **1**, and the

encapsulated NTf_2^-, encapsulated FA and free FA for **2** in the ^{19}F NMR spectra were monitored over time and integrated against the NTf_2^- peak. The values for **1** were normalised so that the value for the encapsulated TFA^- is equal to 100% before the addition of TBA ReO_4 as only the host-guest complex $TFA^- \subset$ **1** is present at this stage. The drop in the intensity of the encapsulated TFA^- is attributed to the encapsulation of ReO_4^- and therefore the values for the encapsulated ReO_4^- were assumed the complement of the values for the TFA^-. The values for **2** were normalised so that the maximal sum of the signal for the free and encapsulated FA (after 1 week, once all the guest had diffused through the gel) was equal to 100%.

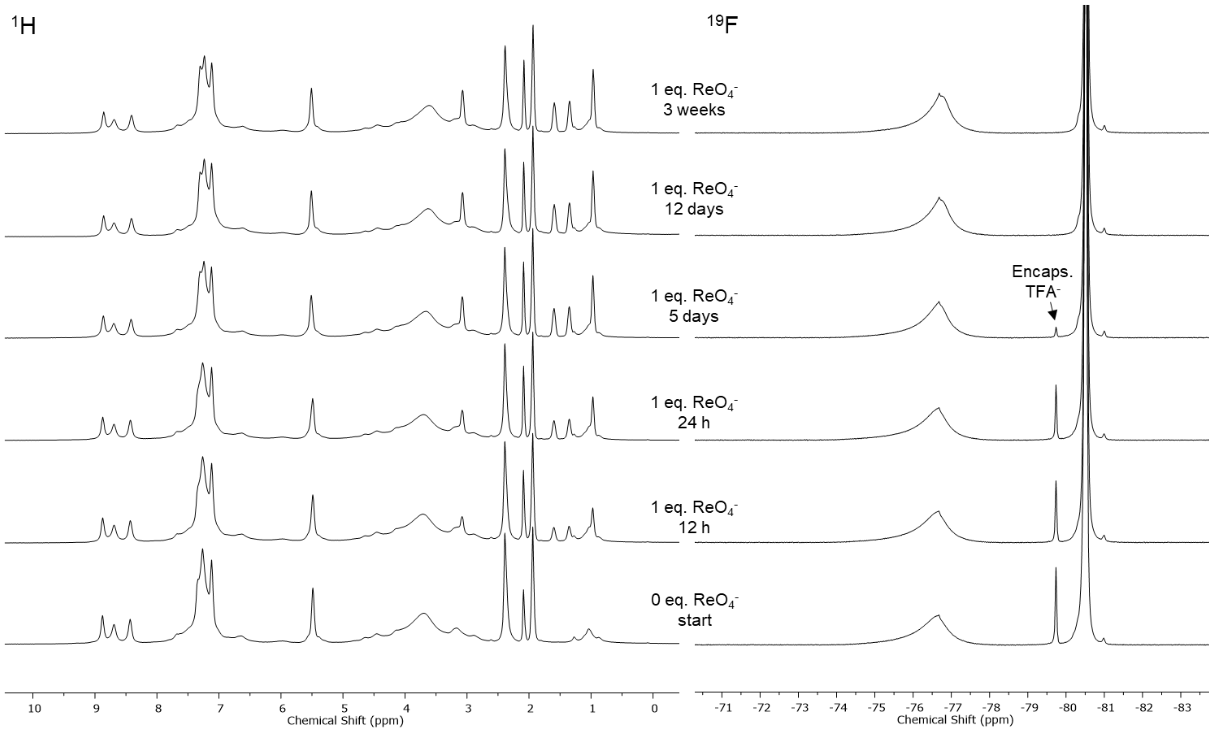

Figure S52: ^1H NMR spectra (500 MHz, 298 K, CD_3CN) (left) and ^{19}F NMR spectra (471 MHz, 298 K, CD_3CN) (right) of **1** $\subset$ Gel over three weeks after addition of 1 eq. of TBA ReO_4.

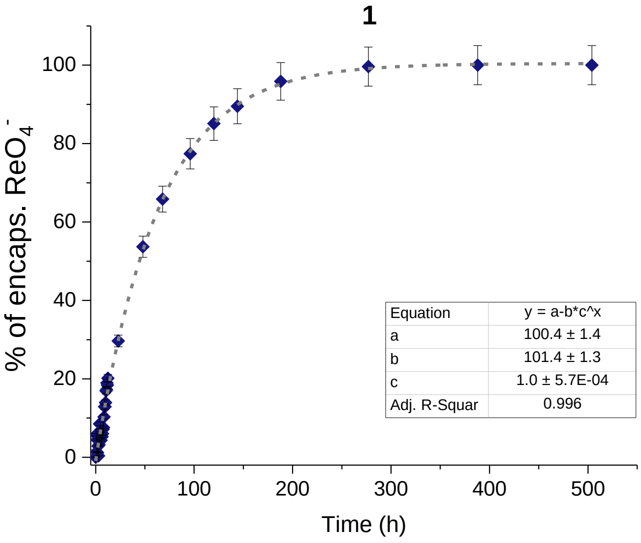

Figure S53: Amount of encapsulated ReO_4^- in **1** in the gel as a function of time (blue diamonds) and exponential asymptotic fitting (dotted line).

Complete encapsulation of ReO_4^- was reached after 12 days. The process followed an exponential asymptotic model:

$$\%\, encaps.guest = a - b * c^x$$

The initial rate of encapsulation was given by the formula:[7]

$$k_{ini} = -b * ln(c)$$

giving the rate of encapsulation for ReO_4^- : $k_{iniReO4}$ = 1.6 ± 0.05 %.h^{-1}

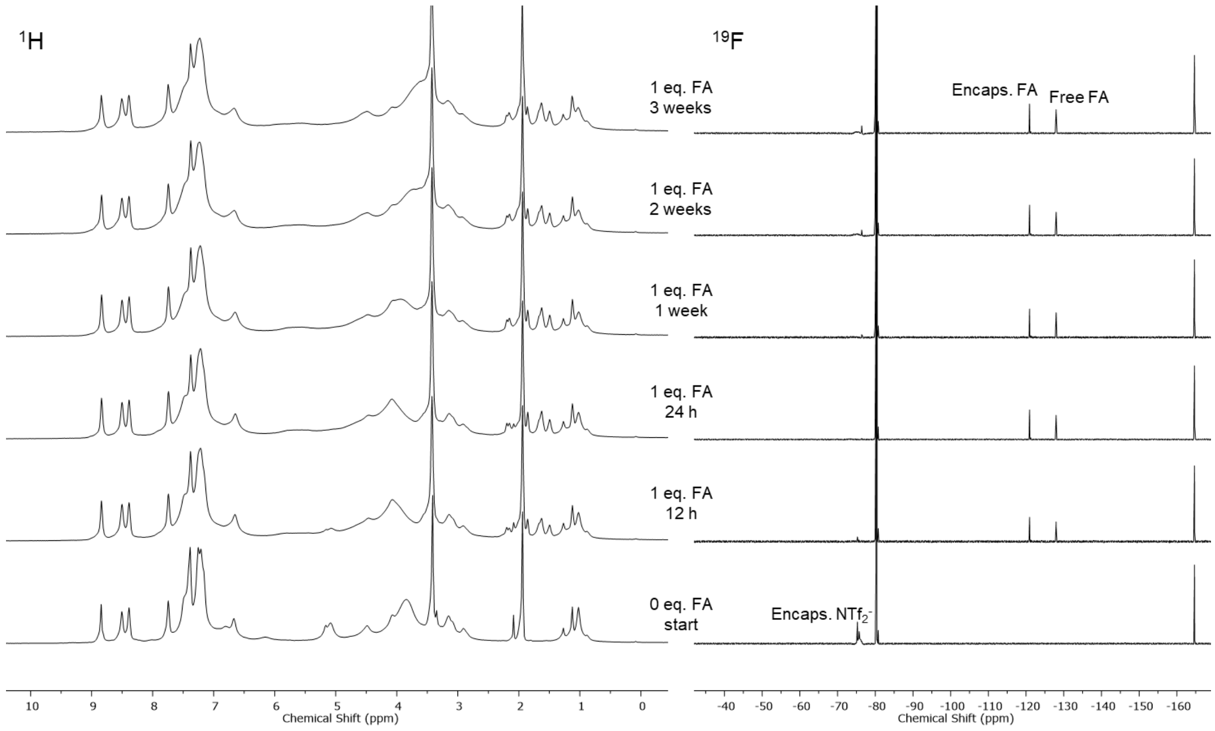

Figure S54: ^{1}H NMR spectra (500 MHz, 298 K, CD_3CN) (left) and ^{19}F NMR spectra (471 MHz, 298 K, CD_3CN) (right) of **2** ⊂ Gel over three weeks after addition of 1 eq. of FA.

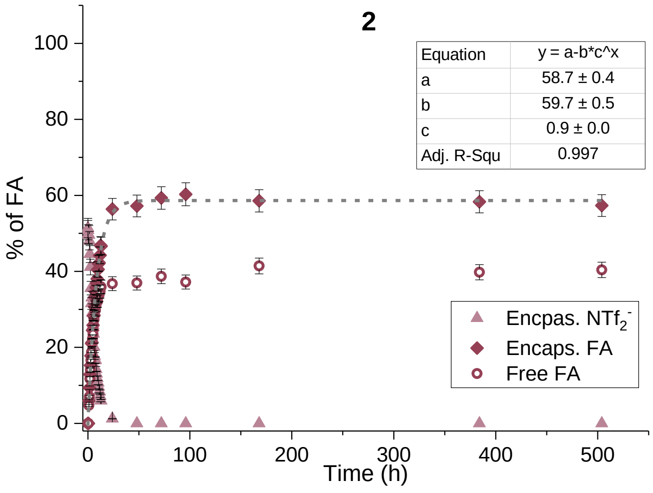

Figure S55: Amount of encapsulated FA in **2** (purple diamonds), encapsulated NTf_2^- (pink triangles) and free FA (empty circles) in the gel as a function of time and exponential asymptotic fitting for the encapsulated FA (dotted line).

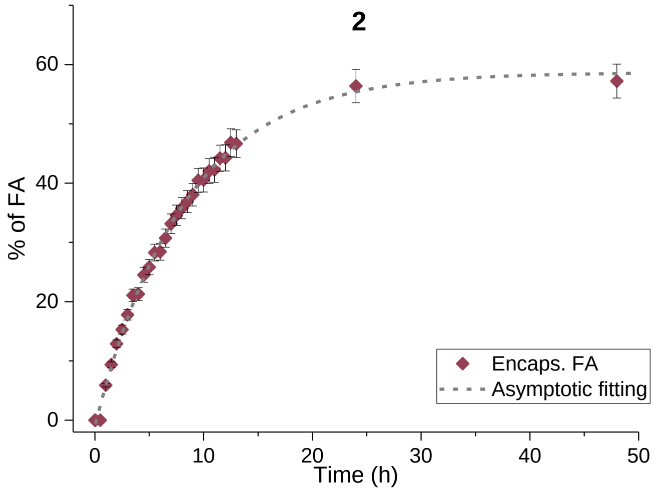

Figure S56: Zoom of the 0-50 h region showing the amount of encapsulated FA in **2** in the gel as a function of time and exponential asymptotic fitting (dotted line).

The encapsulated NTf_2^- dropped rapidly, with no trace of encapsulation for this compound after 48 h. Maximum encapsulation of FA (59% ± 3) was reached after 72 h. The process followed an exponential asymptotic model. The calculated rate of encapsulation is k_{iniFA} = 7.23 ± 0.15 %.h^{-1}

5.3) Diffusion of compounds through the gel

5.3.1) Diffusion of respective guest

The diffusion of guests was followed over time by following the evolution of their signals in the NMR spectra. The diffusion curve of TBA ReO_4 in the gel made of the peptide and cage **1** was obtained by monitoring the signal for the TBA^+ at 1.6 ppm in the ^1H NMR spectra integrated against the acetonitrile peak. Values were normalized so that the maximum diffusion after 3 weeks was equal to 100%. The diffusion curve of the FA in the gel made of the peptide and cage **2** was obtained by summing the integrals for the free and encapsulated FA relative to the NTf_2^- peak. Values were normalized so that the maximal sum of the signal for the free and encapsulated FA (after 1 week) was equal to 100%.

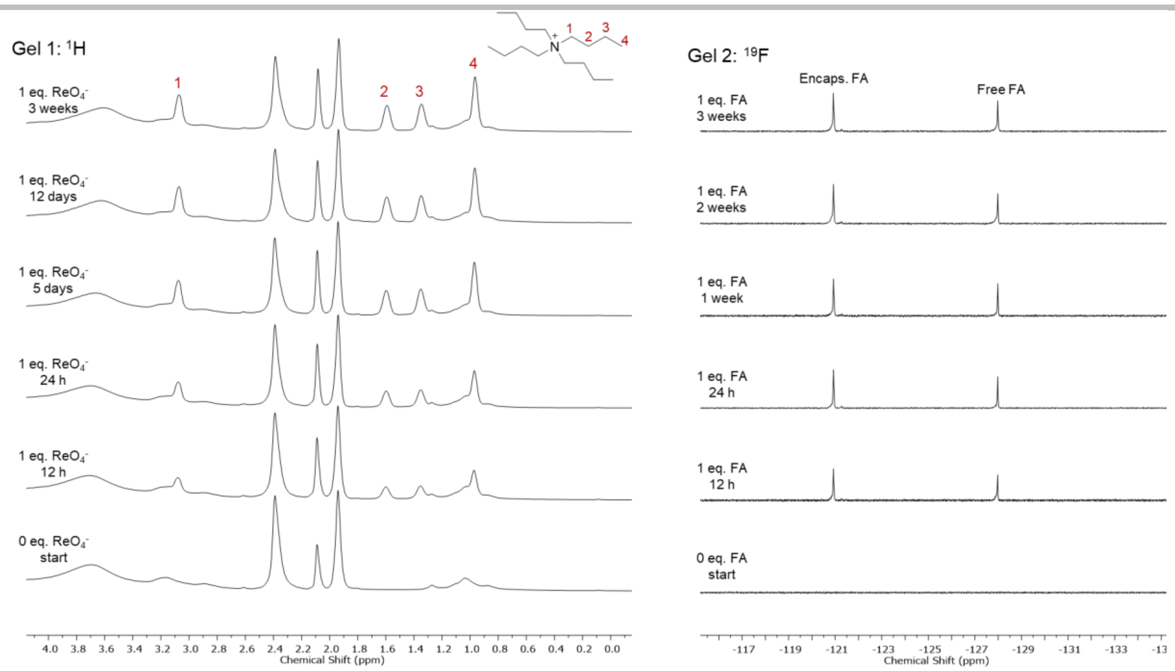

Figure S57: ^{1}H NMR spectra (500 MHz, 298 K, CD_3CN) (left) zoomed in the 0-4 ppm region of **1** ⊂ Gel over three weeks after addition of 1 eq. of TBA ReO_4^- showing the evolution of the TBA$^+$ peak. ^{19}F NMR spectrum (471 MHz, 298 K, CD_3CN) (right) zoomed in the -116 − -134 ppm region of **2** ⊂ Gel over three weeks after addition of 1 eq. of FA showing the evolution of both the encapsulated and free FA.

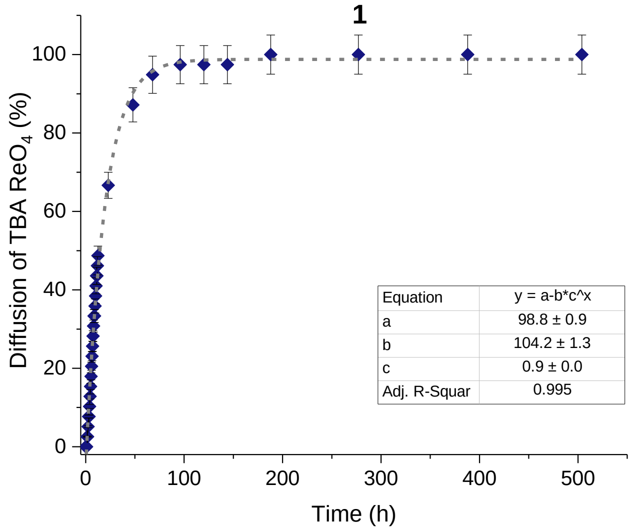

Figure S58: Diffusion of TBA ReO_4 in the gel containing **1** as a function of time (blue diamonds) and exponential asymptotic fitting (dotted line).

Diffusion of TBA ReO_4 reached a steady regime after 5 days. The process followed an exponential asymptotic model. The calculated rate of diffusion is $k_{iniReO4/diff1}$ = 5.44 ± 0.20 %.h^{-1}

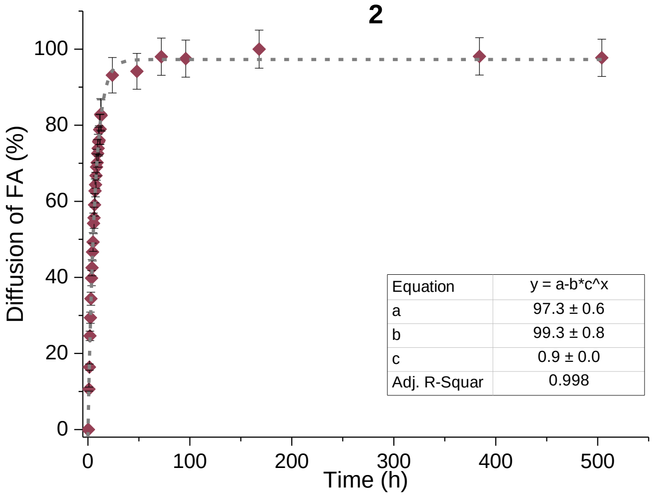

Figure S59: Diffusion of FA in the gel containing **2** as a function of time (purple diamonds) and exponential asymptotic fitting (dotted line).

Diffusion of FA reached a steady regime after 2 days. The process followed an exponential asymptotic model. The calculated rate of diffusion is $k_{iniFA/diff2}$ = 14.61 ± 0.28 %.h^{-1}.

5.3.2) *Diffusion of FA in **1** ⊂ Gel*

To compare the diffusion of compounds between gels containing **1** and **2**, 1 equivalent of FA (1.75 µmol) in 20 µL of CD_3CN was layered on top of a sample of gel containing **1**. The values of the integral of the free FA peak against NTf_2^- were normalized so that the maximum value after 3 weeks was equal to 100%.

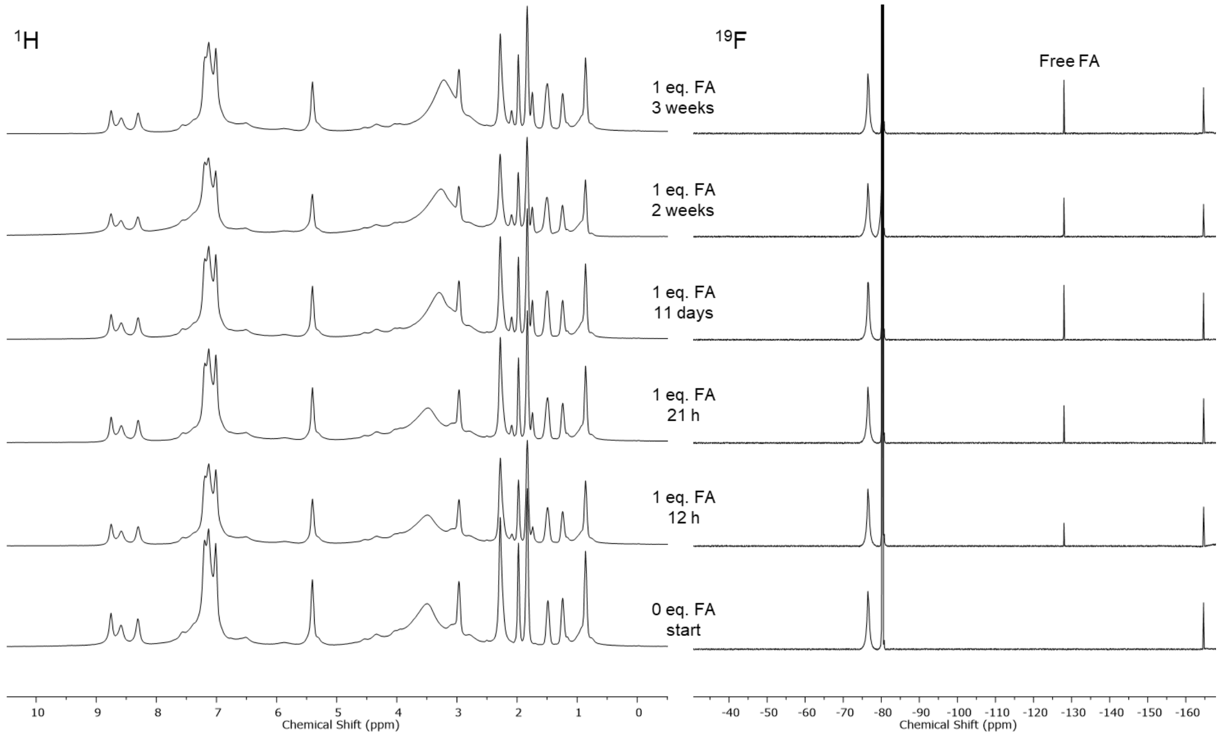

Figure S60: ^{1}H NMR spectra (500 MHz, 298 K, CD_3CN) (left) and ^{19}F NMR spectra (471 MHz, 298 K, CD_3CN) (right) of ReO_4^- ⊂ **1** ⊂ Gel over three weeks after addition of 1 eq. of FA showing the evolution of the free FA peaks.

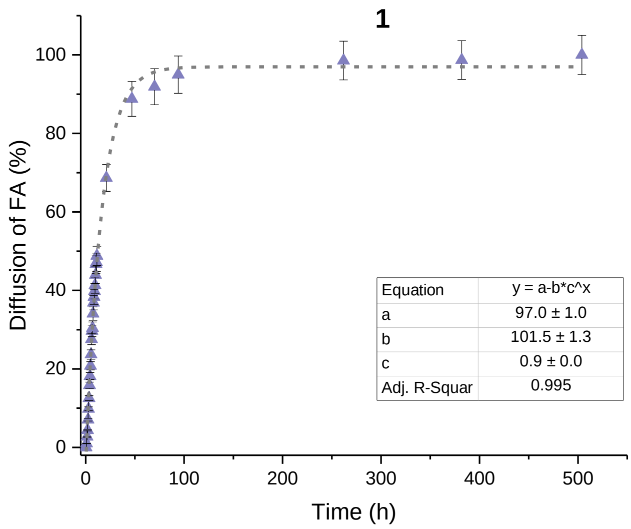

Figure S61: Diffusion of FA in the gel containing **1** as a function of time (blue triangles) and exponential asymptotic fitting (dotted line).

Diffusion of FA reached a steady regime after 5 days. The process followed an exponential asymptotic model. The calculated rate of diffusion is $k_{iniFA/diff1}$ = 6.30 ± 0.22 %.h^{-1}

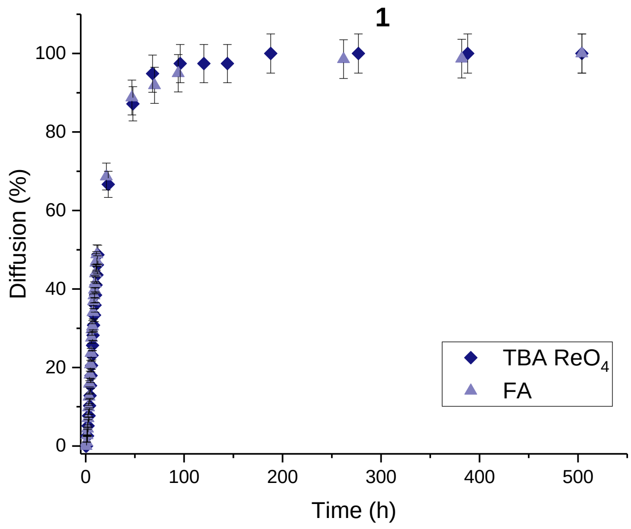

Figure S62: Compared diffusion of TBA ReO_4 (dark blue diamonds) and FA (light blue triangles) in the gel containing **1** as a function of time.

The diffusion follows similar kinetics for both the TBA ReO_4 and the FA in the gel containing **1**, indicating that the rate of diffusion isn't influenced by the type of compound as long as they are of similar size.

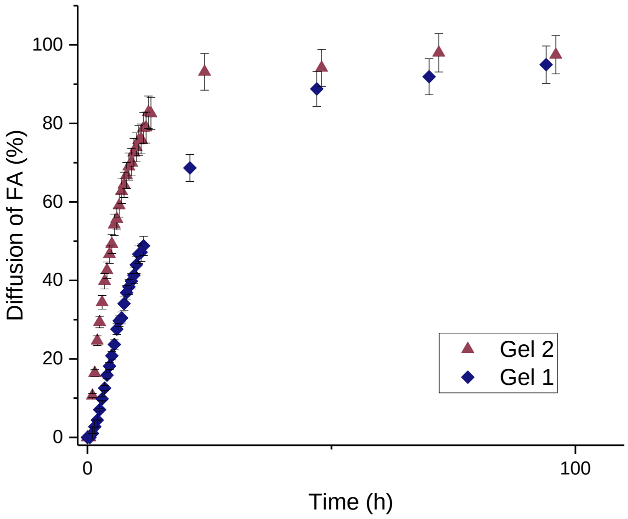

Figure S63: Compared diffusion of FA in the gel containing **1** (dark blue diamonds) and in the gel containing **2** (purple triangles) as a function of time. Only the range 0-100 h is shown for clarity.

The diffusion follows different kinetics for the FA in the gels containing **1** and **2**, indicating that the rate of diffusion is influenced by the type of cage incorporated in the gel. This is consistent with the gel having different structures when cage **1** or **2** are incorporated, an observation which is also supported by the rheology results.

5.3.3) Cage diffusion in the gels

50 µL of a 1 mM solution of $TFA^{-}\subset$**1** or FA⊂**2** (0.35 µmol) in CD_3CN was layered on top of a preformed sample of gel (peptide concentration 50 mM) containing 1.75 µmol of FA⊂**2** or $ReO_4^{-}\subset$**1** respectively. The diffusion curves were obtained by monitoring the integrals of the encapsulated TFA^{-} peak for 1 and the encapsulated FA for 2 over time in the ^{19}F NMR spectra. The values were integrated against the NTf_2^{-} peak and normalised so that the value for the encapsulated guest was equal to 100% after 3 weeks.

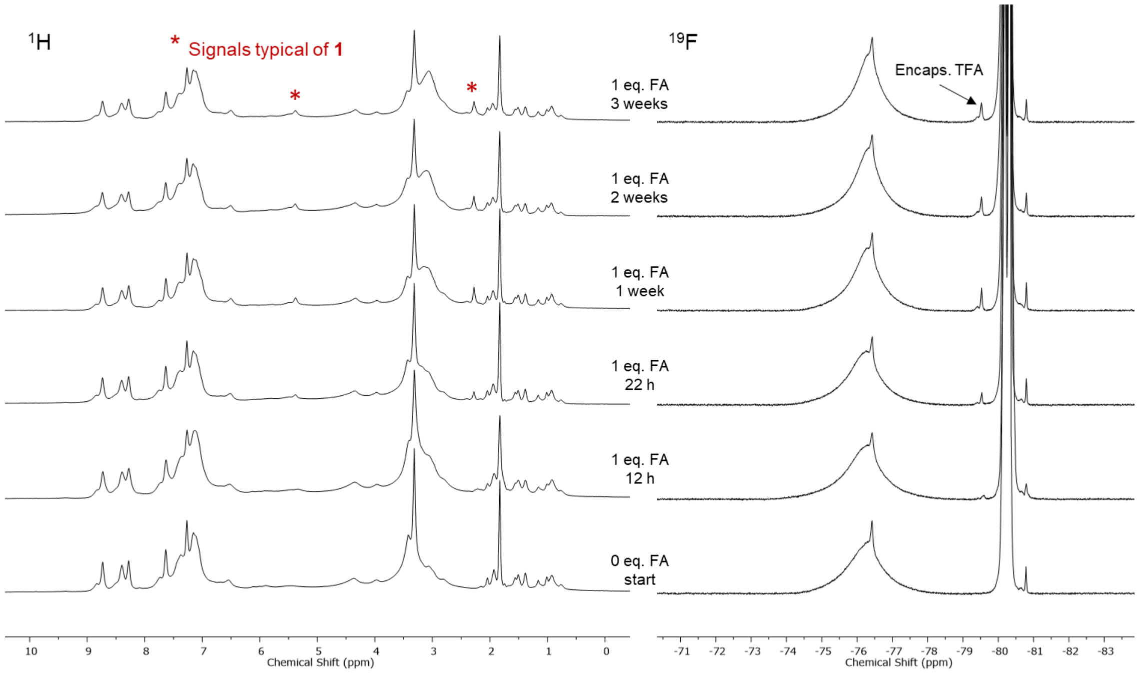

Figure S64: ^{1}H NMR spectra (500 MHz, 298 K, CD_3CN) (left) and ^{19}F NMR spectra (471 MHz, 298 K, CD_3CN) (right) of FA ⊂ **2** ⊂ Gel over three weeks after addition of 1 eq. of TFA^{-} ⊂ **1**.

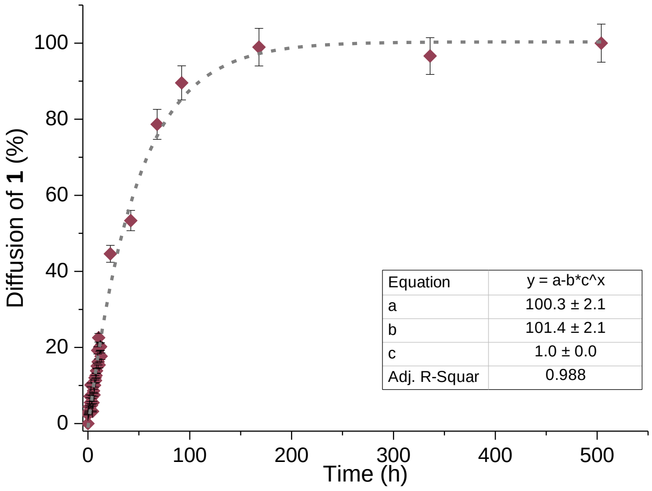

Figure S65: Diffusion of TFA⊂**1** in the gel containing **2** as a function of time (purple diamonds) and exponential asymptotic fitting (dotted line).

Diffusion of TFA⊂**1** reached a steady regime after 1 week. The process followed an exponential asymptotic model. The calculated rate of diffusion is $k_{ini1/diff2}$ = 2.11 ± 0.11 %.h^{-1}.

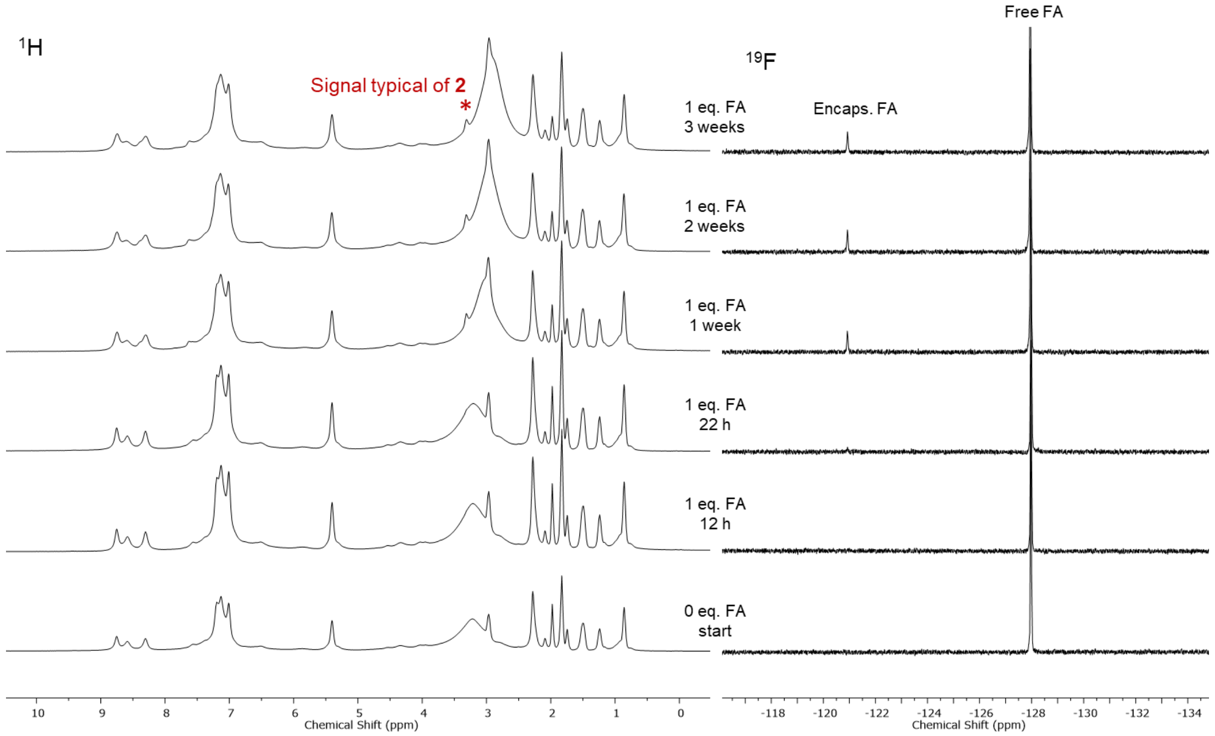

Figure S66: ^{1}H NMR spectra (500 MHz, 298 K, CD_3CN) (left) and ^{19}H NMR spectra (471 MHz, 298 K, CD_3CN) (right) of **1** ⊂ Gel over three weeks after addition of 1 eq. of FA ⊂ **2**.

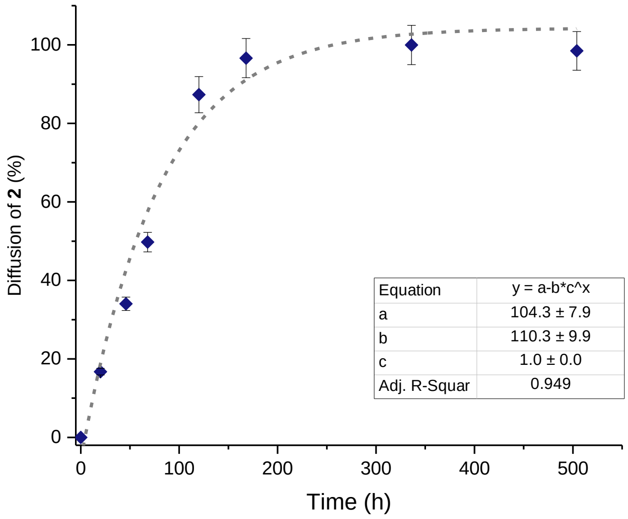

Figure S67: Diffusion of FA⊂**2** in the gel containing **1** as a function of time (blue diamonds) and exponential asymptotic fitting (dotted line).

Diffusion of FA⊂**2** reached a steady regime after 10 days. The process followed an exponential asymptotic model. The calculated rate of diffusion is $k_{ini2/diff1}$ = 1.31 ± 0.20 %.h^{-1}.

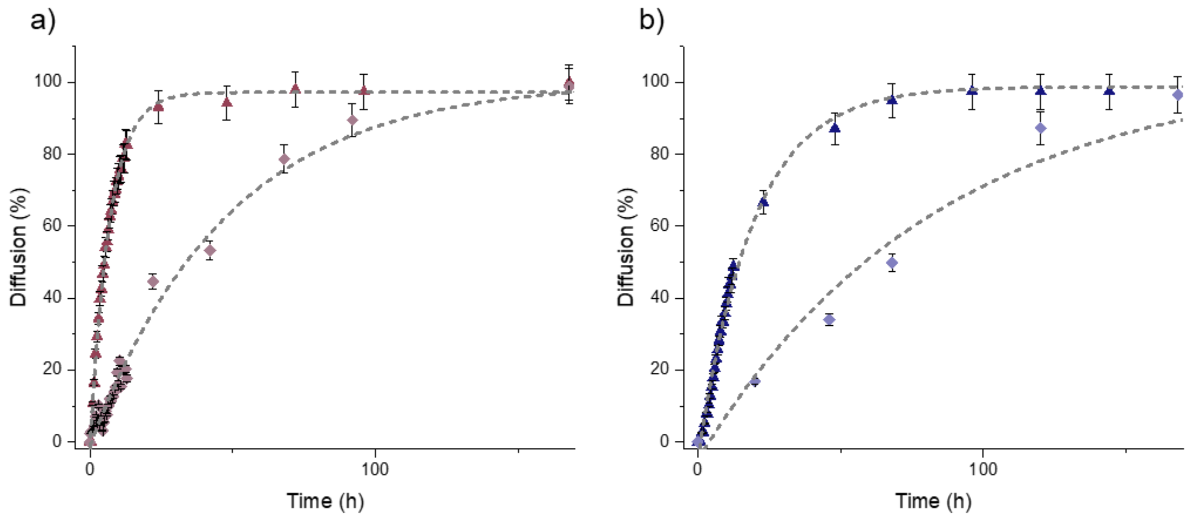

Figure S68: A comparison of the diffusion of a) FA in the gel containing **2** (dark purple triangles) vs. **1** in the gel containing **2** (pink diamonds) and b) TBA ReO_4^- in the gel containing **1** (dark blue triangles) vs. **2** in the gel containing **1** (light blue diamonds) as a function of time. Only the range 0-170 h is shown for clarity.

The diffusion follows different kinetics for the guests and the cages in the gel containing **1** or **2**. The diffusion of the guest is much faster than of the cage, showing the suitability of this system for separation on timescale shorter than a week. Indeed, the guests will diffuse and become encapsulated in their respective layers before significant leaching of the cages into each other's layer happens.

5.3.4) Summary of initial uptake and diffusion rates

Table S5. Initial rate of uptake and diffusion for ReO_4^-, FA, 1 and 2 in 1⊂Gel and 1⊂Gel derived from kinetic ^1H and ^{19}F NMR experiments.

	Initial rate of uptake (%.h^{-1})		Initial rate of diffusion (%.h^{-1})			
	ReO_4^-	FA	ReO_4^-	FA	**1**	**2**
**1⊂Gel**	1.60 ± 0.05		5.44 ± 0.20	6.30 ± 0.22		1.31 ± 0.20
**2⊂Gel**		7.23 ± 0.15		14.61 ± 0.28	2.11 ± 0.11	

5.4) Spatial segregation of cages and guest separation

The set up for slice selective NMR was similar to the one previously reported.[2]

To 0.120 mL of a 5 mM solution of cage **1** in CD_3CN was added 10 equivalents (4.4 mg, 6.0 µmol) of the peptide. The solution was carefully transferred in a 5 mm NMR tube and was sonicated for 10 min to promote the formation of the gel. After 2 h of equilibration, 0.09 mL of a 50 mM solution of the peptide (3.2 mg, 4.3 µmol) in CD_3CN was layered on top of the gel containing **1**. The sample was sonicated for 10 min in order to promote the formation of the gel. This layer serves as a buffer between the two cages. To 0.120 mL of a 5 mM solution of cage **2** in CD_3CN was added 10 equivalents (4.4 mg, 6.0 µmol) of the peptide. The solution was carefully layered on top of the sample and was sonicated for 10 min to promote the formation of the gel. The final layered gel system is shown in Figure S63.

Initial ^1H NMR 2D mapping was done by acquiring 20 slices of approximately 1 mm thickness to obtain a high resolution spatial representation of the sample, in order to check for the presence of each cages within their respective layers. The 'map' of the sample produced allows the slices with the best homogeneity and signal intensity to be chosen for the monitoring of the process.

Shaped pulse excitation was the Bruker standard gaussian-cascade G4 using:

NS = 8

D1 = 1.00 s

SPW1 = 0.30642

P11 = 1494

GPZ1 = 23.5%

^1H and ^{19}F NMR 2D mapping of the sample were used to localize the offset corresponding to each layer of gel under the conditions (slice width) used for the 1D experiments. Fewer and thicker slices were used (approx. 2 mm, 10 slices) than in the initial 2D mapping in order to get better intensity of the signals. The mapping was repeated with the following parameters:

^1H NMR:

GPZ1 = 6%, SPW1 = 0.30642, P11 = 1494, NS = 8

^{19}F NMR:

GPZ1 = 11.5%, SPW1 = 4.792, P11 = 795.2, NS = 8

The optimal slices showing most intense signal in the ^1H were identified for each layer of gel and the SPOFFS1 were recorded:

Top layer: SPOFFS1 = 9200 Bottom layer: SPOFFS1 = -9200

The SPOFFS1 being dependent of the frequency of excitation and the probe used, the SPOFFS1 for the ^{19}F NMR were identified by matching the slices, giving:

Top layer: SPOFFS1 = 9200 Bottom layer: SPOFFS1 = -18400

In order to keep the performance of the ^{19}F experiments similar to ^1H, a 20ppm spectral width was acquired and offset selected to observe each region of interest. One region of the ^1H NMR (0 – 10 ppm, NS = 8) and two regions of the ^{19}F NMR (−135 to − 115 ppm and −88 to − 68 ppm, NS = 256) were monitored. It is thus not possible to treat the ^{19}F data quantitatively, as the peaks could not be

integrated against a reference signal. It was also necessary to acquire 256 scans per spectrum to enable detection of the guest signals, which prevented us from acquiring well-defined 2D ^{19}F NMR maps of the two regions of interest (−135 to − 115 ppm and −88 to − 68 ppm). Such 2D maps would have taken approximately six hours each to acquire and would therefore not have been representative of the state of the system at a single point in time. However, relative values can be obtained by comparing the absolute values of the integrals of peaks between different time points in the 1D slice-selective ^{19}F NMR as the gain and number of scans was kept identical between runs.

Once the slice selective experimental parameter was optimized, 1 equivalent (0.60 µmol) of a mixture of TBA ReO4 and FA in 20 µL of CD_3CN was then layered on top of the sample. The peaks corresponding to encapsulated TFA^-, encapsulated FA and free FA in the ^{19}F NMR spectra were monitored over time.

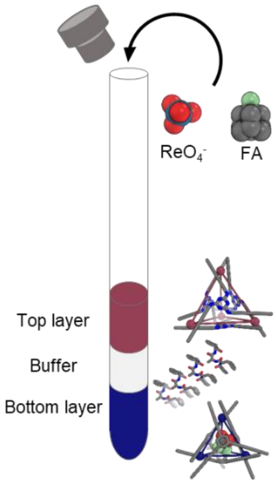

Figure S69: Composition of the sample for the slice selective NMR experiment.

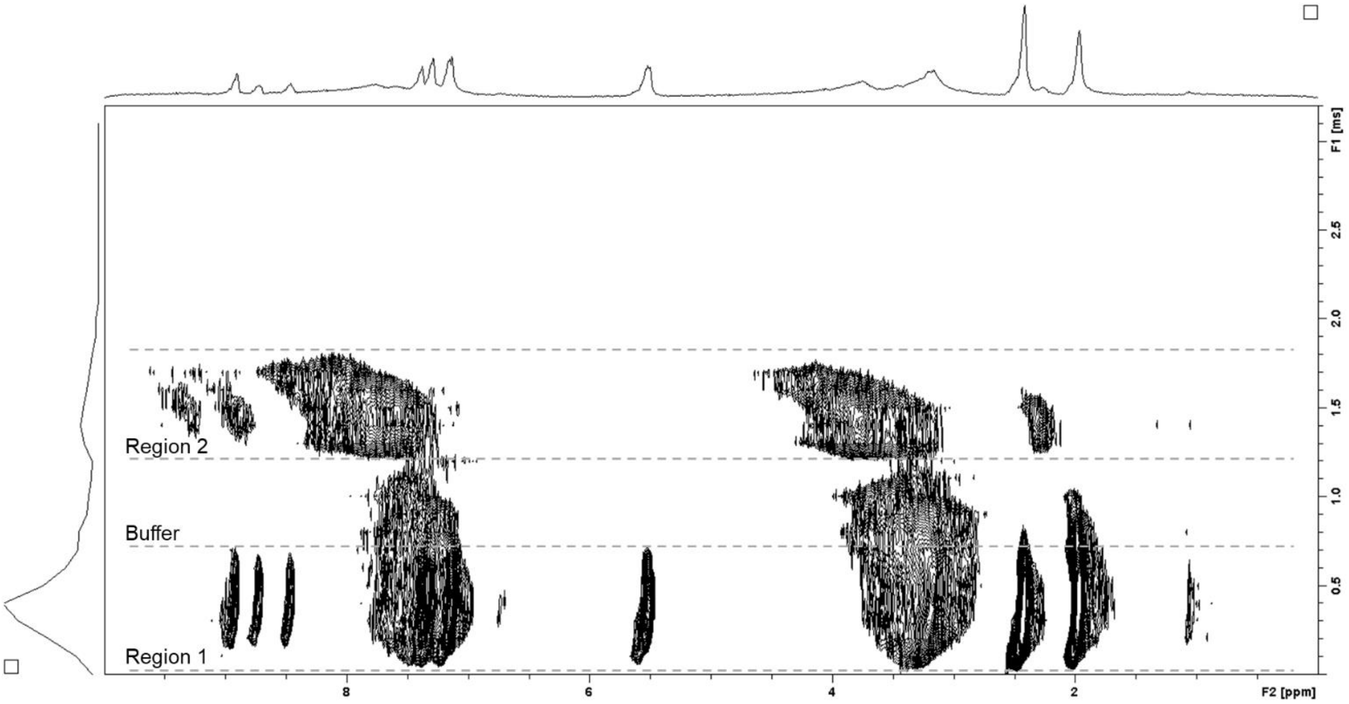

Figure S70: 2D ^1H NMR map of the gel sample showing three regions containing **1** (bottom), the peptide only (middle) and **2** (top).

A signal/noise ratio value of *S/N*=19 was obtained for the MOCs (5 mM) in the gel under the conditions used to acquire the 2D map from the 1D spectra extracted from the 2D map. In the ideal case where *S/N*=3, the maximum corresponding concentration of MOC present in buffer would be 0.8 mM. However, detection can be achieved as long as *S/N*>1, which in this case gives a maximum concentration of 0.26 mM. We thus estimated that the concentration of MOC in the buffer was below 0.26 mM, meaning less than 10% of the MOC had leached in the buffer.

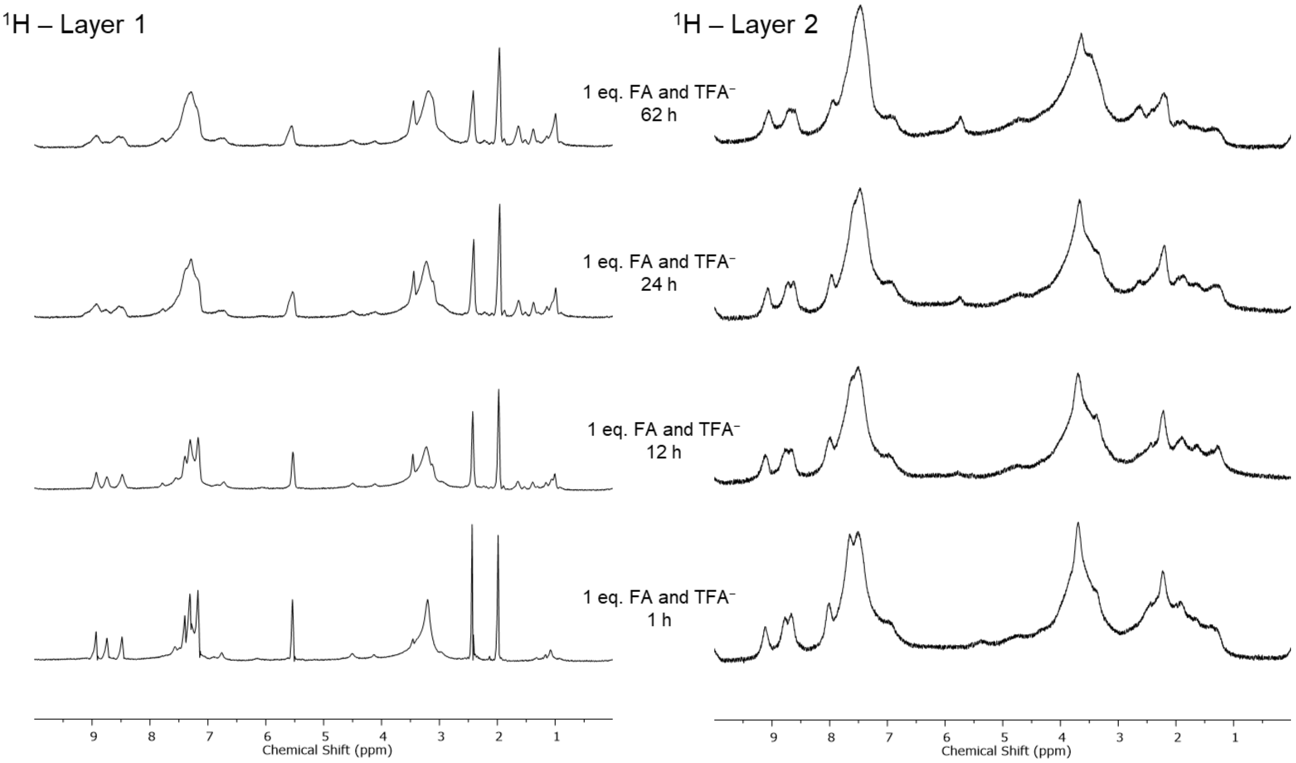

Figure S71: ^{1}H NMR spectra (500 MHz, 298 K, CD_3CN) of the bottom layer containing **1** (left) and of the top layer containing **2** (right) over 62 h after addition of 1 eq. of FA and TFA^- mixed.

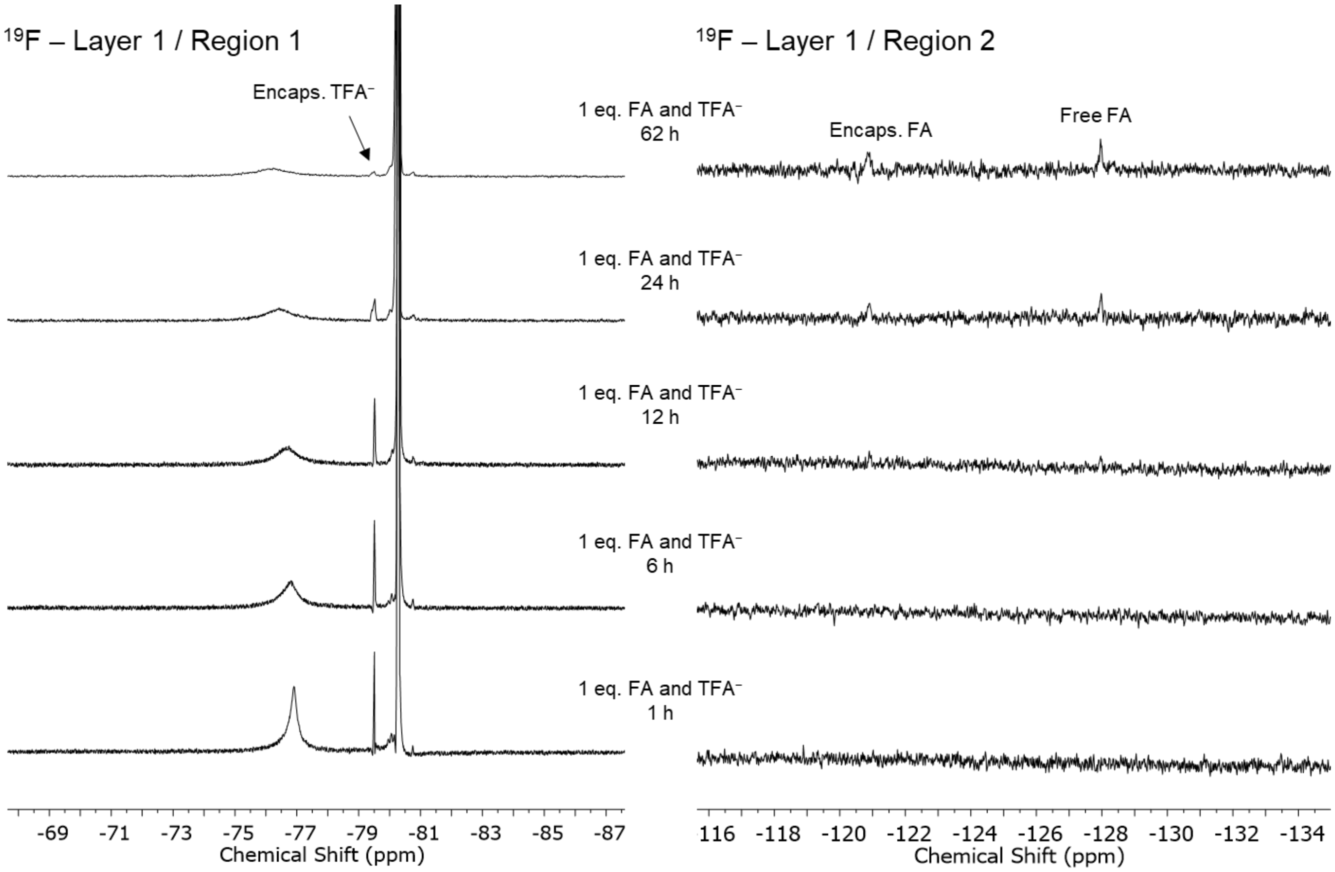

Figure S72: ^{19}F NMR spectra (471 MHz, 298 K, CD_3CN) of the bottom layer containing **1** over 62 h after addition of 1 eq. of FA and TFA^- mixed in the two regions where signals for the guests are seen (TFA^- on the left and FA on the right).

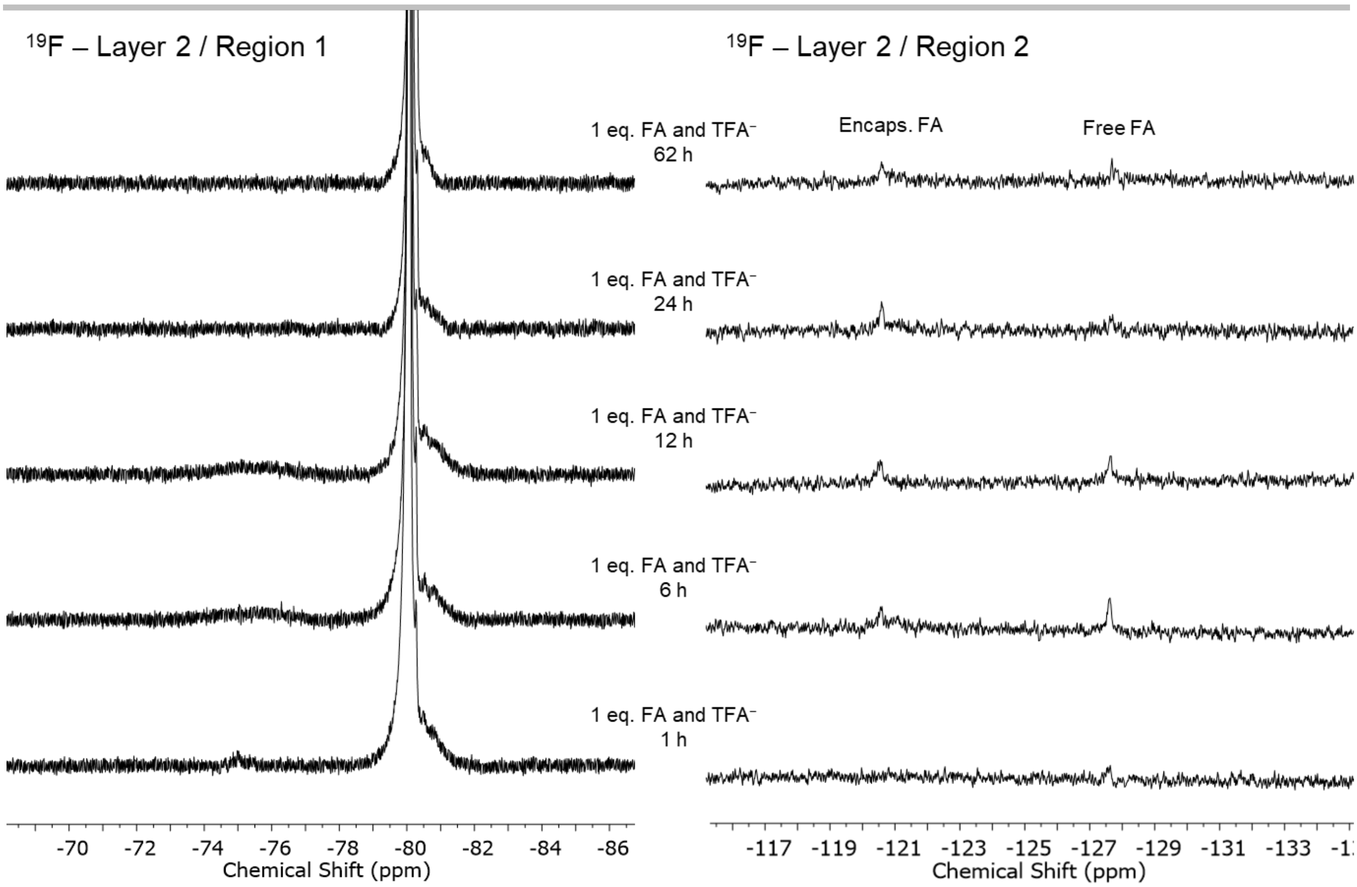

Figure S73: ^{19}F NMR spectra (471 MHz, 298 K, CD_3CN) of the top layer containing **2** over 62 h after addition of 1 eq. of FA and TFA^- mixed in the two regions where signals for the guests are seen (TFA^- on the left and FA on the right).

After addition of the mixture of guests on top of the tri-layered sample, we observe the appearance of the signals corresponding to the encapsulated FA in the top layer containing **2**. Free FA was observed in both layers 1 and 2, which we infer to be a result of the weaker binding of this guest in cage **2**. We note that no comparisons between the intensity of the signals observed in both layers could be made, as the performance of the detection greatly varied from one layer to another, evidenced by the higher signal/noise ratio in layer 2 compared to 1. However, layer 2 additionally presented signals corresponding to FA ⊂ **2**, which were only observed after 24 h in layer 1 due to the leaching of FA ⊂ **2**. We thus concluded that despite the lack of absolute separation of FA between layers, an enrichment of this compound was achieved in layer 2.

Similarly, we observe the disappearance of the encapsulated TFA^- peak in the layer containing **1**, indicative of the $ReO4^-$ encapsulation. As ReO_4^- is NMR silent, its presence in layer 2 could not be directly observed. However, 1 eq. of perrhenate per eq. of MOC **1** was added to the tri-layered system, and approximately 80% of ReO_4^- was observed to be encapsulated in **1**, as noted by following the displacement of TFA by ^{19}F NMR by integration against the triflimide peak. We infer that the remaining 20% would be entrapped in the gel (as opposed to encapsulated inside the MOC) and would become spread evenly between both layers. We thus conclude that, even though complete segregation of the ReO_4^- in layer **1** was not achieved, an estimated enrichment of up to a 9:1 ratio between layer 1 and layer 2 was obtained.

References:

[1] a) M. P. Sibi, G. Petrovic, *Tetrahedron: Asymmetry* **2003**, *14*, 2879-2882; b) A. M. Castilla, N. Ousaka, R. A. Bilbeisi, E. Valeri, T. K. Ronson, J. R. Nitschke, *J. Am. Chem. Soc.* **2013**, *135*, 17999-18006; c) J. L. Bolliger, T. K. Ronson, M. Ogawa, J. R. Nitschke, *J. Am. Chem. Soc.* **2014**, *136*, 14545-14553.

[2] A. B. Grommet, J. B. Hoffman, E. G. Percástegui, J. Mosquera, D. J. Howe, J. L. Bolliger, J. R. Nitschke, *J. Am. Chem. Soc.* **2018**, *140*, 14770-14776.

[3] A. M. Castilla, M. A. Miller, J. R. Nitschke, M. M. J. Smulders, *Angew. Chem. Int. Ed.* **2016**, *55*, 10616-10620.
[4] F. Stewart, *Aust. J. Chem.* **1983**, *36*, 1629-1638.
[5] A. B. Grommet, J. L. Bolliger, C. Browne, J. R. Nitschke, *Angew. Chem. Int. Ed.* **2015**, *54*, 15100-15104.
[6] a) B. Lekprasert, V. Korolkov, A. Falamas, V. Chis, C. J. Roberts, S. J. B. Tendler, I. Notingher, *Biomacromolecules* **2012**, *13*, 2181-2187; b) D. Iglesias, M. Melle-Franco, M. Kurbasic, M. Melchionna, M. Abrami, M. Grassi, M. Prato, S. Marchesan, *ACS Nano* **2018**, *12*, 5530-5538.
[7] N. Busschaert, S. J. Bradberry, M. Wenzel, C. J. E. Haynes, J. R. Hiscock, I. L. Kirby, L. E. Karagiannidis, S. J. Moore, N. J. Wells, J. Herniman, G. J. Langley, P. N. Horton, M. E. Light, I. Marques, P. J. Costa, V. Félix, J. G. Frey, P. A. Gale, *Chem. Sci.* **2013**, *4*, 3036-3045.

Author Contributions

AMG synthesized and characterized the peptide, carried out the tests to establish the condition of gelation and acquired and analyzed the rheometry, UV-vis and CD data (lead).
MK synthesized and characterized subcomponents and cages (equal), designed, acquired and analyzed the data on the host-guest studies in solution and gels (lead), designed (equal), acquired and analyzed the data on the spatial segregation experiment (lead).
CJEH synthesized and characterized subcomponents and cages (equal), acquired some of the data on the host-guest studies in solution and gels (supporting) and designed the spatial segregation experiment (equal).
SK acquired and analyzed the TEM data.
DI acquired and analyzed the Raman data.
The original draft was written by AMG and MK (equal) and the figures were done by MK. CJEH, DI, SK, JRN and SM all contributed to the manuscript at later stages.
JRN and SM administered the project and secured funding for the project.
